# Investigating the influence of masker and target properties on the dynamics of perceptual awareness under informational masking

**DOI:** 10.1371/journal.pone.0282885

**Published:** 2023-03-16

**Authors:** Alexandre Veyrié, Arnaud Noreña, Jean-Christophe Sarrazin, Laurent Pezard

**Affiliations:** 1 Aix-Marseille Université, LNC, CNRS UMR 7291, Marseille, France; 2 ONERA, The French Aerospace Lab, Salon de Provence, France; All India Institute of Speech and Hearing, INDIA

## Abstract

Informational masking has been investigated using the detection of an auditory target embedded in a random multi-tone masker. The build-up of the target percept is influenced by the masker and target properties. Most studies dealing with discrimination performance neglect the dynamics of perceptual awareness. This study aims at investigating the dynamics of perceptual awareness using multi-level survival models in an informational masking paradigm by manipulating masker uncertainty, masker-target similarity and target repetition rate. Consistent with previous studies, it shows that high target repetition rates, low masker-target similarity and low masker uncertainty facilitate target detection. In the context of evidence accumulation models, these results can be interpreted by changes in the accumulation parameters. The probabilistic description of perceptual awareness provides a benchmark for the choice of target and masker parameters in order to examine the underlying cognitive and neural dynamics of perceptual awareness.

## 1 Introduction

In the auditory modality, as opposed to the visual modality, relevant signals and noises in the environment are summed into an acoustic mixture. The auditory system thus has to face the problem of extracting relevant signals from a noisy acoustic environment, a process termed the “Cocktail Party Problem” [1, 2]. In this context, the auditory processes of scene analysis, e.g., auditory stream segregation or “auditory streaming”, are involved in the organization of acoustic inputs from multiple sound sources into coherent auditory objects [3]. Although the segregation of a complex auditory scene into coherent objects depends on weakly understood interactions between stimulus characteristics and task-driven processes [4], the simple simultaneity of independent sources makes the perception of a particular source more difficult [5]. This masking phenomenon occurs when the threshold of audibility of a given signal is raised by the presence of another sound.

Two types of masking have been described. First, “Energetic Masking”, or peripheral masking, appears when a target sound and a masking sound produce overlapping patterns of excitation at the cochlear and auditory nerve levels [6, 7]. Second, “Informational Masking” is “a degradation of auditory detection or discrimination of a signal embedded in a context of similar sounds; it is not related to energetic masking caused by physical interactions between signal and masker” [8]. Informational masking is thus the result of information processing at different stages beyond the auditory periphery [9] and it is related to perceptual grouping, source segregation, attention, memory and more general cognitive processes [5]. For some listeners, informational masking is robust to extensive training [10]. Nevertheless, informational masking may also disappear after extensive training and therefore does not necessarily reflect a loss of information in the peripheral auditory system but could rather denotes limitations imposed by central processing [11].

Informational masking has been studied using a classic paradigm consisting in presenting regularly repeated pure tone target among a multi-tone masker with random temporal and frequency characteristics [12, 13]. Stimulus properties, such as masker uncertainty and masker-target similarity, have been shown to have a strong influence on informational masking. The random temporal and frequency characteristics of the masker contribute to the masker uncertainty which can significantly reduce the detection of a target presented well above the auditory sensory threshold [11, 14–16].

Beyond the masker’s characteristics that influence informational masking, similarities shared by the masker and the target have a detrimental impact on target detection performance [17, 18]. High masker-target similarity promotes informational masking, whereas low masker-target similarity abolishes this masking [19]. The masker-target similarity can be related to different properties such as perceived location, direction of frequency glide, spectro-temporal coherence, or duration similarity [18].

In the informational masking paradigm, the target repetition rate and tone frequency are fixed for a given trial. Since the auditory system is highly efficient at processing regularities in acoustic sequences [20], it can gradually estimate the properties of each sound source on the basis of the acoustical information distributed over time [21]. As such, the target repetition rate plays a crucial role for perceptual grouping in a complex acoustic scene [22]. Prior studies of neural correlates of perceptual awareness have shown that neural activity increases at the frequency corresponding to the target repetition rate [4] with higher rates facilitating perceptual awareness [23, 24].

In summary, the relative contributions of various masker and target properties have been extensively studied in an informational masking paradigm where the main task of the participants is to detect a stream of target tones repeated regularly over time and embedded in a random-frequency multi-tone mask. Nevertheless, most informational masking studies, dealing with the effect of masker and target properties, measure discriminability or other static indices which neglect the temporal characteristics of information integration leading to the progressive build-up of a conscious percept [25]. Consequently, the effects of masker and target properties on the dynamics of the perceptual awareness remain relatively unsolved and misunderstood.

The studies of the progressive build-up of the conscious percept require information about the time course of the likelihood of perceptual awareness. Studies of perceptual awareness have examined the probability of detection as a discrete function of the number of target tone repetitions [25–27]. We propose here to use a statistical framework adapted to the modelling of continuous temporal processes leading to the change of a system’s state. In fact, the detection of the target can be considered as a transition from a “non percept” state to a “percept” state. In clinical and biostatistical medicine, the analysis of the time occurrence of such binary output has been performed using methods named “survival analysis” due to the considered states which are “alive” or “dead”. These analyses take into account the time course of the probability of state transition and provide alternative statistical methods adapted to any time-to-event outcome [28, 29]. To our knowledge, no study has yet investigated the time course of perceptual awareness using survival models to characterize its dynamics.

The main purpose of this study was to investigate how masker uncertainty, masker-target temporal similarity and target repetition rate can influence the dynamics of perceptual awareness of a target embedded in a multi-tone masker. To ensure this goal, we designed three experiments (simply denoted I, II and III) which sample different masker and target properties according to values used in previous studies [4, 24, 25, 30, 31] to provide a relatively complete set of relevant experimental conditions. Masker uncertainty was manipulated using masker mean inter-tone interval and number of frequencies per octave and characterized by the entropy of the tones’ distribution. Masker-target similarity was manipulated using masker and target tone durations and characterized as a duration difference. Last, target repetition rate was also varied due to its effect on auditory streaming and its putative effect on the dynamics of perceptual awareness. For these three experiments, in addition to the study of detection performance, we used survival model analysis to study the dynamics of perceptual awareness.

## 2 Method

This study has been approved by ethical comity under the reference: IRB00011835–2020-06-09–253.

### 2.1 Subjects

All subjects were recruited at Aix-Marseille University on the Saint-Charles campus. A new group of subjects was recruited for each experiment. Fourteen subjects (7 women) participated in Experiment I. Three subjects were left-handed (S1, S9 and S11). Ages were from 18 to 32 years with a mean of 24 years and SD of 3 years. Fourteen subjects (5 women) participated in Experiment II. Two subjects were left-handed (S2 and S9). Ages were from 18 to 38 years with a mean of 24 years and a SD of 5 years. Finally, thirteen subjects (6 women) participated in Experiment III. Two subjects were left-handed (S3 and S7). Ages were from 18 to 38 years with a mean of 24 years and a SD of 5 years. All subjects had reported normal hearing and no history of hearing or neurological impairment. Participation in the experiments was unpaid and on a volunteer basis following a call for participation. Subjects were informed of the study’s purpose and each volunteer gave written consent for participation.

### 2.2 Stimuli and apparatus

**Fig 1:**
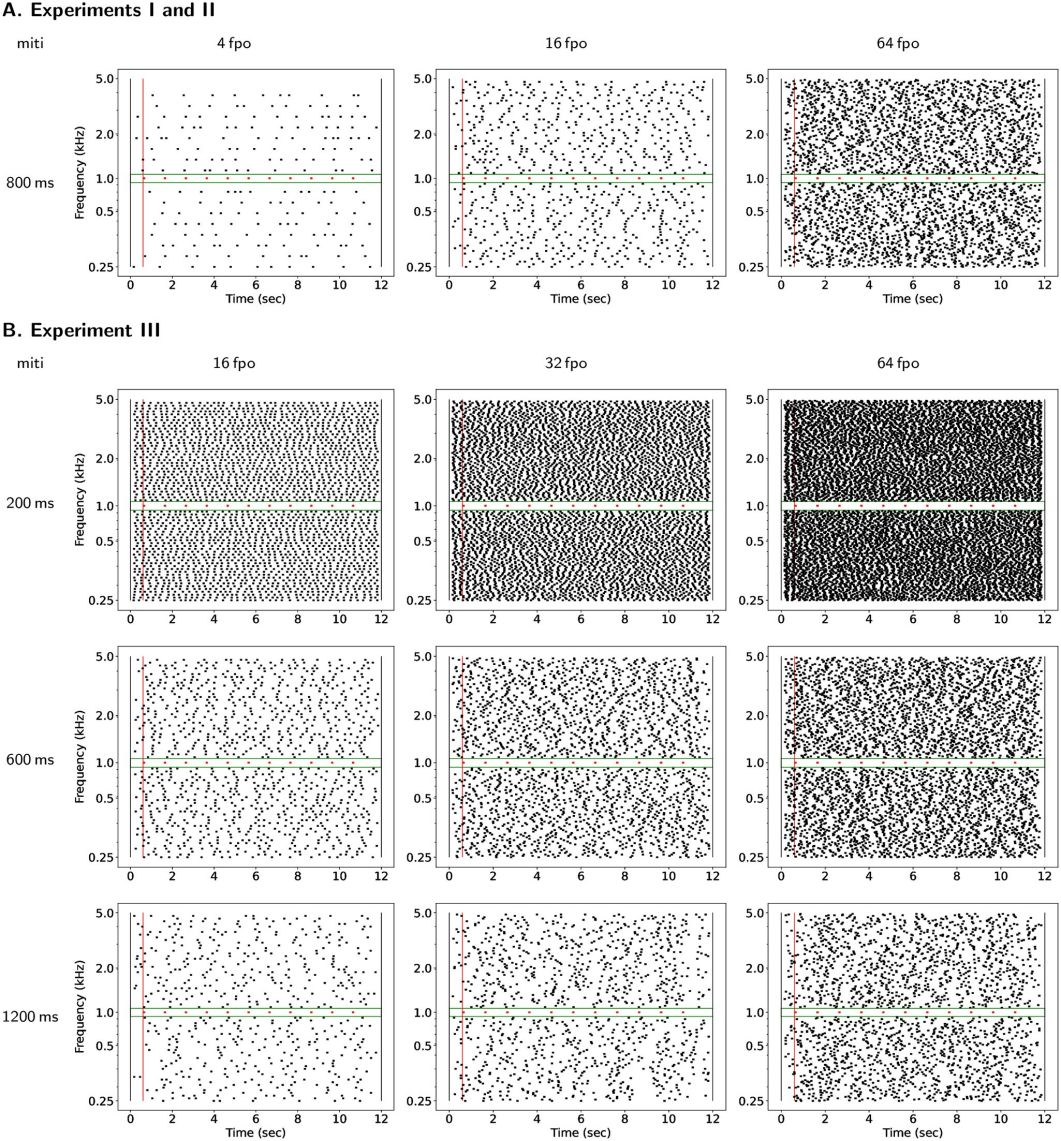
Graphical representations of a sample of the auditory stimuli used in the experiments. A target stream (in red) is presented in the protected region (in green) of a multi-tone masker. In the present examples, the target tones have a 1 kHz frequency, a duration of 100 ms and a repetition rate of 1 Hz. The masker tones (in black) span from 239 to 5 000 Hz and have a duration of 100 ms. Mean inter-tone interval (miti) are given on the rows and frequencies per octave (fpo) are given on the columns. (A) Experiments I and II and (B) Experiment III.

All auditory stimuli (Fig 1) were composed of a multi-tone masker and, in 67% of the trials, of a target [14, 25, 27, 32]. Auditory stimuli were all constructed using a common design based on a set of acoustical parameters displayed in Table 1 [4, 24, 25, 30, 31]. Target tones were presented at the same level as individual masker tones *i.e*. a target-to-masker level ratio of 0 dB [27]. The masker and target tones both had 10 ms on and off cosine-shaped ramps. When present, the regularly repeating target tone always started 600 ms after the beginning of the masker.

**Table 1:** Parameters of the experimental settings for the three experiments.

		Experiment I	Experiment II	Experiment III
Masker	Tone Duration (ms)	20, 60, 100	20, 60, 100	20
	Tone frequencies per octave	4, 16, 64	4, 16, 64	16, 32, 64
	Mean inter-tone interval (ms)	800	800	200, 600, 1200
Target	Tone Duration (ms)	20, 60, 100	20	60
	Rate (Hz)	1	5, 10, 20	1, 2, 5

Masker frequencies varied from 239 to 5000 Hz and target tone frequencies were taken from a set of six frequencies log-spaced between 489 and 2924 Hz.

Targets were composed of a regular series of tones defined by the tone duration and the tone repetition rate *i.e*. tones per second based on tone onset. To prevent subjects from selectively paying attention to a specific frequency range, the target tone frequencies changed randomly from trial to trial. Target tone frequency was drawn at random from a set of six equiprobable log spaced frequencies (489, 699, 1000, 1430, 2045 and 2924 Hz) [25, 27].

The maskers were characterized by duration, number of frequencies per octave (fpo) and the mean inter-tone intervals (miti). For the three experiments, the intervals between masking tones were drawn at random according to a uniform distribution with a constant minimum (100 ms) and a different maximum leading to different scale parameters. The tone frequencies in the masker were equally spaced on a logarithmic scale between 239 and 5 000 Hz [25, 27].

In order to ensure minimal energetic masking, a protected region surrounding the target was kept tone free in the masker. For each target, an equivalent rectangular bandwidth (ERB) [33, 34] was calculated using: *ERB* = 24.7(4.37*F*_*t*_ + 1) with *F*_*t*_ the frequency of the target in kHz. The protected region was centered on *F*_*t*_ and had a total extension of two ERB, *i.e*. one on each side of *F*_*t*_ (see Fig 1).

The masker-target temporal similarity was defined as the value of the difference between masker and target tone durations. Negative values denote a target tone duration longer than the masker tone duration and vice versa. The masker uncertainty was quantified using the entropy of the tones’ distribution. It characterizes both temporal and frequency uncertainty according to mean inter-tone interval and number of tone frequencies per octave respectively. Since the masker inter-tone intervals were drawn from an uniform distribution with a range Δ (in ms) between min. and max. inter-tone intervals, entropy *H* of this uniform distribution is: *H*(*x*) = log(Δ). Considering each frequency per octave as an independent process, the entropy of the whole masker *M* for *n* frequencies per octave is: *H*(*M*) = *n*log(Δ). The values of entropy for all the combinations of the masker parameters are given in Table 2.

**Table 2:** Masker uncertainty quantified by entropy (in nats) of the tone distribution.

		Exp. I—II	Exp. III		
miti (ms)		800	200	600	1200
Δ (ms)		1400	200	1000	2200
fpo	4	28.97	—	—	—
	16	—	84.77	110.52	123.13
	32	231.82	169.55	221.05	246.28
	64	463.63	339.09	442.09	492.56

miti: mean inter-tone interval, fpo: frequencies per octave, Δ: range of the inter-tone intervals (max.—min.).

Auditory stimuli were generated using the Python programming language (version 3.5.2). They were digitized with a sampling rate of 44100 Hz and 16 audio bit depth. All stimuli were delivered with an acoustic intensity level calibrated to 70 dB SPL.

### 2.3 Procedures and conditions

Each experiment was composed of 243 trials, which were randomly distributed into 6 blocks of 41 or 40 trials. Each trial lasted 12 seconds and trials were separated by 4 seconds of silence. The total duration of the experiment was 65 minutes. The task of the participants was to press the space bar on a computer keyboard as soon as they detected the target. Subjects were instructed to answer as accurately and as quickly as possible as soon as they were certain of the target presence. The subjects did not receive any feedback on the validity or timeliness of their answers and they were not given the opportunity to change their answer in the event that they realised they had made a mistake (false alarm). There was no systematic recording of such a situation and it is therefore not possible to measure its extent. Subjects were informed that the target would not necessarily be present in each trial. No information regarding target probability was given. Participants had a training session, composed of trials with and without target which continued until the subject correctly detected 10 trials with targets. The trials used in the training block were composed of maskers with tone duration of 20 or 60 ms and mean inter-tone interval of 600 or 800 ms. Targets were composed of tones with a frequency of 1 kHz and duration of 100 ms. Target repetition rate varied randomly between trials and was either 1 or 2 Hz.

Auditory stimuli were produced by a DELL PRECISION M4800 computer (i7 4900 MQ processor, 16GB DDR3 RAM, NVidia Quadro K2100M running Windows 7 with an Intel Lynx Point PCH sound card) and presented diotically to participants with a calibrated circumaural headset (Sennheiser HDA 600) in a soundproof room. The stimuli were presented to participants using the Eprime software (version 2.0, Psychology Software Tools).

### 2.4 Experiments

In each experiment, the probability of trials without a target was 33% leading to 81 trials without a target and 162 trials with a target for a total of 243 trials per experiment. Each experimental combination of masker and target properties was presented once per target tone frequency (6) to each subject. In the three experiments, masker tone frequencies per octave varied according to three levels: 4, 16 and 64 frequencies per octave for Experiments I and II and 16, 32, 64 frequencies per octave for Experiment III (see Table 1).

In Experiment I, the masker and target tone durations were manipulated according to three levels: 20, 60 and 100 ms. The target repetition rate was equal to 1 Hz. The inter-tone intervals of the masker were drawn from an uniform distribution (min.: 100 ms, max.: 1500 ms) leading to a mean inter-tone interval of 800 ms. Masker-target similarity thus varied from -80 to 80 ms and the values of the masker uncertainty are given in Table 2.

In Experiment II, the target repetition rate varied according to three levels: 5, 10 and 20 Hz. The target tone duration was set to 20 ms in order to ensure that successive tones do not overlap in the high repetition rate condition. Masker tone durations were adjusted to the same values as in Experiment I (20, 60 or 100 ms). The masker inter-tone intervals were drawn from the same uniform distribution as in Experiment I leading to the same mean inter-tone interval of 800 ms and masker uncertainty (Table 2). Masker-target similarity thus varied from 0 to 80 ms.

In Experiment III, the target tone duration was set to 60 ms and masker tone duration was set to 20 ms, leading to a single value of masker-target similarity (40 ms). Target repetition rate varied according to three levels: 1, 2 and 5 Hz. Masker inter-tone intervals were drawn from uniform distributions with a constant minimum (100 ms) and variable maxima (300, 1100 or 2300 ms), yielding three values of mean inter-tone interval: 200, 600 and 1200 ms leading to an increase in masker uncertainty with the duration of the mean inter-tone interval. The values of the masker uncertainty are given in Table 2.

### 2.5 Analysis

Trial detection times were recorded each time the participant first pressed the space bar. For each experiment, data were read from the EPrime raw file using Python scripts [35] and statistics were performed with the R software [36, 37].

Each trial was categorized as either a hit, a miss, a false alarm or a correct rejection according to both the target’s presence and the participant’s response. Since detecting a regularity requires hearing at least two repetitions of the target tone, any detection occurring faster was considered as a guess and dismissed from valid responses. For each experiment, the time cut-off was adapted to the fastest target tone repetition rate leading to a cut-off of 1600 ms for Experiment I, 700 ms for Experiment II and 1100 ms for Experiment III.

The detection performance index (*d*′) was computed from the hit rate (HR) and false alarm rate (FAR) after a z-score transformation with the percent point function [38, 39]: *d*′ = *z*(HR) − *z*(FAR). This index was computed for each subject and in each experimental condition where FAR can be defined. Since a false alarm corresponds to a trial where the target is absent, FAR cannot be defined for experimental conditions characterized by target properties and thus *d*′ can only be computed for the experimental conditions characterized by masker properties (i.e. uncertainty).

Complete detailed data analysis are provided for Experiment I, II and III in S1–S3 Files respectively. They first consist in a qualitative exploration of the distributions of HR, FAR and *d*′ and of the distribution of reaction time for hit trials for each subject in order to discard obvious outliers.

Then, detection performance data were analyzed using mixed-effect models with the nlme R library [40]. Mixed-effect models were estimated using the performance index (*d*′) as the response variable, the masker uncertainty as a fixed effect and the subject id. as a random effect for the intercept parameter.

The previous analysis allows us to characterise the “detection performance” on the basis of the *d*′ index computed using the four categories of trials (hit, miss, false alarm and correct rejection). In a second analysis, only trials where a target is present are taken into account. These trials are characterized by their detection time which can be (right-)censored in the case of “miss”. These data correspond to time-to-event data associated with the transition between two binary states: from “non-perception” to “perception” of the target. Such data are analysed using survival models which allow one to characterize the effects of experimental parameters on the dynamics of such transition. In the present case, it is considered that survival models allow one to characterise the influence of masker and target properties on the dynamics of “perceptual awareness” build-up. We provide a quick summary of the relevant concepts of survival analysis needed for the present study. Review articles [29, 41] or books [42, 43] give more in-depth descriptions of the techniques.

Statistical analysis of the effect of predictors on the time-to-event variable are usually performed using the Cox proportional hazards (PH) model [44, 45]. This model is based on the hazard rate function *h*(*t*) which describes the instantaneous rate of occurrence of the event (here the target detection) over time. Thus, the hazard rate *h*(*t*) is the instantaneous risk of detecting the target at a time *t* for those who have not yet detected it until *t* and can be formally defined as:

$$\begin{array}{c} h\left(t\right)=\underset{\Delta t\to 0}{\text{lim}}\frac{\text{Pr}(t\leq T<t+\Delta t\;|\;T\geq t)}{\Delta t} \end{array}$$ (1)

Cox PH model assumes that the predictors have a multiplicative effect on the hazard and that this effect is constant over time, i.e.,

$$\begin{array}{c} h\left(t|x\right)=h_{0}\left(t\right)e^{\beta_{1}x_{1}+\cdots +\beta_{p}x_{p}} \end{array}$$ (2)

where *h*(*t*|*x*) is the hazard at time *t* for a subject with a set of *p* predictors *x*_1_, ⋯, *x*_*p*_, *h*_0_(*t*) is the baseline hazard function and *β*_1_, ⋯, *β*_*p*_ are the model parameters describing the effect of the predictors on the overall hazard.

Since repetitions were performed over participants, the influence of experimental conditions on reaction times was analyzed with a repeated measurements techniques [46]. In this case, mixed-effect models are highly recommended [28, 47, 48]. In survival analysis, mixed-effect models correspond to frailty models which extend the Cox PH model by adding a subject-specific random effect with a multiplicative effect on hazard [49]. This incorporation of subject-specific random effects in the Cox PH model modifies the baseline hazard function *h*_0_(*t*) meaning that relative effect of a given covariate pattern on the baseline hazard function varies across subjects. The hazard at time *t* for a subject with a fragility *ξ* is thus given as:

$$\begin{array}{c} h\left(t|x,\xi \right)=h_{0}\left(t\right)e^{\beta_{1}x_{1}+\cdots +\beta_{p}x_{p}+\xi } \end{array}$$ (3)

The detection time obtained in the three experiments were analysed using frailty models implemented in the survival R packages [50]. Masker uncertainty, masker-target temporal similarity and target repetition rate represent the fixed-effects whereas the frailty terms with subject id. correspond to random effects of the fitted models.

For each fitted model, S1–S3 Files provide diagnostic plots for residuals and influential data. Cox-Snell residuals [28, 51, 52] are used in the case of Cox PH models. After deleting any influential outliers with a flawed behavior e.g. with a high FAR, analysis of variance were performed in order to assess the global statistical significance of the effects of experimental parameters and their interactions.

In the case of global statistical effect, the complete interactions between experimental parameters were studied on the basis of all pairwise comparisons using estimated marginal means implemented in the emmeans R package. Estimated marginal means correspond to the values of the model parameters averaged for the adequate combination of factor modalities. The supplementary files give the complete results which are summed up in the article in tables which group together experimental conditions using compact letter display (CLD) [53]. Results are illustrated by hazard rate curves estimated according to the grouped conditions given in the tables.

The characterisation of the dynamics of perceptual awareness is also illustrated using the cumulative distribution function *F*(*t*) which is the probability that the detection time is lower than *t* and defined as:

$$\begin{array}{c} F(t)=\text{Pr}(T\leq t) \end{array}$$ (4)

This function varies from 0 to 1 and is thus easier to interpret than the hazard rate function used in the Cox PH model.

## 3 Results

The observation of detection performance for each experimental block showed that the performance index in the first block was lower than in the other blocks (see S1 Fig). The data from the first block was thus discarded for each experiment to prevent learning variability. The behavioural results of performance and detection times are reported in Table 3.

**Table 3:** Behavioral results for detection time and performance in the three experiments.

	Experiment I	Experiment II	Experiment III
Mean Detection Time (ms)	5430 ± 2804	3019 ± 2051	3104 ± 2070
Hit’s Rate (%)	0.46 ± 0.19	0.78 ± 0.09	0.88 ± 0.08
False Alarm’s Rate (%)	0.25 ± 0.19	0.13 ± 0.11	0.08 ± 0.05
d’	0.71 ± 0.32	2.11 ± 0.27	2.73 ± 0.55

Results are given as mean ± standard deviation.

**Fig 2:**
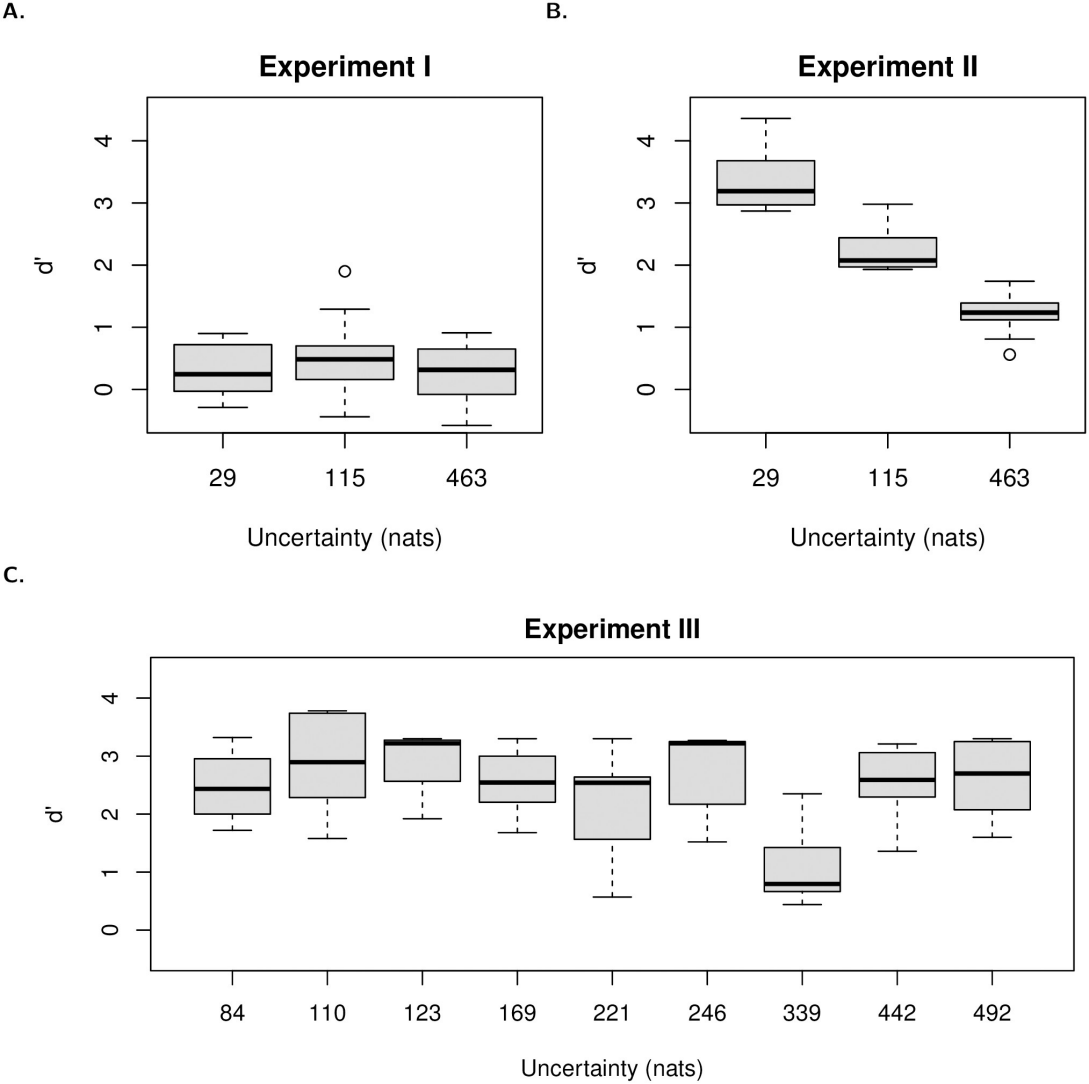
Detection performance index *d*′ as a function of masker uncertainty in Experiment I, II and III. Masker uncertainty is measured by entropy in nats.

**Fig 3:**
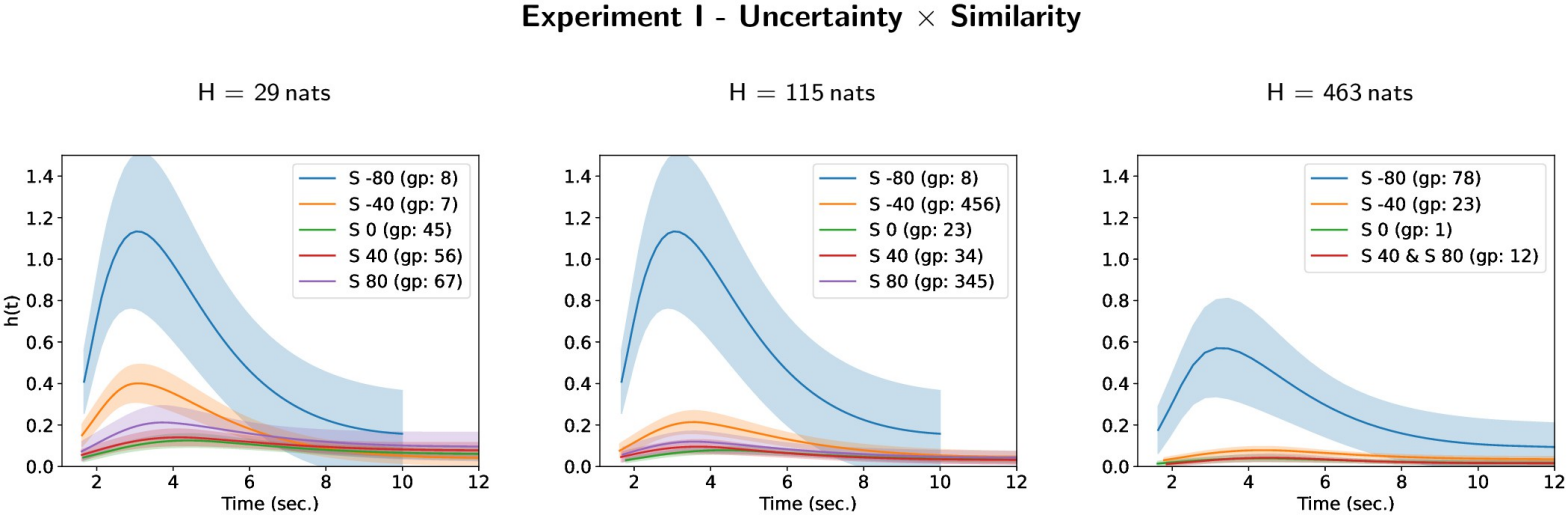
Hazard rate functions *h*(*t*) for masker uncertainty and masker-target similarity in Experiment I. Each figure depicts the estimates of the hazard rate functions for each different group of the compact letter display for experimental conditions defined by the interaction between masker uncertainty and masker-target similarity.

The detection performance index (*d*′) is depicted as a function of the masker uncertainty for Experiment I, II and III in Fig 2. The instantaneous rate of target detection is represented by the estimated hazard rate functions given for each group of experimental conditions for Experiment I, II and III on Figs 3, 5 and 7 respectively.

### 3.1 Experiment I

The complete data analysis for Experiment I is given in S1 File. No subject was discarded on the basis of the qualitative inspection of the distributions of the performance indices and the reaction times (*n* = 14).

No significant effect of the masker uncertainty on the detection performance index (*d*′) was observed (*F*(2, 26) = 1.16, *p* = 0.33, see Fig 2).

The analysis of the Cox PH model with frailty term showed no significant effect of the frailty term (*χ*^2^ = 0.27, *df* = 1, *p* = 0.61) whereas the masker-target temporal similarity (*χ*^2^ = 286.461, *df* = 4, *p* < 0.001), the masker uncertainty (*χ*^2^ = 167.07, *df* = 2, *p* < 0.001) and their interaction (*χ*^2^ = 430.98, *df* = 20.5, *p* < 0.001) have a significant effect on the hazard rate of target perceptual awareness.

The results of all-pairwise comparisons for the interaction between masker uncertainty and masker-target temporal similarity are given in S1 File and their CLD summary is given in Table 4. An illustration of the corresponding hazard rate curves for each level of masker-target similarity in each case of masker uncertainty is given on Fig 3 (and the converse illustration is given on S2 Fig). The highest hazard rate of target perceptual awareness is observed when the target tone duration is 80 ms longer than the masker tone duration (masker-target similarity: *S* = −80 ms) for the two lowest masker uncertainty (*H* = 29 nats and *H* = 115 nats, i.e. CLD group 8). This hazard rate decreases for the highest masker uncertainty (*H* = 463 nats). The hazard rate curves observed when the target tone duration is equal or shorter than the masker tone duration (*S* = 0 ms, *S* = 40 ms and *S* = 80 ms) can hardly be differentiated for each masker uncertainty level. The hazard rate curves observed in the case where the target tone duration is 40 ms longer than the masker tone duration (*S* = −40 ms) have an intermediate behavior which can be differentiated from the conditions where the target tone duration is equal or shorter than the masker tone duration (*S* = 0 ms, *S* = 40 ms and *S* = 80 ms) for the lowest uncertainty (*H* = 29 nats) and tends to the curves observed in these conditions as uncertainty increases.

**Table 4:** Estimated marginal means and compact letter display for all-pairwise comparisons of the interaction between masker-target similarity and masker uncertainty in Experiment I.

Uncertainty	Similarity	emmean	SE	df	asymp.LCL	asymp.UCL	.group
463	80	-3.5107	0.3383	Inf	-4.1738	-2.8476	12
463	0	-3.2871	0.2497	Inf	-3.7764	-2.7978	1
463	40	-3.2672	0.2709	Inf	-3.7981	-2.7363	12
115	0	-2.6058	0.2337	Inf	-3.0637	-2.1478	23
463	-40	-2.5652	0.2382	Inf	-3.0320	-2.0984	23
115	40	-2.3467	0.2410	Inf	-2.8191	-1.8743	34
115	80	-2.0614	0.2578	Inf	-2.5666	-1.5562	345
29	0	-2.0002	0.2250	Inf	-2.4412	-1.5591	45
115	-40	-1.8532	0.2310	Inf	-2.3061	-1.4004	456
29	40	-1.7631	0.2284	Inf	-2.2108	-1.3153	56
29	80	-1.3134	0.2455	Inf	-1.7946	-0.8322	67
29	-40	-1.0384	0.2248	Inf	-1.4790	-0.5978	7
463	-80	-0.8056	0.2628	Inf	-1.3207	-0.2906	78
115	-80	-0.2971	0.2686	Inf	-0.8236	0.2294	8
29	-80	0.0000	0.0000	Inf	0.0000	0.0000	8

Results are given on the log (not the response) scale. Confidence level used: 0.95. P-value adjustment: Tukey method for comparing a family of 15 estimates. Significance level used: *α* = 0.05 Uncertainty: masker uncertainty, Similarity: masker-target similarity, emmean: estimated marginal mean, SE: standard error, df: degrees of freedom, asymp.LCL: asymptotic lower contrast limit, asymp.UCL: asymptotic upper contrast limit, .group: compact letter group.

**Fig 4:**
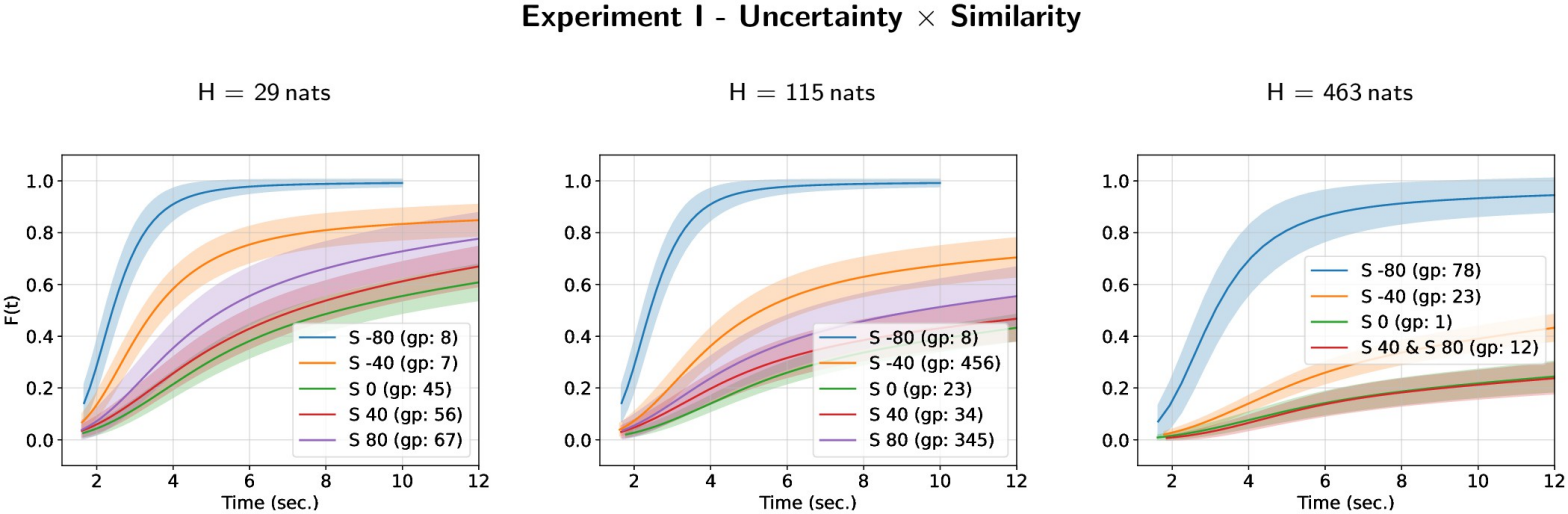
Cumulative distribution functions *F*(*t*) for masker uncertainty and masker-target similarity in Experiment I. Each figure depicts the estimates of the cumulative distribution functions for each different group of the compact letter display for experimental conditions defined by the interaction between masker uncertainty and masker-target similarity.

The cumulative distribution curves for each level of masker-target similarity in each case of masker uncertainty are given on Fig 4 (and the converse illustration is given on S3 Fig). They show that, with the exception of the case where the target tone duration is 80 ms longer than the masker tone duration (masker-target similarity: *S* = −80 ms), the dynamics of the perceptual awareness is considerably slowed down by the increase in the masker uncertainty. Indeed, in most cases the probability that the perception has taken place before the end of the trial (12 sec.) is less than 0.8 or even less than 0.5 for the highest masker uncertainty (*H* = 463 nats). One can also observe (mainly on S3 Fig) that increasing the difference in duration between the masker and the target tones increases the speed of perception. But this increase is faster when the target tone duration is longer than the masker tone duration.

### 3.2 Experiment II

The complete data analysis for Experiment II is given in S2 File. A subject (S6) was discarded from the analyses on the basis of the atypical distribution of her reaction times. Subsequent analysis of the mixed-effect linear model for the performance index (*d*′) and of the Cox PH model for the detection times lead to the suppression of three more subjects (S1, S2, S8) as influential outliers with FAR clearly higher than that of the other subjects. The analyses of Experiment II were thus limited to *n* = 10 subjects.

The analysis of variance of mixed-effect linear model showed a significant effect of masker uncertainty on the performance index (*d*′) (*F*(2, 27) = 74.2, *p* < .001). The analysis of all-pairwise comparisons showed a significant difference between all pairs of conditions which reflects a significant decrease of detection performance with the increase of masker uncertainty (see Fig 2).

The analysis of the Cox PH model with frailty term showed significant effects for the frailty term (*χ*^2^ = 13.5, *df* = 1, *p* < 0.001), masker uncertainty (*χ*^2^ = 428.5, *df* = 2, *p* < 0.001), masker-target similarity (*χ*^2^ = 170.2, *df* = 2, *p* < 0.001) and target repetition rate (*χ*^2^ = 249.1, *df* = 2, *p* < 0.001). All the interactions had also a significant effect on the hazard rate of target detection (masker uncertainty × masker-target similarity: *χ*^2^ = 13, *df* = 4, *p* = 0.011; masker uncertainty × target repetition rate: *χ*^2^ = 12.1, *df* = 4, *p* = 0.017; masker-target similarity × target repetition rate: *χ*^2^ = 19.8, *df* = 4, *p* < 0.001; and the triple interaction: *χ*^2^ = 178.7, *df* = 16.4, *p* < 0.001).

**Fig 5:**
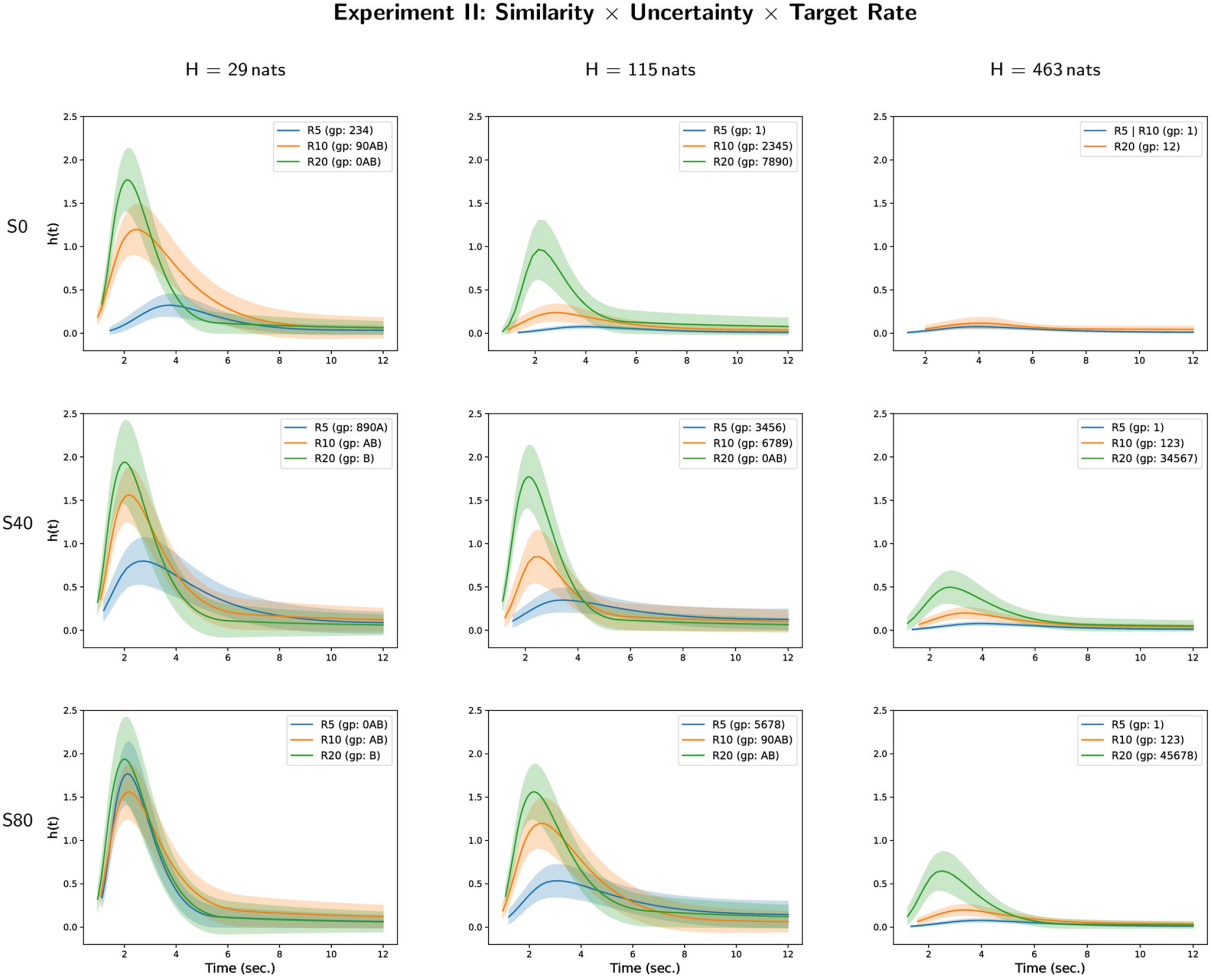
Hazard rate functions *h*(*t*) for masker uncertainty, masker-target similarity and target repetition rate in Experiment II. Each figure depicts the estimates of the hazard rate functions for each different group of the compact letter display for experimental conditions defined by the interaction between masker uncertainty, masker-target similarity and target repetition rate.

The results of all-pairwise comparisons for the triple interaction are given in S2 File and their CLD summary is given in Table 5. An illustration of the corresponding hazard rate curves for each level of target repetition rate in each condition defined by the interaction between masker-target temporal similarity and masker uncertainty is given on Fig 5 (and the two other possible representations are given on S4 and S5 Figs). One can observe a decrease of hazard rate of target perceptual awareness with the increase of masker uncertainty and with the decrease of target repetition rate, except in the case of the lowest masker uncertainty (*H* = 29 nats) and of highest difference between target and masker tone durations (*S* = 80 ms). In this case, the hazard rate of target perceptual awareness is higher for target repetition rate of 5 Hz than for target repetition rate of 10 Hz. As a general principle, the lowest hazard rate of target perceptual awareness is observed when target tone duration is equal to masker tone duration (*S* = 0 ms) but the effect of masker-target temporal similarity is highly dependent of the interaction of the two other parameters (see S5 Fig).

**Table 5:** Estimated marginal means and compact letter display for all-pairwise comparisons of the interaction between masker-target similarity and masker uncertainty in Experiment II.

Similarity	T.Rate	Uncertainty	emmean	SE	df	asymp.LCL	asymp.UCL	.group
0	5	463	-1.3599	0.3220	Inf	-1.9910	-0.7289	1
0	10	463	-1.2571	0.2986	Inf	-1.8425	-0.6718	1
0	5	115	-1.1938	0.2925	Inf	-1.7670	-0.6206	1
80	5	463	-1.0947	0.2818	Inf	-1.6471	-0.5424	1
40	5	463	-1.0778	0.2770	Inf	-1.6208	-0.5348	1
0	20	463	-0.6114	0.2551	Inf	-1.1114	-0.1114	12
40	10	463	-0.3128	0.2425	Inf	-0.7881	0.1625	123
80	10	463	-0.2938	0.2403	Inf	-0.7648	0.1772	123
0	5	29	0.0000	0.0000	Inf	0.0000	0.0000	234
0	10	115	0.0116	0.2313	Inf	-0.4417	0.4649	2345
40	5	115	0.3892	0.2253	Inf	-0.0524	0.8309	3456
40	20	463	0.4186	0.2255	Inf	-0.0233	0.8605	34567
80	20	463	0.6976	0.2236	Inf	0.2594	1.1357	45678
80	5	115	0.8100	0.2164	Inf	0.3859	1.2341	5678
40	10	115	1.0322	0.2200	Inf	0.6009	1.4635	6789
0	20	115	1.1872	0.2205	Inf	0.7551	1.6193	7890
40	5	29	1.2853	0.2185	Inf	0.8570	1.7135	890A
0	10	29	1.5819	0.2193	Inf	1.1520	2.0118	90AB
80	10	115	1.7138	0.2206	Inf	1.2814	2.1462	90AB
80	5	29	1.8252	0.2248	Inf	1.3847	2.2657	0AB
0	20	29	1.8364	0.2202	Inf	1.4048	2.2680	0AB
40	20	115	1.9370	0.2181	Inf	1.5095	2.3645	0AB
80	10	29	1.9539	0.2202	Inf	1.5224	2.3855	AB
80	20	115	1.9883	0.2196	Inf	1.5579	2.4186	AB
40	10	29	1.9949	0.2204	Inf	1.5629	2.4269	AB
40	20	29	2.2040	0.2205	Inf	1.7718	2.6361	B
80	20	29	2.2100	0.2203	Inf	1.7783	2.6418	B

Results are given on the log (not the response) scale. Confidence level used: 0.95. P-value adjustment: Tukey method for comparing a family of 27 estimates. Significance level used: *α* = 0.05 Uncertainty: masker uncertainty, Similarity: masker-target similarity, T.Rate: target repetition rate. emmean: estimated marginal mean, SE: standard error, df: degrees of freedom, asymp.LCL: asymptotic lower contrast limit, asymp.UCL: asymptotic upper contrast limit, .group: compact letter group.

**Fig 6:**
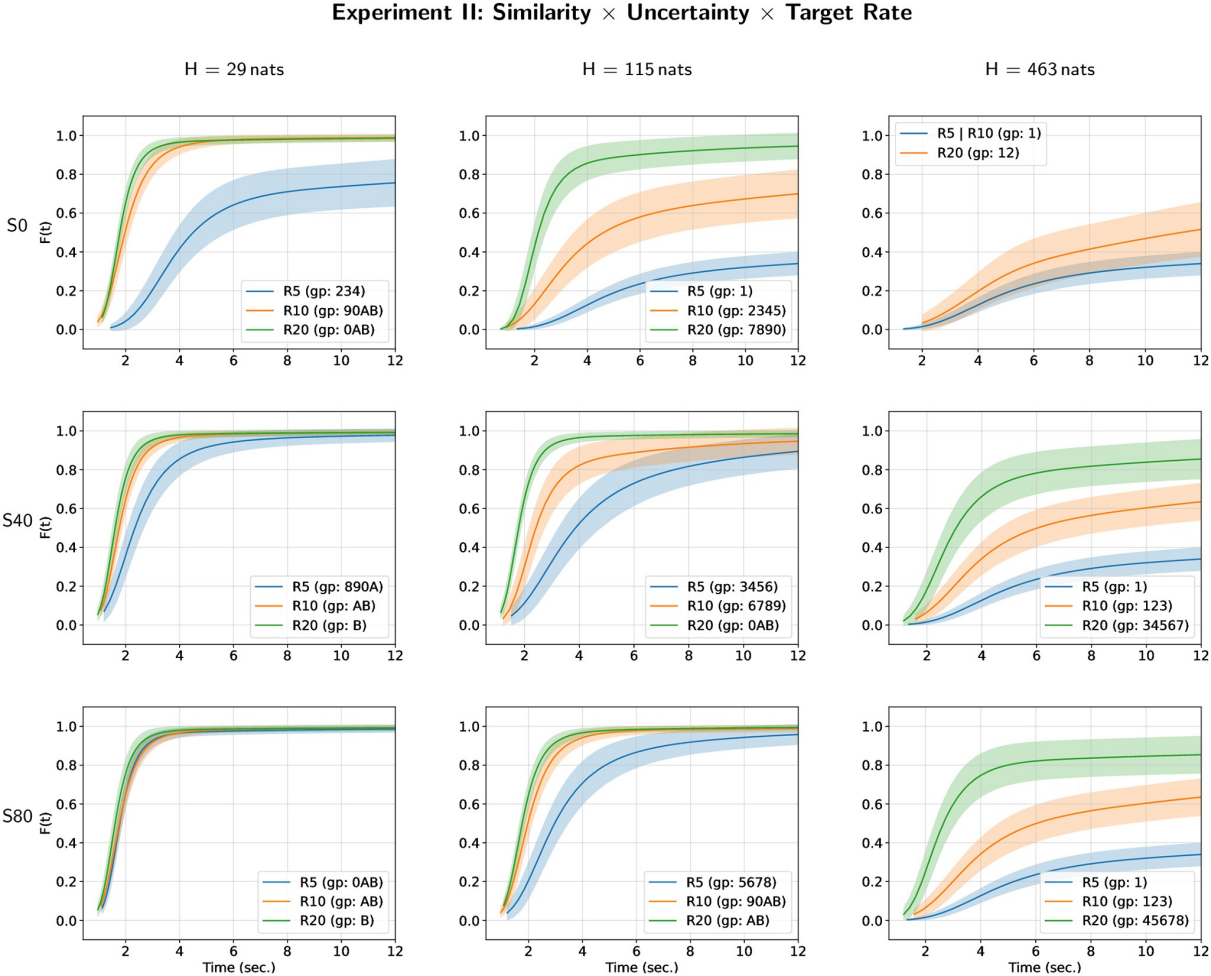
Cumulative distributions functions *F*(*t*) for masker uncertainty, masker-target similarity and target repetition rate in Experiment II. Each figure depicts the estimates of the cumulative distribution functions for each different group of the compact letter display for experimental conditions defined by the interaction between masker uncertainty, masker-target similarity and target repetition rate.

The cumulative distribution curves for each level of target repetition rate in each condition defined by the interaction between masker-target temporal similarity and masker uncertainty are given on Fig 6 (and the two other possible representations are given on S6 and S7 Figs). These curves complement the observations made with the hazard rate functions. They show that the increase in masker uncertainty and the decrease in the target repetition rate decrease or prevent perceptual awareness of the target. In the case of low masker-target temporal similarity, only experimental conditions with high target repetition rate and low masker uncertainty allow a high probability of perceptual awareness to be achieved over time corresponding to a trial.

### 3.3 Experiment III

The complete data analysis for Experiment III is given in S3 File. The qualitative inspection of the distributions of the detection performance index (*d*′) shows that one subject (S8) has a high FAR. This subject was thus discarded from subsequent analysis (*n* = 12).

A significant effect was found for masker uncertainty on the detection performance index (*d*′) (*F*(8, 88) = 11.9, *p* < 0.001). All-pairwise comparisons show that the detection performance is significantly lower for the masker uncertainty condition where *H* = 339 nats (i.e., 64 fpo and miti: 200 ms) than for all the other uncertainty conditions.

The analysis of the frailty model showed no significant effect of the frailty term (*χ*^2^ = 1.25, *df* = 1, *p* = 0.26) whereas significant effects were observed for masker uncertainty (*χ*^2^ = 458.32, *df* = 8, *p* < 0.001), for the target repetition rate (*χ*^2^ = 153.81, *df* = 2, *p* < 0.001) and for their interaction (*χ*^2^ = 197.78, *df* = 26.1, *p* < 0.001).

**Fig 7:**
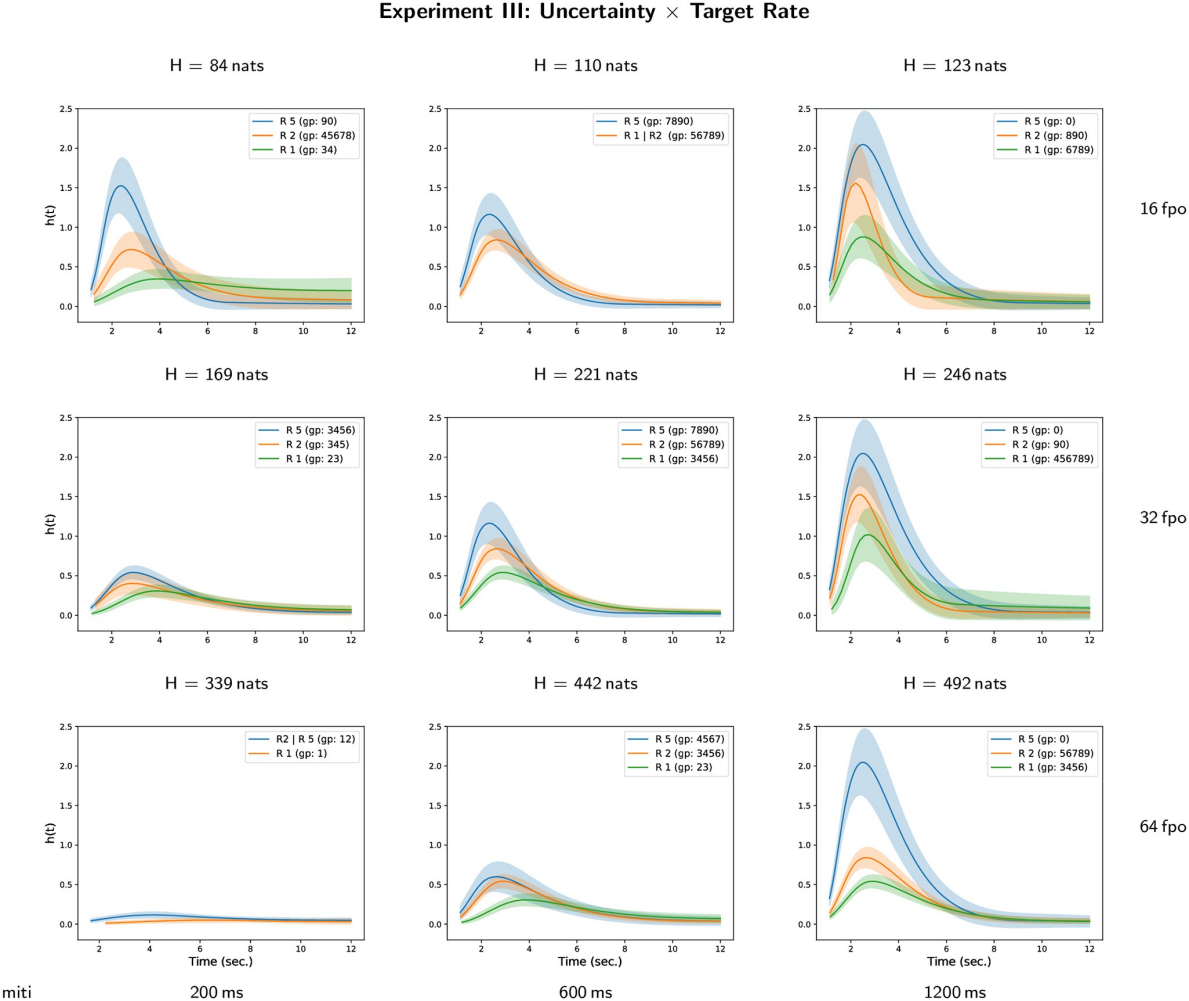
Hazard rate functions *h*(*t*) for masker uncertainty and target repetition rate in Experiment III. Each figure depicts the estimates of the hazard rate functions for each different group of the compact letter display for experimental conditions defined by the interaction between masker uncertainty and target repetition rate.

The results of all-pairwise comparisons for the interaction between masker uncertainty and target repetition rate are given in S3 File and their CLD summary is given in Table 6. An illustration of the corresponding hazard rate curves for each level of target repetition rate in each case of masker uncertainty is given on Fig 7 (and the converse representation is given on S8 Fig). The hazard rate of target perceptual awareness increases significantly with target repetition rate but there is no monotonous effect of the masker uncertainty on the hazard rate of target perceptual awareness. One can observe (Fig 7) that the two parameters defining the masker uncertainty, i.e. the number of frequencies per octave and the mean inter-tone interval, have underlying effects. The hazard rate of the target perceptual awareness increases with mean inter-tone interval and decreases with the number of frequencies per octave. Nevertheless, these overall effects of the masker parameters are modulated by the effect of target tone repetition rate.

**Table 6:** Estimated marginal means and compact letter display for all-pairwise comparisons of the interaction between masker-target similarity and masker uncertainty in Experiment III.

T.Rate	Uncertainty	emmean	SE	df	asymp.LCL	asymp.UCL	.group
1	339	-1.7181	0.2668	Inf	-2.2411	-1.1951	1
2	339	-1.0969	0.2231	Inf	-1.5343	-0.6596	12
5	339	-0.8521	0.2168	Inf	-1.2771	-0.4271	12
1	442	-0.3084	0.1990	Inf	-0.6984	0.0816	23
1	169	-0.2980	0.1928	Inf	-0.6759	0.0798	23
1	84	0.0000	0.0000	Inf	0.0000	0.0000	34
2	169	0.0723	0.1960	Inf	-0.3118	0.4564	345
1	492	0.1572	0.1885	Inf	-0.2122	0.5267	3456
1	221	0.2434	0.1895	Inf	-0.1280	0.6149	3456
2	442	0.2913	0.1974	Inf	-0.0956	0.6783	3456
5	169	0.3264	0.1903	Inf	-0.0466	0.6993	3456
5	442	0.4842	0.1943	Inf	0.1035	0.8649	4567
2	84	0.5693	0.1866	Inf	0.2037	0.9349	45678
1	246	0.7110	0.1927	Inf	0.3333	1.0888	456789
2	221	0.7120	0.1910	Inf	0.3377	1.0862	56789
1	110	0.7510	0.1878	Inf	0.3829	1.1191	56789
2	110	0.7557	0.1933	Inf	0.3769	1.1346	56789
2	492	0.7829	0.1912	Inf	0.4081	1.1577	56789
1	123	0.8308	0.1895	Inf	0.4595	1.2022	6789
5	221	1.0993	0.1891	Inf	0.7286	1.4700	7890
5	110	1.1616	0.2030	Inf	0.7637	1.5596	7890
2	123	1.2463	0.1920	Inf	0.8700	1.6226	890
2	246	1.3122	0.1927	Inf	0.9345	1.6899	90
5	84	1.3657	0.1915	Inf	0.9903	1.7411	90
5	246	1.5825	0.1931	Inf	1.2040	1.9610	0
5	492	1.7711	0.1907	Inf	1.3974	2.1449	0
5	123	1.7801	0.1931	Inf	1.4017	2.1586	0

Results are given on the log (not the response) scale. Confidence level used: 0.95. P-value adjustment: Tukey method for comparing a family of 27 estimates. Significance level used: *α* = 0.05. Uncertainty: masker uncertainty, T.Rate: target repetition rate, emmean: estimated marginal mean, SE: standard error, df: degrees of freedom, asymp.LCL: asymptotic lower contrast limit, asymp.UCL: asymptotic upper contrast limit, .group: compact letter group.

**Fig 8:**
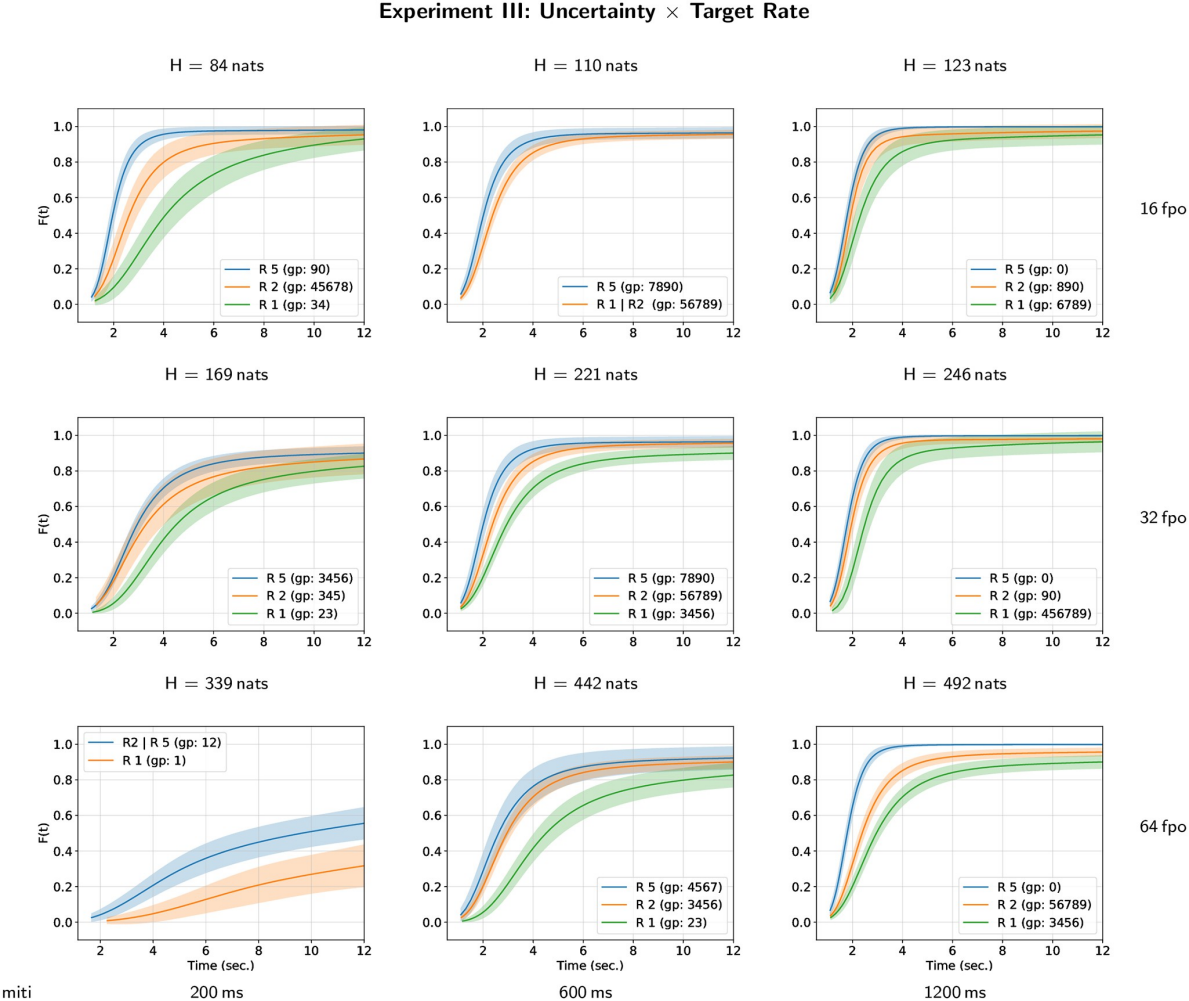
Cumulative distribution functions *F*(*t*) for masker uncertainty and target repetition rate in Experiment III. Each figure depicts the estimates of the cumulative distribution functions for each different group of the compact letter display for experimental conditions defined by the interaction between masker uncertainty and target repetition rate.

Cumulative distribution curves for each level of target repetition rate in each case of masker uncertainty are given on Fig 8 (and the converse representation is given on S9 Fig). In most of the experimental conditions, the probability of perceptual awareness at the end of the trial is higher than 0.8 with the exception of the condition with *H* = 339 nats where the probability is lower than 0.6. As observed in Experiment II, the highest target repetition rate (5 Hz) improve the dynamics of the perceptual awareness compared to the lower target repetiton rate. The time constant of the perceptual awareness dynamics decrease with inter-tone interval and with the number of frequencies per octave. Nevertheless, the dynamics of perceptual awareness is highly dependent on the combination of the experimental parameters.

### 3.4 Summary

Discrimination index (*d*′) allows one to study the effect of masker uncertainty, only, on the detection performance in the three experiments. While masker uncertainty has no effect in Experiment I, Experiment II shows a decrease in detection performance as masker uncertainty increases and Experiment III shows that a specific combination of frequencies per octave and mean inter-tone interval decrease detection performance.

Experiment I shows that perceptual awareness of the target decreases when masker uncertainty increases and when masker-target temporal similarity increases. More specifically, perceptual awareness of the target decreases for masker tone duration longer than target tone duration. Experiment II also shows globally that perceptual awareness of the target decreases when masker uncertainty increases and when target repetition rate decreases. Moreover, the effect of masker-target similarity depends on the values of the other parameters. Experiment III shows that perceptual awareness of the target decreases when target repetition rate decreases. In the case of masker uncertainty, the temporal and frequency uncertainty of the masker have opposite effects: the perceptual awareness of the target increases when temporal uncertainty increases and when frequency uncertainty decreases. In all the experiments, the interactions between experimental factors are significant leading to the conclusion that the effects of masker and target properties on the dynamics of the perceptual awareness are highly context dependent.

## 4 Discussion

This study investigated how masker-target temporal similarity (mostly Exp. I), target repetition rate (mostly Exp. II) and masker uncertainty (mostly Exp. III) affected the performance and the dynamics of perceptual awareness related to the detection of a regular target using an informational masking paradigm. Reaction times were analyzed using survival data analysis with mixed-effects to take into account both the temporal characteristics of the data and the inter-individual variability in a quantitative manner. First, we found an asymmetrical effect in the masker-target temporal similarity: the target was detected more easily when its tones were longer than those of the masker, but the masker tone duration had a small effect, if any, if it was longer than that of the target. Second, the detection of target was improved for a higher target repetition rate. Finally, the masker uncertainty reduces target detection performance. However, the time and frequency uncertainties have opposite effects, since target detection decreases as the time uncertainty increases and as the frequency uncertainty decreases.

Informational masking is known to produce a large amount of inter-individual variability even larger than for energetic masking [5, 10, 18, 54–56]. In the three experiments, different levels of inter-subject variability were observed. On one hand, in Experiment II, the frailty survival model depicted a significant effect of frailty which reveals a high level of inter-subject heterogeneity in the dynamics of perceptual awareness. On the other hand, in Experiments I and III, no significant effect of the frailty was observed. This result characterizes a higher homogeneity between subjects in these experimental conditions. The inter-subject variability observed in informational masking studies has been explained by the extent to which listeners deal with the stimulus parameters in order to enhance the segregation between the target and the masker and adopt ideal or non-ideal strategies [5]. Since independent and homogeneous groups performed the three experiments, the differences observed in inter-subject processing strategies should be mainly attributed to the specific combination of masker and target properties of each experiment. The interaction between masker uncertainty, masker-target temporal similarity and target repetition rate in the ranges used in Experiment II leads to more variable strategies than that of Experiment I and III. The interaction between the three parameters used in Experiment II increases the inter-individual variability related to the increase in the number of experimental combinations. The small number of stimuli per experimental condition presented to each subject may also be a factor that tends to increase the observed intra- and inter-individual variability.

In order to form coherent representation of the auditory objects, *i.e*. auditory streaming, the auditory system should exploit differences in the statistics of the temporal structure of signals to separate figure from background [57]. At least two complementary phenomena contribute to the release of masking which leads to the detection of the target. First, the acoustic properties of the masker and the target and their statistical differences contribute to the difficulty of the task. Second, the way the auditory system integrates the informational content of the stimulus and extracts relevant information to form the target percept leads to the dynamics of perceptual awareness. The results obtained here allow one to confirm previous results and suggest new perspectives relevant for these two phenomena.

Firstly, this study has manipulated acoustic parameters of a stimulus with the relationships between these parameters allowing one to obtain second order characteristics of the stimulus such as masker uncertainty and masker-target temporal similarity. An acoustic parameter such as the target repetition rate is known to be of critical importance in the grouping of physical and perceptual cues in a complex acoustic scene [22, 58]. In extreme cases, target repetition rate beyond a critical value (about 40 Hz) can cause the sounds to merge into a stream that is easily detected by subjects. In Experiment II and III, the observed effects of target repetition rate on the dynamics of the perceptual awareness are in accordance with the literature [23, 24].

Secondly, in both experiments where masker-target temporal similarity has been manipulated, the observation of its effect leads to the conclusion that perceptual awareness is generally enhanced when the similarity is low. This observation complements previous studies where masker-target frequency similarity [17] and a high degree of temporal similarity [18] highly decreased detection performance. Our study further shows that masker-target temporal similarity effect is asymmetric. Indeed, in Experiment II, the effect was more pronounced when the target duration was larger than the masker tone duration. This result suggests that masker-target duration is very important and salient for organizing auditory scenes, in particular auditory streams, even potentially as important as the effect of the target repetition rate. These two variables, the target tone duration and the target repetition rate may act in concert throughout the silence duration between successive targets. In summary, smaller durations of inter-target silence, associated to higher target repetition rate or higher target duration (for a fixed target repetition rate), promote the build-up of auditory streams.

Thirdly, masker uncertainty has been manipulated using the number of frequencies per octave and the mean inter-tone interval. It has been shown that perceptual awareness is facilitated when the number of frequencies per octave is low. For a given number of frequencies per octave, the perceptual awareness is enhanced for long mean inter-tone intervals. These findings are consistent with previous literature where for example, the frequency uncertainty of individual masker components was predominant in producing the masking effect [10, 59]. Nevertheless, the masker uncertainty, quantified by the entropy of tone distribution, is not linearly related to the facilitation of perceptual awareness. This statistical property of the masker thus does not directly account for the changes in the dynamics of perceptual awareness. Although, stimulus’ statistical characteristics such as uncertainty have been used to explain detection performance [57, 60], we observe here that a acoustical characteristics of the masker such as spectro-temporal density (defined as a number of tones per second and per octave) can account for the changes in the dynamics of perceptual awareness in a more direct manner (see S10 Fig). Nevertheless, it has been shown that other statistical properties of the masker such as the component relative entropy of the masker can account for the characteristics of the informational masking [61, 62]. In this study, all tones have been presented at the same level. The overall level of the masker is then higher for higher spectro-temporal density, which could have influenced the target detection: target is relatively softer and potentially less salient for a high masker spectro-temporal density.

Previous informational masking studies mainly analyzed the results from two-alternative forced choice procedures using concepts from signal detection theory such as auditory discrimination thresholds [11, 14, 63] or detection performance. In the procedure of the present study, we were not able to compute the *d*′ index for the experimental conditions defined by the target properties. Thus, it does not allow us to study the effect of target properties and of interaction between target and noise properties on the detection process. Nevertheless, on the basis of the dynamics of perceptual awareness, we have seen that the relationship between masker and target properties are important, particularly in the type of informational masking paradigm used in the present study. The interaction effect between the number of frequencies per octave and the mean inter-tone interval can be considered as a specific case of the significant effects of the interaction between the stimulus parameters observed here on the dynamics of perceptual awareness. Such an observation suggests that, from the auditory processing point of view, the acoustic scene is not divided into independent parameters and their related effects. However, signal detection theory defines static parameters that do not allow to deal with the progressive integration of information leading to perceptual awareness. Moreover, these dynamic aspects of the detection phenomenon are not taken into account by conventional reaction time analysis methods. In this way, the analysis by survival models with mixed-effects provide a relevant contribution to the study of the dynamics of perceptual awareness by taking into account the respective effects of the stimulus parameters on the detection likelihood.

Finally, it has been proposed that auditory integration of information can be modelled as an evidence accumulation process [64, 65]. Such a model can account for the buildup of the auditory streaming and thus for the dynamics of perceptual awareness. A qualitative comparison between the hazard rate obtained by simulating a simple model of evidence accumulation [65] and those obtained in the present study suggests that the different combinations of masker and target properties lead to different evidence accumulation processes (see S11 Fig). Although these curves are only qualitatively similar to experimental ones, they show the possibility to use survival analysis for the analysis of experimental results in the context of evidence accumulation models. Furthermore, a recent study investigating the neural basis of perceptual consciousness and perceptual monitoring showed that gradual changes in neuronal dynamics during evidence accumulation relates to perceptual consciousness and perceptual monitoring in humans [66]. Thus, the study of auditory perceptual awareness could benefit from the combination of survival and evidence accumulation models with recording of electrophysiological activity at different scales in informational masking tasks to decipher the relationship between conscious auditory perception and neuronal dynamics.

In summary, the masker-target temporal similarity, the target repetition rate and the masker spectro-temporal density parameters of the stimulus modulate the dynamics of perceptual awareness in informational masking. The comparison with an evidence accumulation model shows that these effects may be mediated by changes in the accumulation parameter. Thus our study suggests that the use of survival models to analyse the dynamics of the build-up of auditory perception might be adapted to the experimental testing of the evidence accumulation model. Studying the effects of these stimulus parameters using survival models allows one to study the time course of the detection probability and better determine predictability of signal detection. This study provides useful and novel information on the time course of perceptual awareness related to a set of stimulus parameters for designing further analysis of the dynamics of perceptual awareness. In particular, further studies on possible neuronal correlates of perceptual awareness can be based on this temporal information to investigate how the dynamics of perceptual awareness is causally linked to the dynamical transitions of information transmission at the neuronal population level.

## Supporting information

S1 File Complete statistical analysis of performance and reaction time data for Experiment I. (PDF)

S2 File Complete statistical analysis of performance and reaction time data for Experiment II. (PDF)

S3 File Complete statistical analysis of performance and reaction time data for Experiment III. (PDF)

S1 Fig Detection performance *d*′ for each block and for the three experiments. The performance is lower in the first block for the three experiments and thus their results have been removed from final analysis to minimize a residual learning effect.
(PDF)

S2 Fig Hazard rate functions *h*(*t*) for Experiment I: Similarity × Uncertainty. (PDF)

S3 Fig Cumulative distribtion functions *F*(*t*) for Experiment I: Similarity × Uncertainty. (PDF)

S4 Fig Hazard rate functions *h*(*t*) for Experiment II: Similarity × Rate × Uncertainty. (PDF)

S5 Fig Hazard rate functions *h*(*t*) for Experiment II: Rate × Uncertainty × Similarity. (PDF)

S6 Fig Cumulative distribution functions *F*(*t*) for Experiment II: Similarity × Rate × Uncertainty. (PDF)

S7 Fig Cumulative distribution functions *F*(*t*) for Experiment II: Rate × Uncertainty × Similarity. (PDF)

S8 Fig Hazard rate functions *h*(*t*) for Experiment III: Rate × Uncertainty. (PDF)

S9 Fig Cumulative distribution functions *F*(*t*) for Experiment III: Rate × Uncertainty. (PDF)

S10 FigComparison of the relationship between auditory segregation time constant and entropy (H) with that of auditory segregation time constant and masker spectro-temporal density (STD).Auditory segregation time constant is defined as the time *τ* for which the cumulative distribution function associated to the hazard rate for auditory segregation equals 0.63 (see Panel A.). Masker spectro-temporal density is defined as fpo/miti in s^−1^oct^−1^ where miti: mean inter-tone interval, fpo: frequencies per octave. Panel B. compares the relationship between *τ* and STD or entropy. The data where *τ* > 12 is not depicted.The following table gives the values of masker spectro-temporal density for the set of masker parameters used in the experiments.

**Table:** 

		Exp. I—II	Exp. III		
miti (ms)		800	200	600	1200
Δ (ms)		1400	200	1000	2200
fpo	4	5	—	—	—
	16	—	80	26.7	13.3
	32	40	160	53.3	26.7
	64	80	320	106.7	53.3

miti: mean inter-tone interval, fpo: frequencies per octave.(PDF)

S11 FigHazard rate functions obtained from evidence accumulation model for several values of the parameters.A simple model of evidence accumulation [65] describes activity that accumulates and saturates at target level *T*. The activity *X*_*n*_ is updated sequentially according to: *X*_*n*+1_ = *X*_*n*_ + *r*(*T* − *X*_*n*_) + *ε*_*n*+1_ where *T* < 1 and where $\varepsilon_{n+1}∼N(0,\sigma^{2})$ are independent random variables (Gaussian noise of zero mean and standard deviation *σ*). The activity increments are state dependent and proportional to the difference *T* − *X*_*n*_, with constant rate *r*. Accordingly, the activity *X* drifts towards *T* stochastically if 0 < *r* < 1. Accumulation slows with *X*_*n*_ near *T* and the activity can cross the threshold only due to noise. Examples of the effect of changes in the model parameters. Constant parameters are: *T* = 0.9, *X*_0_ = 0. When not varied, *r* = 0.7 and *σ* = 0.15.(PDF)
