# Supplementary material for: Investigating the influence of masker and target properties on the dynamics of perceptual awareness under informational masking
Source: PLoS One. 2023 Mar 16;18(3):e0282885. doi: 10.1371/journal.pone.0282885 (PMC10019711; doi:10.1371/journal.pone.0282885)
Supplement: S1 File — (PDF) [file pone.0282885.s001.pdf]

Reproducible Report for PONE-D-22-05102 - Investigating the influence of masker and target properties on the dynamics of auditory segregation under informational masking
Experiment I

A. Veyrié and L. Pezard

Thursday 29th September, 2022

Contents

1 Loading data **1**
1.1 Performance (d') . . . 1
1.2 Reaction times . . . 2

2 Qualitative inspection of performance indices and reaction times **3**
2.1 Performance indices distributions . . . 3
2.2 Reaction times distributions . . . 4
2.3 Conclusion . . . 5

3 Detection performance (d') **5**

4 Reaction times **7**
4.1 Time-to-event analysis . . . 8

```
library(nlme)
library(xtable)
library(emmeans)
library(lme4)
library(lmerTest)
library(survival)
library(influence.ME)
source('../utils.R')
```

1 Loading data

1.1 Performance (d')

Loading dataframe

```
perf.all <- read.table("../data/performance_Exp_I.csv", header=TRUE, sep=',',
                        dec=".", fileEncoding="utf-8")[,c(1,3:10)]
# Changing variable to factor
perf.all$Uncertainty <- factor(perf.all$Uncertainty)
perf.all$Sujet <- factor(perf.all$Sujet)
summary(perf.all)
```

```
##      Sujet     Uncertainty     nbhits         nbmiss         nbfa          nbrc
##  1       : 3   29 :14      Min.    : 2   Min.    : 0   Min.   :0.0   Min.   :0
##  2       : 3   115:14      1st Qu.:10   1st Qu.:10   1st Qu.:1.0   1st Qu.:2
##  3       : 3   463:14      Median :24   Median :18   Median :2.0   Median :4
##  4       : 3               Mean   :21   Mean   :19   Mean   :2.6   Mean   :4
##  5       : 3               3rd Qu.:30   3rd Qu.:32   3rd Qu.:4.0   3rd Qu.:6
##  6       : 3               Max.   :42   Max.   :38   Max.   :7.0   Max.   :8
##  (Other):24
##     hitsrate       fasrate         dprime
##  Min.   :0.06   Min.   :0.06   Min.   :-0.58
##  1st Qu.:0.26   1st Qu.:0.19   1st Qu.: 0.12
##  Median :0.52   Median :0.36   Median : 0.32
##  Mean   :0.53   Mean   :0.42   Mean   : 0.37
##  3rd Qu.:0.76   3rd Qu.:0.64   3rd Qu.: 0.69
##  Max.   :0.98   Max.   :0.94   Max.   : 1.90
##
```

1.2 Reaction times

Loading the dataframe

```
rt.all <- read.table("../data/data_Exp_I-bis.csv", header=TRUE,
                      sep=',', dec=".", fileEncoding="utf-8")
# Changing reaction times from msec to sec
rt.all$RT <- rt.all$RT/1000
```

```
# Removing the first bloc (because of learning)
rt.all <- rt.all[which(rt.all$Bloc != 1),]
# Transforming variable into categoric factor
rt.all$Similarity <- factor(rt.all$Similarity)
rt.all$Uncertainty <- factor(rt.all$Uncertainty)
rt.all$Sujet <- factor(rt.all$Sujet)
summary(rt.all)
```

```
##      Sujet           Bloc        Stim                Hits            FA
##  8       : 157   Min.   :2   Length:2072        Min.   :0.00   Min.   :0.00
##  9       : 156   1st Qu.:3   Class :character   1st Qu.:0.00   1st Qu.:0.00
##  10      : 156   Median :4   Mode  :character   Median :0.00   Median :0.00
##  12      : 156   Mean   :4                      Mean   :0.47   Mean   :0.06
##  13      : 156   3rd Qu.:5                      3rd Qu.:1.00   3rd Qu.:0.00
##  14      : 156   Max.   :6                      Max.   :1.00   Max.   :1.00
##  (Other):1135
##       Miss             RC              RT         Similarity     m_ppo
##  Min.   :0.00   Min.   :0.00   Min.   : 0.0   -80 :105    Min.   : 4
##  1st Qu.:0.00   1st Qu.:0.00   1st Qu.: 0.0   -40 :434    1st Qu.: 4
##  Median :0.00   Median :0.00   Median : 1.4   0   :620    Median :16
##  Mean   :0.39   Mean   :0.08   Mean   : 2.5   40  :419    Mean   :28
##  3rd Qu.:1.00   3rd Qu.:0.00   3rd Qu.: 4.0   80  :207    3rd Qu.:64
##  Max.   :1.00   Max.   :1.00   Max.   :12.0   NA's:287    Max.   :64
##
##       m_td            m_iti           t_pi           t_td        m_density
##  Min.   :0.020   Min.   :1500   Min.   : 489   Min.   :0     Min.   : 1.0
##  1st Qu.:0.020   1st Qu.:1500   1st Qu.: 699   1st Qu.:0     1st Qu.: 1.0
##  Median :0.060   Median :1500   Median :1430   Median :0     Median : 5.0
##  Mean   :0.062   Mean   :1500   Mean   :1452   Mean   :0     Mean   : 8.1
```

```
##  3rd Qu.:0.100   3rd Qu.:1500   3rd Qu.:2045   3rd Qu.:0     3rd Qu.:18.0
##  Max.   :0.100   Max.   :1500   Max.   :2924   Max.   :0     Max.   :19.0
##                                 NA's   :287    NA's   :287
##  Uncertainty
##  29 :683
##  115:705
##  463:684
##
##
##
##
```

2 Qualitative inspection of performance indices and reaction times

2.1 Performance indices distributions

Histogram of the all dprime

```
hist(perf.all[,"dprime"], breaks=30, col=c("skyblue"), prob=TRUE,
     xlab="d'", main="")
lines(density(perf.all[,"dprime"]))
abline(v=mean(perf.all[,"dprime"]), col="green", lw=2)
```

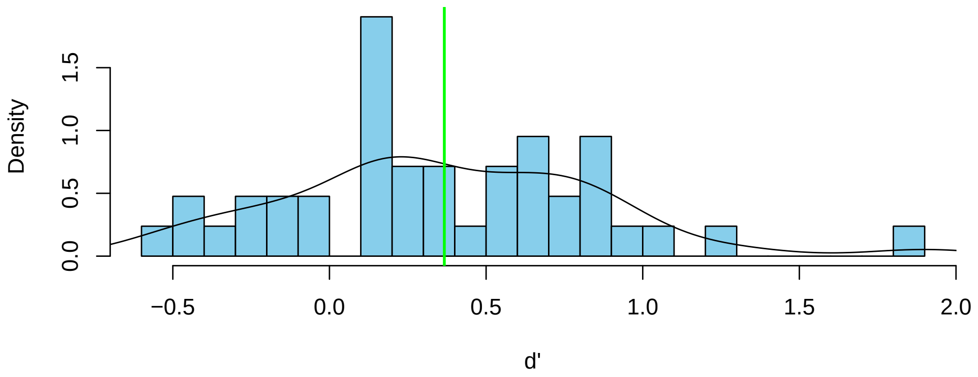

Boxplot of performances indices values by subject

```
par(mfrow=c(3,1))
boxplot(hitsrate ~ Sujet, col=c("skyblue"), data=perf.all,
        xlab="Subject", ylab="Hit's rate", main="")
abline(h=mean(perf.all[,"hitsrate"]), col="green", lw=2)
boxplot(fasrate ~ Sujet, col=c("skyblue"), data=perf.all,
        xlab="Subject", ylab="False alarm's rate", main="")
abline(h=mean(perf.all[,"fasrate"]), col="green", lw=2)
boxplot(dprime ~ Sujet,
        col=c("skyblue"), data=perf.all, xlab="Subject", ylab="d'",
        main="")
abline(h=mean(perf.all[,"dprime"]), col="green", lw=2)
```

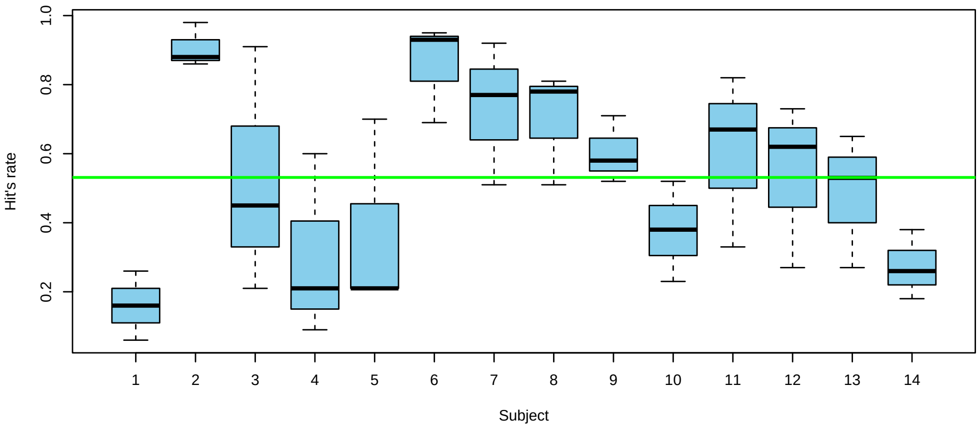

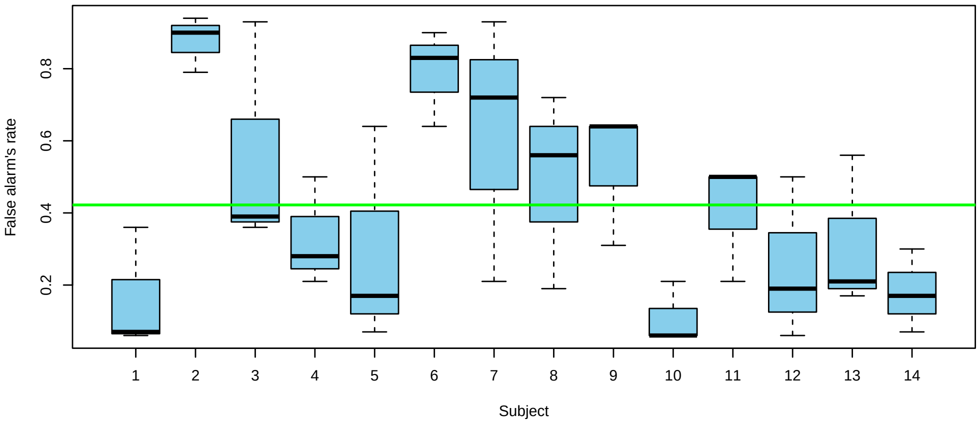

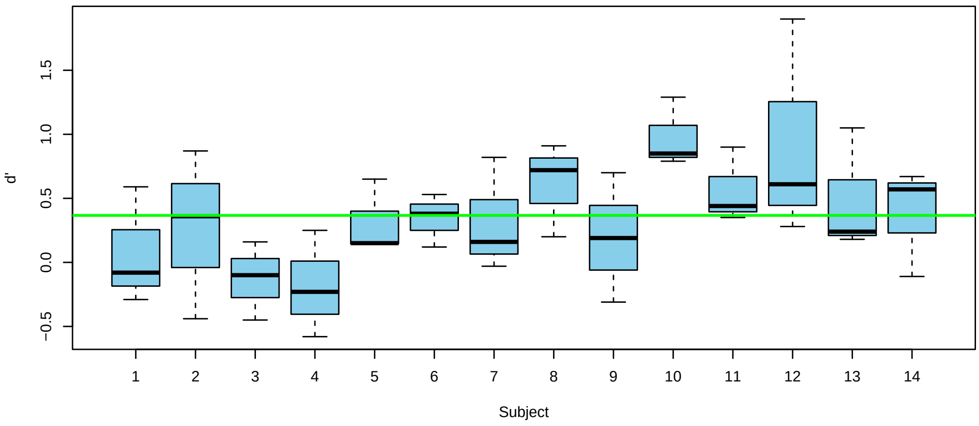

2.2 Reaction times distributions

Ploting the reaction times distribution

```
# RT < 1.6 sec. are before the second target tone
rt.hits <- rt.all[which(rt.all$Hits == 1 & rt.all$RT > 1.6),]
hist(rt.hits[,"RT"], breaks=50, col=c("skyblue"), prob=TRUE, xlab="DT (ms)",
```

```
     main="")
lines(density(rt.hits[,"RT"]))
abline(v=mean(rt.hits[,"RT"]), col="green")
```

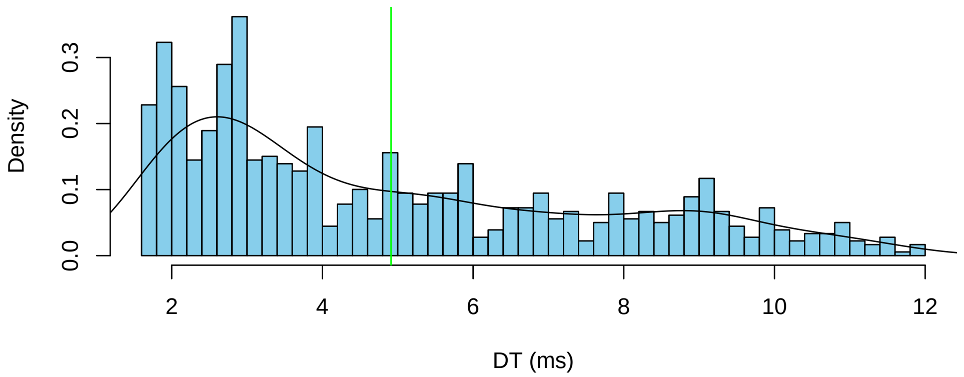

Boxplot of reaction times values by subject

```
boxplot(RT ~ Sujet,
        col=c("skyblue"), data=rt.hits, xlab="Subject", ylab="DT", main="")
abline(h=mean(rt.hits[,"RT"]), col="green")
```

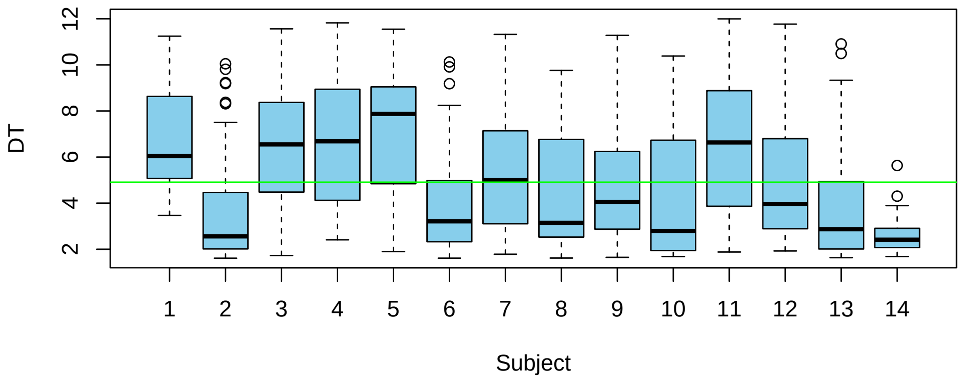

2.3 Conclusion

Although there is significant variability, there is no clear evidence to consider excluding subjects at this stage.

3 Detection performance (d')

```
bwplot(dprime~Uncertainty, data=perf.all)
```

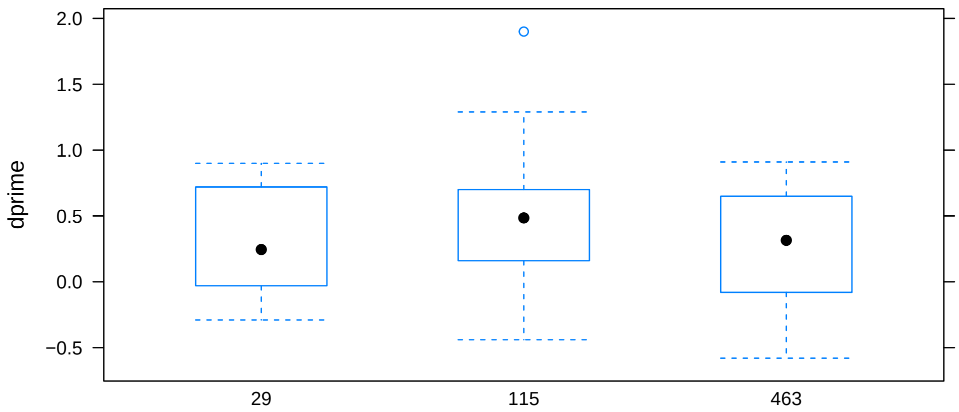

Fitting a mixed effects linear model

```
model_uncertainty <- lmer(dprime ~ Uncertainty + (1|Sujet), data=perf.all)
plot.lmer.diagnostics(model_uncertainty, perf.all)
```

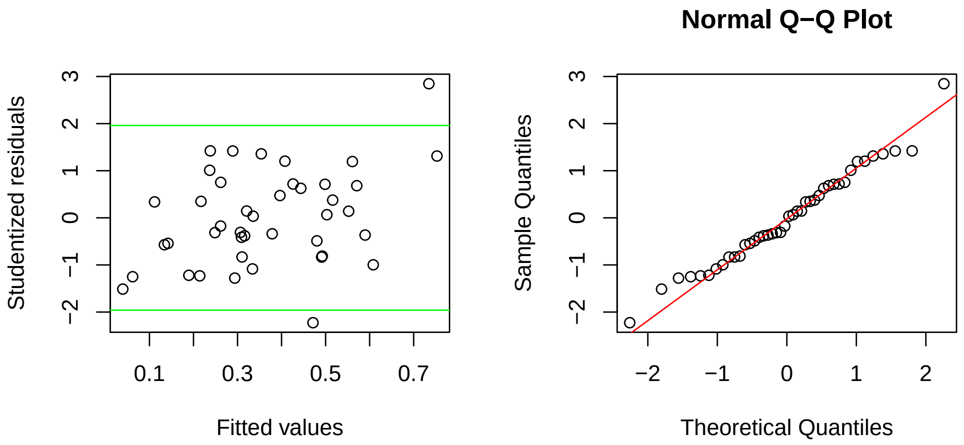


```
plot.lmer.influence(model_uncertainty, perf.all)
```

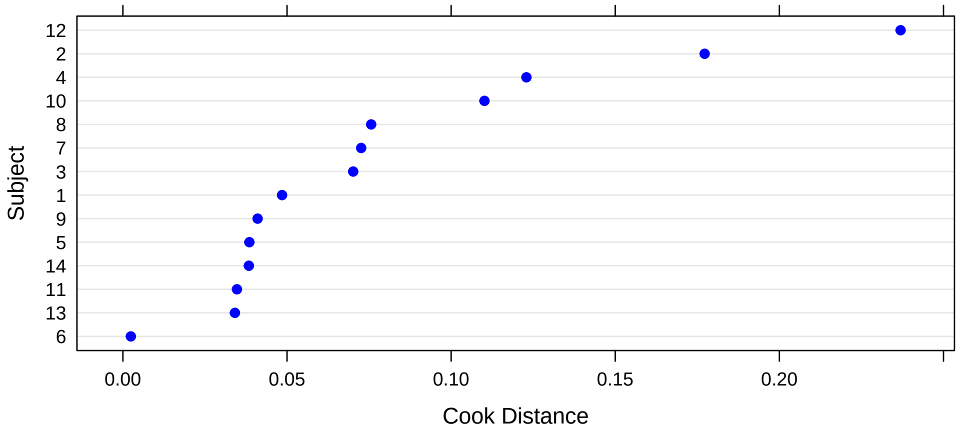

```
summary(model_uncertainty)

## Linear mixed model fit by REML. t-tests use Satterthwaite's method [
## lmerModLmerTest]
## Formula: dprime ~ Uncertainty + (1 | Sujet)
##    Data: perf.all
##
## REML criterion at convergence: 64
##
## Scaled residuals:
##     Min      1Q  Median      3Q     Max
## -1.9990 -0.6751 -0.0626  0.6324  2.5564
##
## Random effects:
##  Groups   Name        Variance Std.Dev.
##  Sujet    (Intercept) 0.0452   0.213
##  Residual             0.2079   0.456
## Number of obs: 42, groups:  Sujet, 14
##
## Fixed effects:
##                 Estimate Std. Error      df t value Pr(>|t|)
## (Intercept)       0.3300     0.1344 36.6616    2.45    0.019 *
## Uncertainty115    0.1821     0.1723 26.0000    1.06    0.300
## Uncertainty463   -0.0721     0.1723 26.0000   -0.42    0.679
## ---
## Signif. codes:  0 '***' 0.001 '**' 0.01 '*' 0.05 '.' 0.1 ' ' 1
##
## Correlation of Fixed Effects:
##             (Intr) Unc115
## Uncrtnty115 -0.641
## Uncrtnty463 -0.641  0.500
```

```
anova(model_uncertainty)

## Type III Analysis of Variance Table with Satterthwaite's method
##             Sum Sq Mean Sq NumDF DenDF F value Pr(>F)
## Uncertainty  0.481    0.24     2    26    1.16   0.33
```

```
# Figure 2.A.
pdf("../../manuscript/figures/results/Exp-I/performance.pdf", width=3.5, height=3.5)
par(oma=c(0,0,0,0), mar=c(4,4,2,0)+0.1)
boxplot(dprime~Uncertainty, data=perf.all, ylim=c(-0.5,4.5),
        main="Experiment I", xlab="Uncertainty (nats)", ylab="d'")
dev.off()
```

4 Reaction times

For comparison with d' results:

```
boxplot(RT~Uncertainty, data=rt.hits,
        main="Experiment I", xlab="Uncertainty (nats)")
```

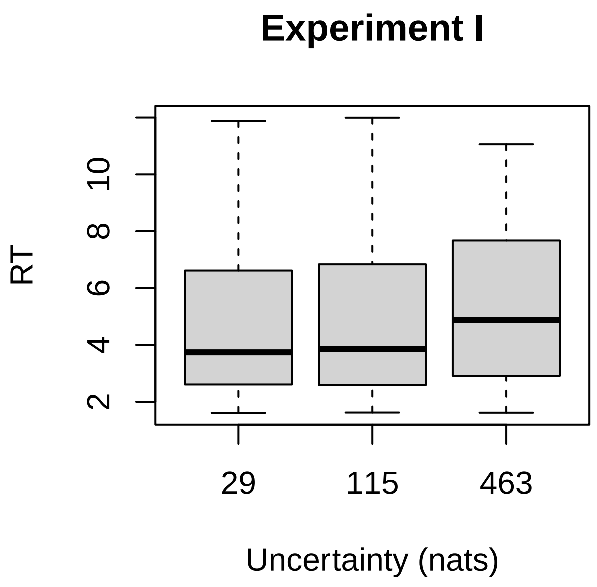


```
bwplot(RT~Similarity|Uncertainty, data=rt.hits, layout=c(3,1))
```

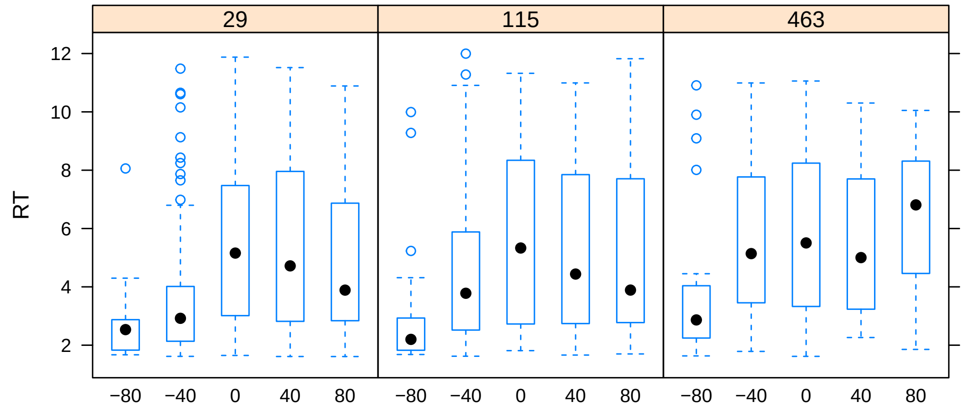

4.1 Time-to-event analysis

```
tte.data <- rbind(rt.all[which(rt.all$Hits == 1 & rt.all$RT > 1.6),],
                  rt.all[which(rt.all$Miss == 1),])
summary(tte.data)

##       Sujet          Bloc         Stim                 Hits             FA
##   7       :135   Min.   :2   Length:1704        Min.   :0.00   Min.   :0
##   12      :135   1st Qu.:3   Class :character   1st Qu.:0.00   1st Qu.:0
##   10      :134   Median :4   Mode  :character   Median :1.00   Median :0
##   8       :132   Mean   :4                      Mean   :0.53   Mean   :0
##   9       :129   3rd Qu.:5                      3rd Qu.:1.00   3rd Qu.:0
##   13      :129   Max.   :6                      Max.   :1.00   Max.   :0
##   (Other):910
```

```
##       Miss             RC          RT       Similarity     m_ppo
##  Min.   :0.00   Min.   :0   Min.   : 0.0   -80: 91    Min.   : 4
##  1st Qu.:0.00   1st Qu.:0   1st Qu.: 0.0   -40:417    1st Qu.: 4
##  Median :0.00   Median :0   Median : 1.8   0  :592    Median :16
##  Mean   :0.47   Mean   :0   Mean   : 2.6   40 :401    Mean   :29
##  3rd Qu.:1.00   3rd Qu.:0   3rd Qu.: 4.3   80 :203    3rd Qu.:64
##  Max.   :1.00   Max.   :0   Max.   :12.0              Max.   :64
##
##       m_td            m_iti           t_pi           t_td         m_density
##  Min.   :0.020   Min.   :1500   Min.   : 489   Min.   :0.020   Min.   : 1.0
##  1st Qu.:0.020   1st Qu.:1500   1st Qu.: 699   1st Qu.:0.020   1st Qu.: 1.0
##  Median :0.060   Median :1500   Median :1430   Median :0.060   Median : 5.0
##  Mean   :0.063   Mean   :1500   Mean   :1454   Mean   :0.058   Mean   : 8.4
##  3rd Qu.:0.100   3rd Qu.:1500   3rd Qu.:2045   3rd Qu.:0.100   3rd Qu.:18.0
##  Max.   :0.100   Max.   :1500   Max.   :2924   Max.   :0.100   Max.   :19.0
##
##  Uncertainty
##  29 :542
##  115:572
##  463:590
##
##
##
##
```

```
# ADD THIS!
tte.data$RT[which(tte.data$Miss == 1)] <- 12

survie <- Surv(tte.data$RT, tte.data$Hits)
model.wf <- coxph(survie ~ Uncertainty * Similarity +
                  frailty(Sujet, distribution='gaussian'), data=tte.data)
plot.cox.diagnostics(model.wf, tte.data)
```

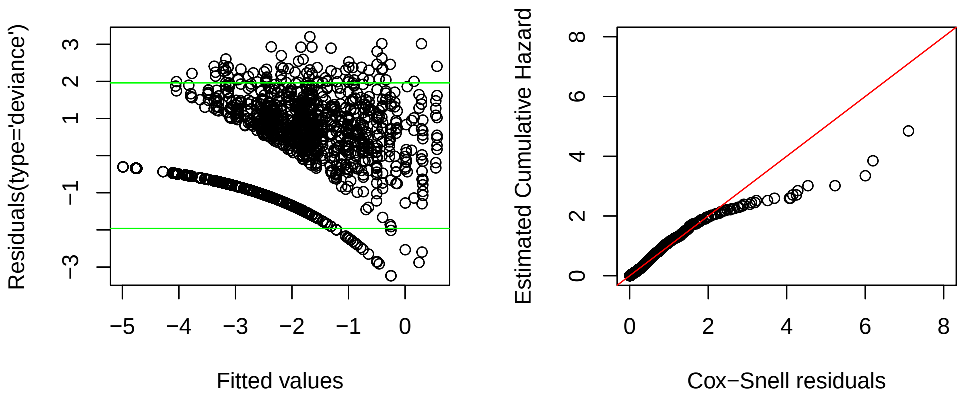


```
plot.cox.influence(model.wf, tte.data)
```

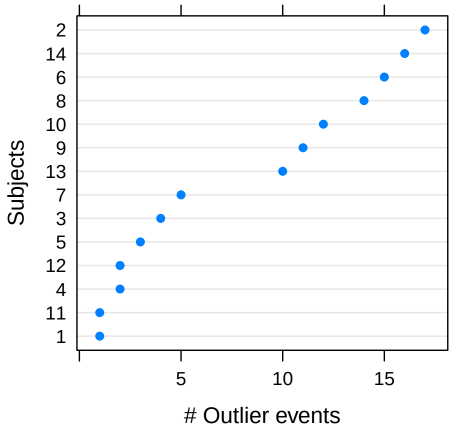

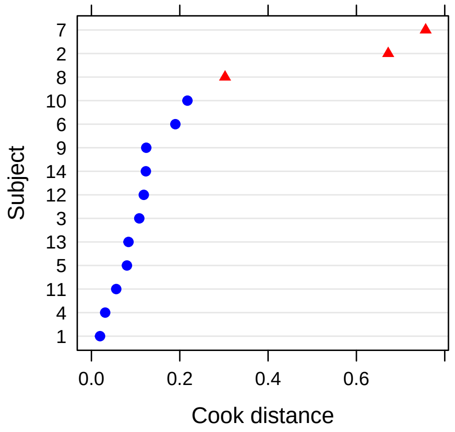

No clear argument from individual performances indices ou reaction time distribution allow to discard any of the 3 influential subjects (S2, S7, S8).

The total number of events (hits) is: 898 and the average number of event per subject is 64.14 and the average number of event per modality is 59.87. 4.28.

```
summary(model.wf)

## Call:
## coxph(formula = survie ~ Uncertainty * Similarity + frailty(Sujet,
##     distribution = "gaussian"), data = tte.data)
##
##   n= 1704, number of events= 898
##
##                           coef   se(coef) se2   Chisq  DF   p
## Uncertainty115           -0.297 0.269    0.269   1.22   1.0 2.7e-01
## Uncertainty463           -0.806 0.263    0.263   9.40   1.0 2.2e-03
## Similarity-40            -1.038 0.225    0.225  21.34   1.0 3.9e-06
## Similarity0              -2.000 0.225    0.225  79.00   1.0 6.2e-19
## Similarity40             -1.763 0.228    0.228  59.56   1.0 1.2e-14
## Similarity80             -1.313 0.246    0.245  28.62   1.0 8.8e-08
## frailty(Sujet, distributi                        446.55 13.6 8.5e-87
## Uncertainty115:Similarity -0.518 0.305    0.304   2.89   1.0 8.9e-02
## Uncertainty463:Similarity -0.721 0.304    0.304   5.64   1.0 1.8e-02
## Uncertainty115:Similarity -0.308 0.306    0.306   1.02   1.0 3.1e-01
## Uncertainty463:Similarity -0.481 0.313    0.313   2.36   1.0 1.2e-01
## Uncertainty115:Similarity -0.287 0.315    0.315   0.83   1.0 3.6e-01
## Uncertainty463:Similarity -0.699 0.333    0.333   4.39   1.0 3.6e-02
## Uncertainty115:Similarity -0.451 0.341    0.341   1.75   1.0 1.9e-01
## Uncertainty463:Similarity -1.392 0.400    0.400  12.10   1.0 5.0e-04
##
##                           exp(coef) exp(-coef) lower .95 upper .95
## Uncertainty115                0.743       1.35    0.4388     1.258
## Uncertainty463                0.447       2.24    0.2670     0.748
## Similarity-40                 0.354       2.82    0.2279     0.550
## Similarity0                   0.135       7.39    0.0871     0.210
## Similarity40                  0.172       5.83    0.1096     0.268
## Similarity80                  0.269       3.72    0.1662     0.435
## Uncertainty115:Similarity     0.596       1.68    0.3281     1.082
## Uncertainty463:Similarity     0.486       2.06    0.2681     0.882
## Uncertainty115:Similarity     0.735       1.36    0.4032     1.338
```

```
## Uncertainty463:Similarity     0.618       1.62    0.3344     1.142
## Uncertainty115:Similarity     0.751       1.33    0.4046     1.393
## Uncertainty463:Similarity     0.497       2.01    0.2588     0.956
## Uncertainty115:Similarity     0.637       1.57    0.3265     1.243
## Uncertainty463:Similarity     0.249       4.02    0.1135     0.545
##
## Iterations: 5 outer, 16 Newton-Raphson
##      Variance of random effect= 0.62
## Degrees of freedom for terms=  2.0  4.0 13.5  8.0
## Concordance= 0.783  (se = 0.007 )
## Likelihood ratio test= 885  on 27.5 df,   p=<2e-16
```

```
anova(model.wf)

## Analysis of Deviance Table
##  Cox model: response is survie
## Terms added sequentially (first to last)
##
##                                          loglik  Chisq   Df Pr(>|Chi|)
## NULL                                      -6388
## Uncertainty                               -6304 167.07  2.0     <2e-16 ***
## Similarity                                -6161 286.46  4.0     <2e-16 ***
## frailty(Sujet, distribution = "gaussian")  -6161   0.27  1.0       0.61
## Uncertainty:Similarity                    -5945 430.98 20.5     <2e-16 ***
## ---
## Signif. codes:  0 '***' 0.001 '**' 0.01 '*' 0.05 '.' 0.1 ' ' 1
```

```
emmip(model.wf, Similarity ~ Uncertainty, engine='lattice')
```

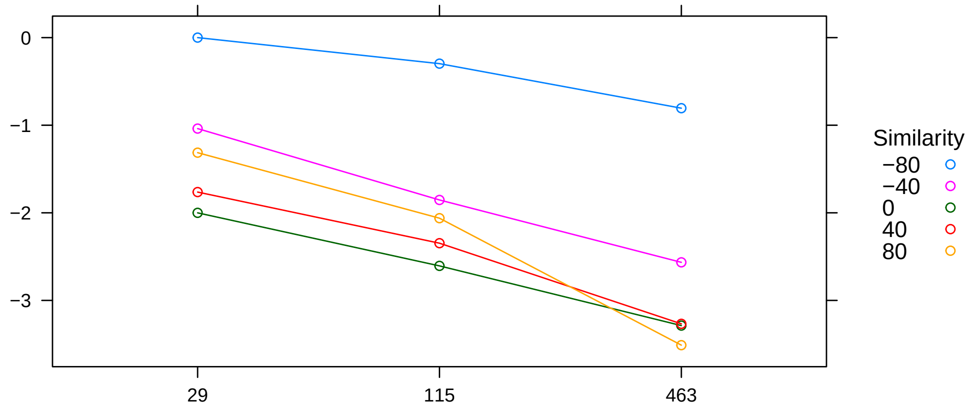


```
emmip(model.wf, Uncertainty ~ Similarity, engine='lattice')
```

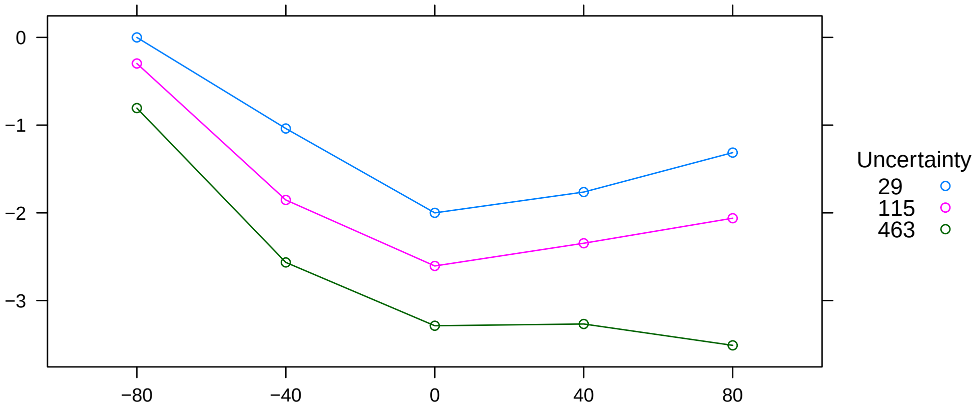


```
emm <- emmeans(model.wf, specs=pairwise~Uncertainty:Similarity)
pwpm(emm$emmeans)

##            29 -80  115 -80  463 -80   29 -40  115 -40  463 -40     29 0    115 0
## 29 -80   [ 0.000]   0.9989   0.1291   0.0004   <.0001   <.0001   <.0001   <.0001
## 115 -80    0.2971 [-0.297]   0.8103   0.0461   <.0001   <.0001   <.0001   <.0001
## 463 -80    0.8056   0.5085 [-0.806]   0.9985   <.0001   <.0001   <.0001   <.0001
## 29 -40     1.0384   0.7413   0.2327 [-1.038]   <.0001   <.0001   <.0001   <.0001
## 115 -40    1.8532   1.5561   1.0476   0.8148 [-1.853]   0.0004   0.9994   <.0001
## 463 -40    2.5652   2.2681   1.7595   1.5268   0.7119 [-2.565]   0.0149   1.0000
## 29 0       2.0002   1.7030   1.1945   0.9618   0.1469  -0.5650 [-2.000]   0.0034
## 115 0      2.6058   2.3086   1.8001   1.5674   0.7525   0.0406   0.6056 [-2.606]
## 463 0      3.2871   2.9900   2.4815   2.2487   1.4339   0.7219   1.2870   0.6814
## 29 40      1.7631   1.4660   0.9574   0.7247  -0.0902  -0.8021  -0.2371  -0.8427
## 115 40     2.3467   2.0496   1.5411   1.3083   0.4935  -0.2185   0.3466  -0.2590
## 463 40     3.2672   2.9701   2.4616   2.2288   1.4140   0.7020   1.2671   0.6615
## 29 80      1.3134   1.0163   0.5077   0.2750  -0.5398  -1.2518  -0.6868  -1.2924
## 115 80     2.0614   1.7643   1.2557   1.0230   0.2082  -0.5038   0.0612  -0.5444
## 463 80     3.5107   3.2136   2.7051   2.4723   1.6575   0.9455   1.5106   0.9050
##             463 0    29 40   115 40   463 40    29 80   115 80   463 80
## 29 -80     <.0001   <.0001   <.0001   <.0001   <.0001   <.0001   <.0001
## 115 -80    <.0001   <.0001   <.0001   <.0001   0.0019   <.0001   <.0001
## 463 -80    <.0001   0.0004   <.0001   <.0001   0.6390   <.0001   <.0001
## 29 -40     <.0001   <.0001   <.0001   <.0001   0.9609   <.0001   <.0001
## 115 -40    <.0001   1.0000   0.1587   <.0001   0.1361   0.9989   <.0001
## 463 -40    0.0074   <.0001   0.9956   0.0638   <.0001   0.3836   0.0781
## 29 0       <.0001   0.9371   0.6812   <.0001   0.0049   1.0000   <.0001
## 115 0      0.0141   <.0001   0.9739   0.1014   <.0001   0.2357   0.1135
## 463 0    [-3.287]   <.0001   <.0001   1.0000   <.0001   <.0001   1.0000
## 29 40     -1.5240 [-1.763]   0.0318   <.0001   0.3930   0.9622   <.0001
## 115 40    -0.9404   0.5836 [-2.347]   0.0023   <.0001   0.9866   0.0077
## 463 40    -0.0199   1.5042   0.9205 [-3.267]   <.0001   <.0001   1.0000
## 29 80     -1.9737  -0.4497  -1.0333  -1.9538 [-1.313]   0.0301   <.0001
## 115 80    -1.2257   0.2983  -0.2853  -1.2058   0.7480 [-2.061]   0.0003
## 463 80     0.2236   1.7476   1.1640   0.2435   2.1973   1.4493 [-3.511]
##
## Row and column labels: Uncertainty:Similarity
## Upper triangle: P values   adjust = "tukey"
## Diagonal: [Estimates] (emmean)
```

```
## Lower triangle: Comparisons (estimate)   earlier vs. later

pwpp(emm$emmeans)
```

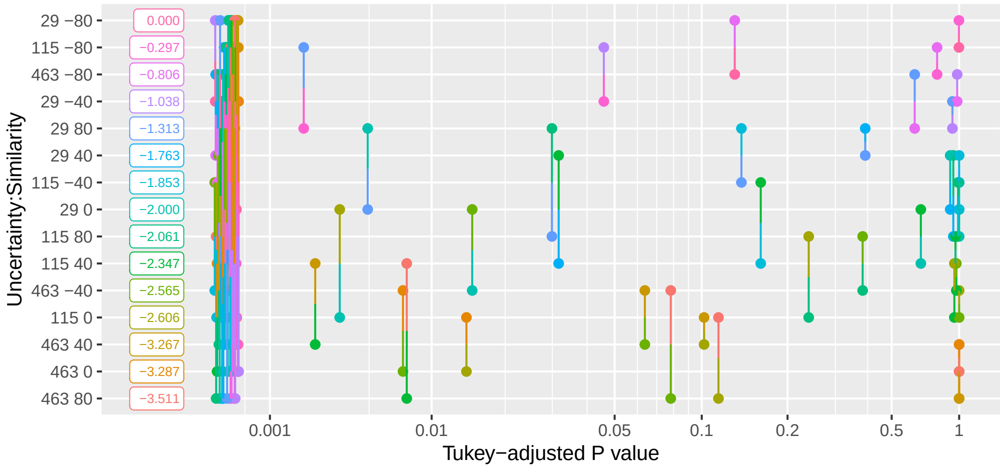


```
# Table 4
emm.cld <- cld(emm$emmeans)

## Error in cld(emm$emmeans):  impossible de trouver la fonction "cld"

plot(emm.cld)

## Error in plot(emm.cld):  objet 'emm.cld' introuvable

emm.cld

## Error in eval(expr, envir, enclos):  objet 'emm.cld' introuvable
```
