# Supplementary material for: Investigating the influence of masker and target properties on the dynamics of perceptual awareness under informational masking
Source: PLoS One. 2023 Mar 16;18(3):e0282885. doi: 10.1371/journal.pone.0282885 (PMC10019711; doi:10.1371/journal.pone.0282885)
Supplement: S2 File — (PDF) [file pone.0282885.s002.pdf]

Reproducible Report for PONE-D-22-05102 - Investigating the influence of masker and target properties on the dynamics of perceptual awareness under informational masking
Experiment II

A. Veyrié and L. Pezard

Thursday 29th September, 2022

Contents

1 Loading data **1**
1.1 Preformance data (d') . . . 1
1.2 Reaction times . . . 2

2 Qualitative inspection of performance indices and reaction times **3**
2.1 Performance indices distributions . . . 3
2.2 Reaction times distribution . . . 4
2.3 Conclusion . . . 6

3 Detection performance (d') **6**

4 Reaction times **12**
4.1 Time-to-event analysis . . . 16


```
library(nlme)
library(xtable)
library(emmeans)
library(lme4)
library(lmerTest)
library(survival)
library(influence.ME)
library(multcomp)
#library(dplyr)
#library(lattice)
source('../utils.R')
```

1 Loading data

1.1 Preformance data (d')

Loading dataframe

```
perf.all = read.table("../data/performance_Exp_II.csv",
                      header=TRUE, sep=',', dec=".",
                      fileEncoding="utf-8")
```

```r
# Changing variable in categoric factor
perf.all$Uncertainty = factor(perf.all$Uncertainty)
summary(perf.all)
```

```
##      Sujet           Exp             Uncertainty     nbhits        nbmiss
##  Min.   : 1.0   Length:42          29 :14      Min.   : 9   Min.   : 0
##  1st Qu.: 4.0   Class :character   115:14      1st Qu.:31   1st Qu.: 2
##  Median : 7.5   Mode  :character   463:14      Median :39   Median : 6
##  Mean   : 7.5                                  Mean   :36   Mean   : 9
##  3rd Qu.:11.0                                  3rd Qu.:42   3rd Qu.:14
##  Max.   :14.0                                  Max.   :45   Max.   :35
##       nbfa            nbrc         hitsrate        fasrate          dprime
##  Min.   : 0.0   Min.   : 0.0   Min.   :0.21   Min.   :0.02   Min.   :-0.8
##  1st Qu.: 1.0   1st Qu.: 6.0   1st Qu.:0.68   1st Qu.:0.06   1st Qu.: 0.5
##  Median : 4.0   Median :17.5   Median :0.86   Median :0.21   Median : 1.6
##  Mean   : 7.1   Mean   :14.3   Mean   :0.79   Mean   :0.35   Mean   : 1.6
##  3rd Qu.:14.0   3rd Qu.:20.0   3rd Qu.:0.94   3rd Qu.:0.69   3rd Qu.: 2.8
##  Max.   :25.0   Max.   :25.0   Max.   :0.99   Max.   :0.98   Max.   : 4.4
```

1.2 Reaction times

Loading the dataframe

```r
rt.all = read.table("../data/data_Exp_II.csv",
                  header=TRUE, sep=',', dec=".", fileEncoding="utf-8")
# Removing the first bloc (because of learning)
rt.all = rt.all[which(rt.all$Bloc != 1),]
# Changing reaction times from msec to sec
rt.all$RT = rt.all$RT/1000
```

```r
# Transforming variable into categoric factor
rt.all$Sujet = factor(rt.all$Sujet)
rt.all$Similarity = factor(rt.all$Similarity)
rt.all$Uncertainty = factor(rt.all$Uncertainty)
rt.all$T.Rate = factor(rt.all$T.Rate)
summary(rt.all)
```

```
##      Sujet           Bloc        Stim                 Hits             FA
##  1      : 200   Min.   :2   Length:2800        Min.   :0.00   Min.   :0.00
##  2      : 200   1st Qu.:3   Class :character   1st Qu.:0.00   1st Qu.:0.00
##  3      : 200   Median :4   Mode  :character   Median :1.00   Median :0.00
##  4      : 200   Mean   :4                      Mean   :0.54   Mean   :0.11
##  5      : 200   3rd Qu.:5                      3rd Qu.:1.00   3rd Qu.:0.00
##  6      : 200   Max.   :6                      Max.   :1.00   Max.   :1.00
##  (Other):1600
##       Miss             RC              RT         Similarity     m_ppo
##  Min.   :0.00   Min.   :0.00   Min.   : 0.0   0    :627   Min.   : 4
##  1st Qu.:0.00   1st Qu.:0.00   1st Qu.: 0.0   40   :632   1st Qu.: 4
##  Median :0.00   Median :0.00   Median : 1.4   80   :631   Median :16
##  Mean   :0.13   Mean   :0.22   Mean   : 2.1   NA's:910    Mean   :27
##  3rd Qu.:0.00   3rd Qu.:0.00   3rd Qu.: 2.7               3rd Qu.:64
##  Max.   :1.00   Max.   :1.00   Max.   :12.0               Max.   :64
##
##       m_td            m_iti            t_pi         T.Rate        t_td
##  Min.   :0.020   Min.   :1500   Min.   : 489   5    :626   Min.   :0
```

```
##  1st Qu.:0.020   1st Qu.:1500   1st Qu.: 699   10  :639   1st Qu.:0
##  Median :0.060   Median :1500   Median :1430   20  :625   Median :0
##  Mean   :0.059   Mean   :1500   Mean   :1436   NA's:910   Mean   :0
##  3rd Qu.:0.100   3rd Qu.:1500   3rd Qu.:2045              3rd Qu.:0
##  Max.   :0.100   Max.   :1500   Max.   :2924              Max.   :0
##                                 NA's   :910               NA's   :910
##    m_density    Uncertainty
##  Min.   : 1.0   29 :978
##  1st Qu.: 1.0   115:912
##  Median : 5.0   463:910
##  Mean   : 7.9
##  3rd Qu.:18.0
##  Max.   :19.0
##
```

2 Qualitative inspection of performance indices and reaction times

2.1 Performance indices distributions

Histogram of the all dprime

```r
hist(perf.all[,"dprime"],
     breaks=30, col=c("skyblue"), prob=TRUE, xlab="d'", main="")
lines(density(perf.all[,"dprime"]))
abline(v=mean(perf.all[,"dprime"]), col="green")
```

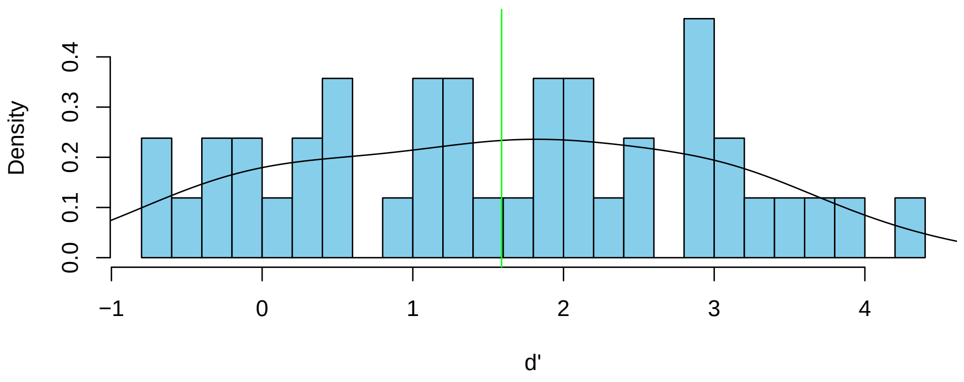

Boxplot of performance indices by subject

```r
par(mfrow=c(3,1))
boxplot(hitsrate ~ Sujet, col=c("skyblue"), data=perf.all,
        xlab="Subject", ylab="Hit's rate", main="")
abline(h=mean(perf.all[,"hitsrate"]), col="green")
boxplot(fasrate ~ Sujet, col=c("skyblue"), data=perf.all,
        xlab="Subject", ylab="False alarm's rate", main="")
abline(h=mean(perf.all[,"fasrate"]), col="green")
boxplot(dprime ~ Sujet,
        col=c("skyblue"), data=perf.all, xlab="Subject", ylab="d'", main="")
```

```
abline(h=mean(perf.all[,"dprime"]), col="green")
```

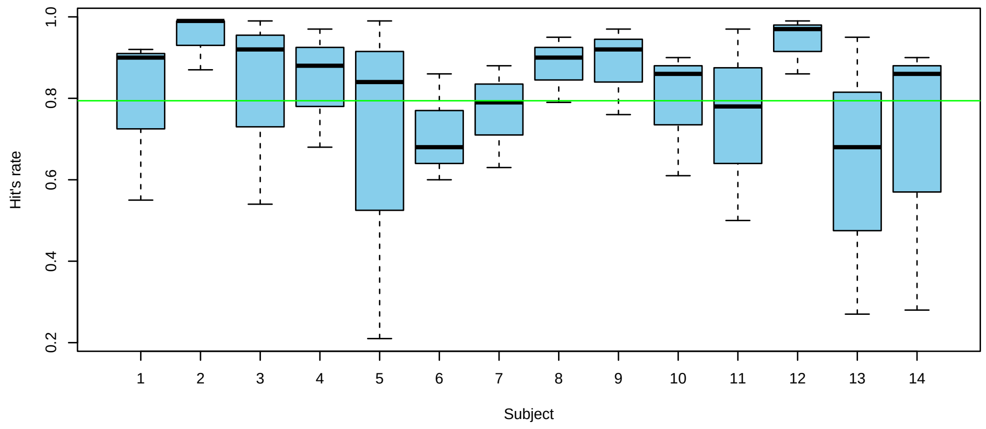

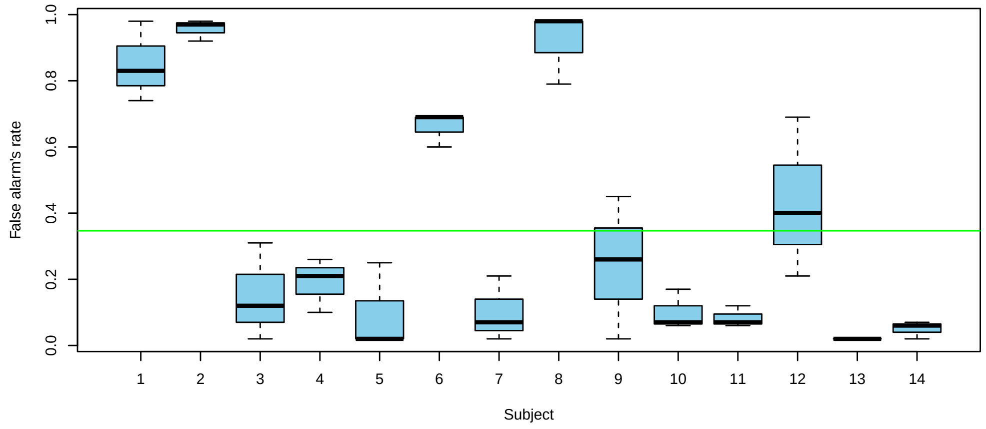

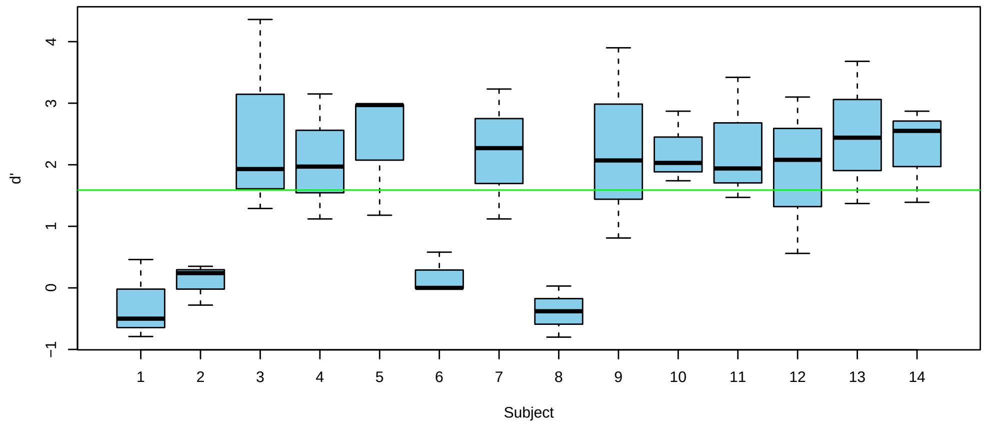

2.2 Reaction times distribution

Ploting the reaction times distribution

```
# Selectioning reaction time for Hits from a cut of 700 ms
rt.hits <- rt.all[which(rt.all$RT > 0.7 & rt.all$Hits == 1),]
hist(rt.hits[,"RT"], breaks=50, col=c("skyblue"),
     prob=TRUE, xlab="TD (ms)", main="")
lines(density(rt.hits[,"RT"]))
abline(v=mean(rt.hits[,"RT"]), col="green")
```

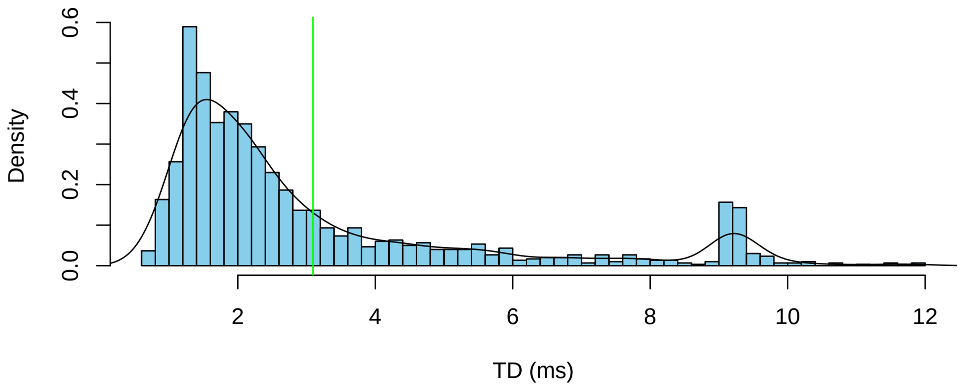

Boxplot of dprime values by subject

```
boxplot(RT ~ Sujet,
        col=c("skyblue"), data=rt.hits, xlab="Subject", ylab="DT", main="")
abline(h=mean(rt.hits[,"RT"]), col="green")
```

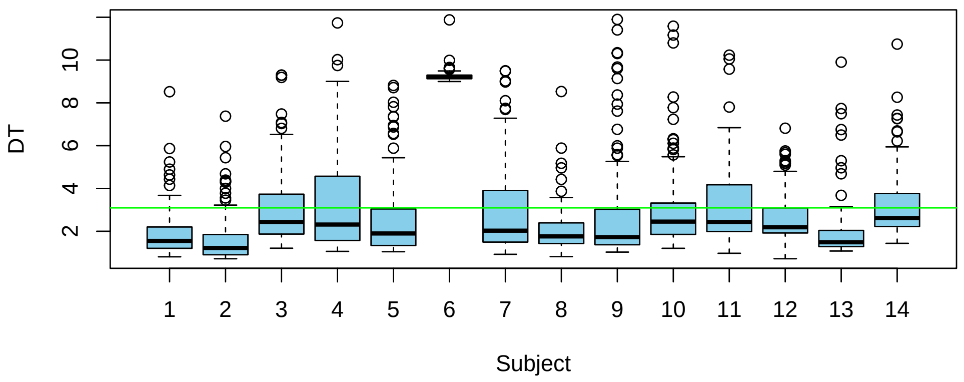


```
rt_wo6 <- rt.hits[which(rt.hits$Sujet != 6),]
hist(rt.hits[,"RT"], breaks=50, col=c("red"),
     prob=TRUE, xlab="TD (ms)", main="", ylim=c(0,0.7))
hist(rt_wo6[,"RT"], breaks=50, col=c("skyblue"),
     prob=TRUE, xlab="TD (ms)", main="", add=TRUE)
arrows(10, 0.4, 9.3, 0.22)
text(10.3, 0.4, "S6")
```

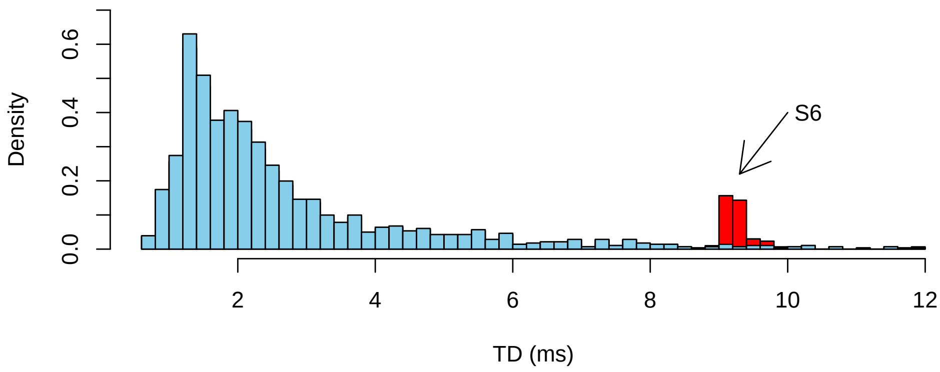

2.3 Conclusion

Remove subject n°6

```
rt.wos = rt.hits[which(rt.hits$Sujet != 6),]
perf.wos = perf.all[which(perf.all$Sujet != 6),]
tte.wos = rt.all[which(rt.all$Sujet != 6),]
```

3 Detection performance (d')

```
bwplot(dprime~Uncertainty, data=perf.wos)
```

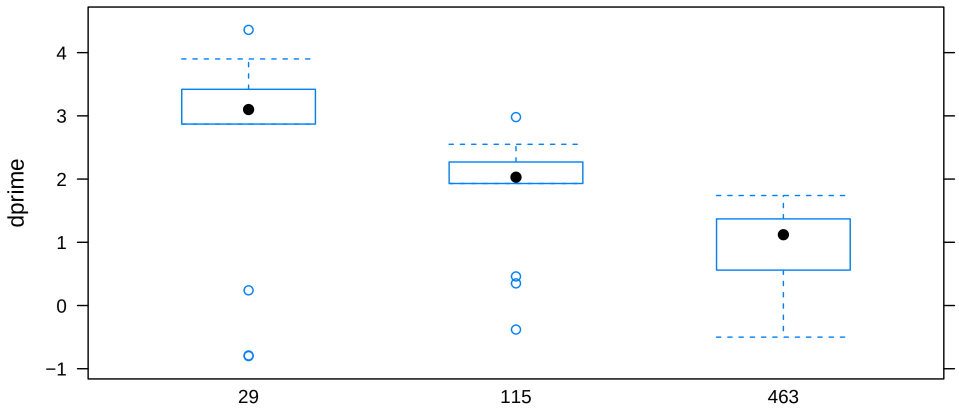


```
# boxplot(dprime~Uncertainty, data=perf.wos)
```

```
model_uncertainty = lmer(dprime ~ Uncertainty + (1|Sujet), data=perf.wos)
plot.lmer.diagnostics(model_uncertainty, perf.wos)
```

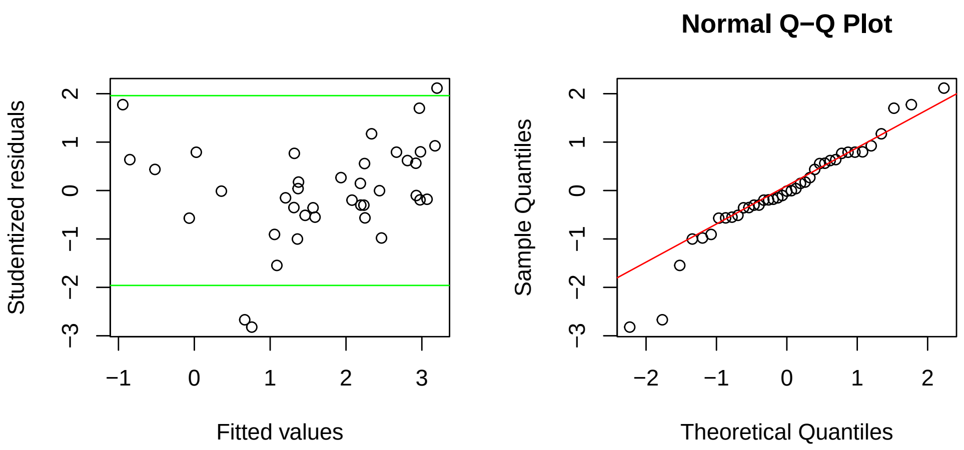


```
plot.lmer.influence(model_uncertainty, perf.wos)
```

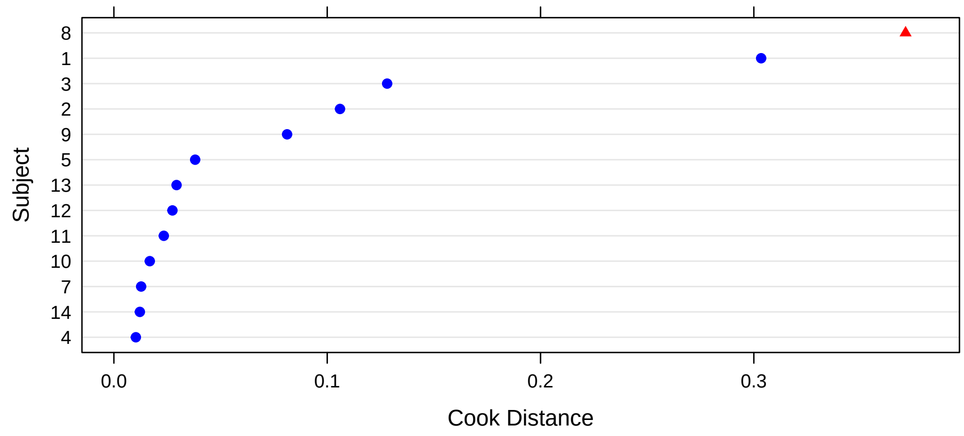


```
perf.wos = perf.wos[which(perf.wos$Sujet != 8),]
model_uncertainty = lmer(dprime ~ Uncertainty + (1|Sujet), data=perf.wos)
plot.lmer.diagnostics(model_uncertainty, perf.wos)
```

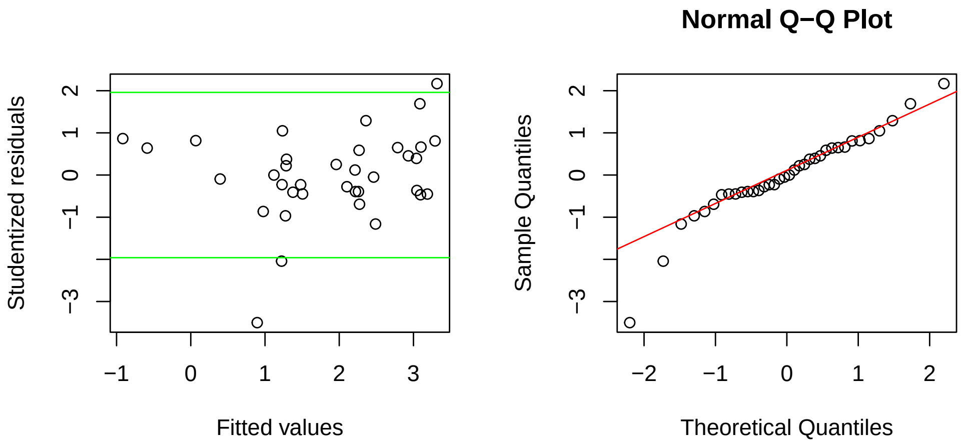

```
plot.lmer.influence(model_uncertainty, perf.wos)
```

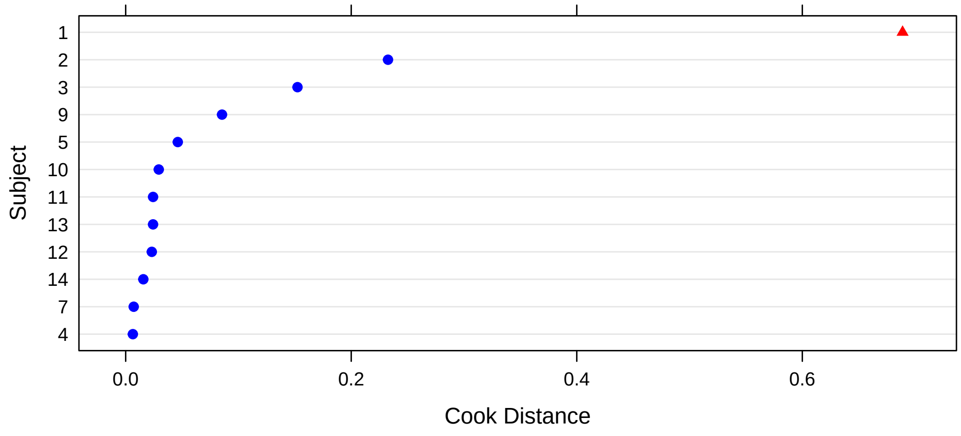


```
perf.wos = perf.wos[which(perf.wos$Sujet != 1),]
model_uncertainty = lmer(dprime ~ Uncertainty + (1|Sujet), data=perf.wos)
plot.lmer.diagnostics(model_uncertainty, perf.wos)
```

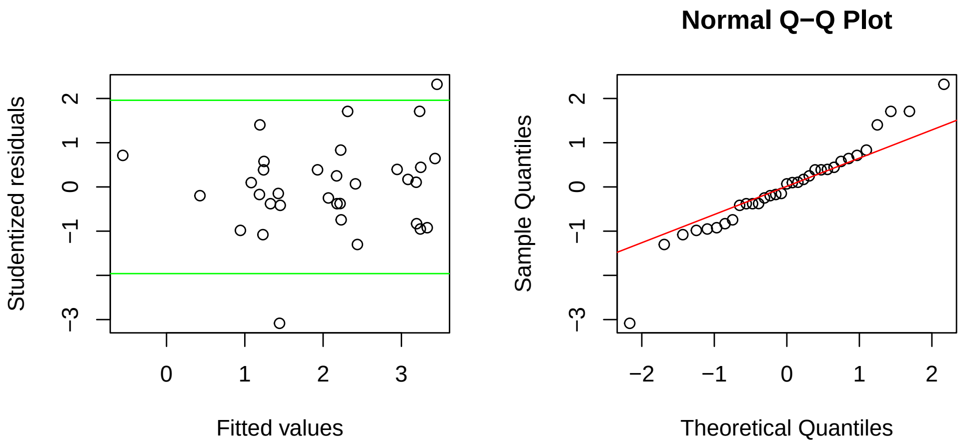


```
plot.lmer.influence(model_uncertainty, perf.wos)

## boundary (singular) fit:  see help('isSingular')
```

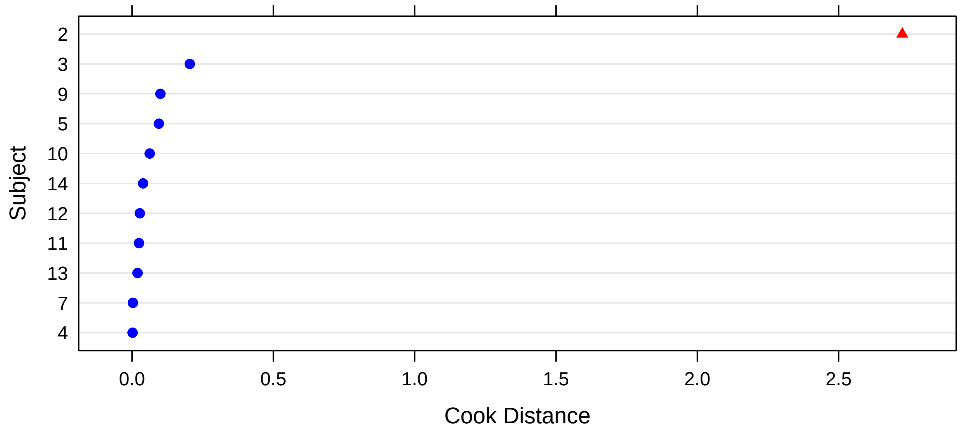


```
perf.wos = perf.wos[which(perf.wos$Sujet != 2),]
model_uncertainty = lmer(dprime ~ Uncertainty + (1|Sujet), data=perf.wos)

## boundary (singular) fit:  see help('isSingular')

plot.lmer.diagnostics(model_uncertainty, perf.wos)
```

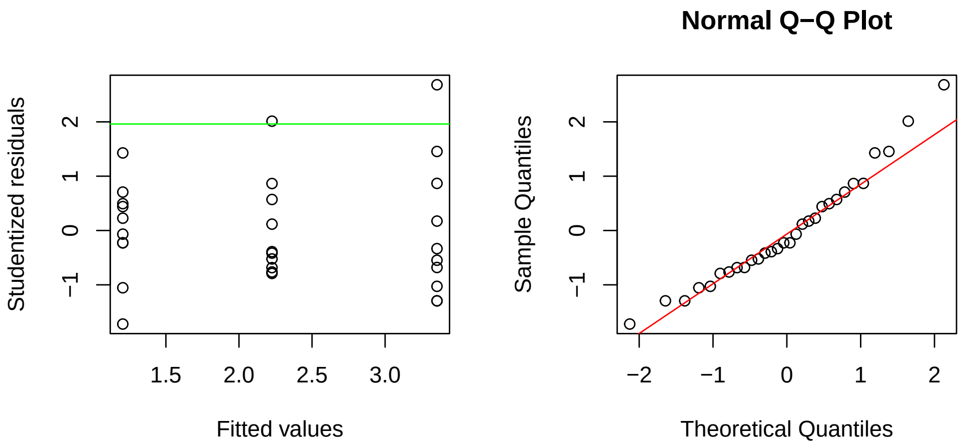


```
plot.lmer.influence(model_uncertainty, perf.wos)

## boundary (singular) fit:  see help('isSingular')
## boundary (singular) fit:  see help('isSingular')
## boundary (singular) fit:  see help('isSingular')
## boundary (singular) fit:  see help('isSingular')
## boundary (singular) fit:  see help('isSingular')
## boundary (singular) fit:  see help('isSingular')
## boundary (singular) fit:  see help('isSingular')
## boundary (singular) fit:  see help('isSingular')
## boundary (singular) fit:  see help('isSingular')
## boundary (singular) fit:  see help('isSingular')
```

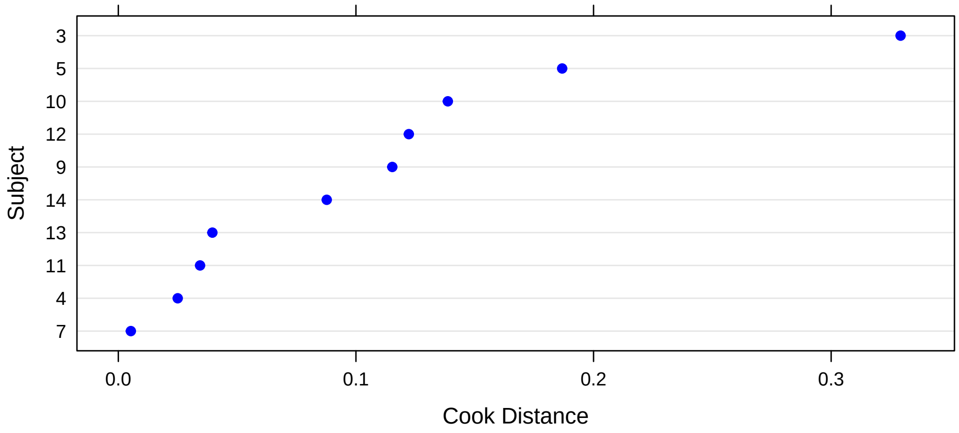


```
summary(model_uncertainty)

## Linear mixed model fit by REML. t-tests use Satterthwaite's method [
## lmerModLmerTest]
## Formula: dprime ~ Uncertainty + (1 | Sujet)
##    Data: perf.wos
##
## REML criterion at convergence: 33
##
## Scaled residuals:
##    Min     1Q Median     3Q    Max
## -1.633 -0.648 -0.215  0.523  2.545
##
## Random effects:
##  Groups   Name        Variance Std.Dev.
##  Sujet    (Intercept) 1.96e-21 4.43e-11
##  Residual             1.56e-01 3.95e-01
## Number of obs: 30, groups:  Sujet, 10
##
## Fixed effects:
##                 Estimate Std. Error     df t value Pr(>|t|)
## (Intercept)       3.355      0.125 27.000   26.87  < 2e-16 ***
## Uncertainty115   -1.129      0.177 27.000   -6.39  7.6e-07 ***
## Uncertainty463   -2.150      0.177 27.000  -12.17  1.8e-12 ***
## ---
## Signif. codes:  0 '***' 0.001 '**' 0.01 '*' 0.05 '.' 0.1 ' ' 1
##
## Correlation of Fixed Effects:
##             (Intr) Unc115
## Uncrtnty115 -0.707
## Uncrtnty463 -0.707  0.500
## optimizer (nloptwrap) convergence code: 0 (OK)
## boundary (singular) fit: see help('isSingular')
```

```
anova(model_uncertainty)

## Type III Analysis of Variance Table with Satterthwaite's method
##             Sum Sq Mean Sq NumDF DenDF F value  Pr(>F)
```

```
## Uncertainty   23.1    11.6     2    27    74.2 1.1e-11 ***
## ---
## Signif. codes:  0 '***' 0.001 '**' 0.01 '*' 0.05 '.' 0.1 ' ' 1
```

```
bwplot(dprime~Uncertainty, data=perf.wos)
```

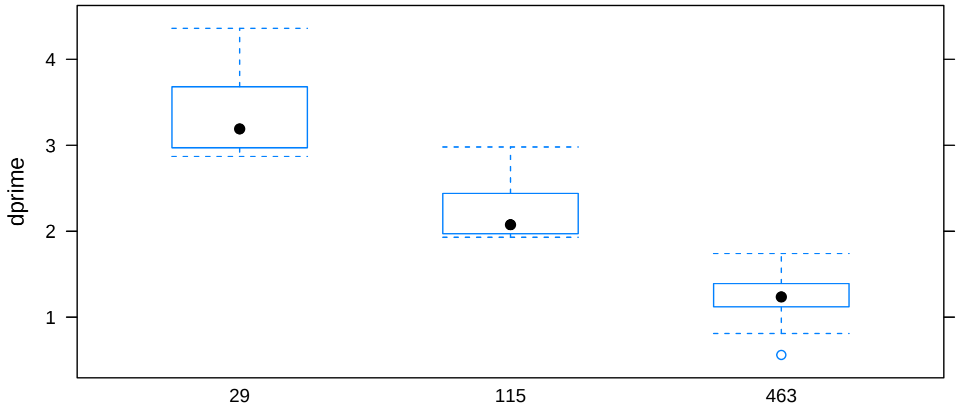


```
# boxplot(dprime~Uncertainty, data=perf.wos, ylim=c(-0.5, 4.5))
```

```
emmip(model_uncertainty, ~ Uncertainty, engine='lattice')
```

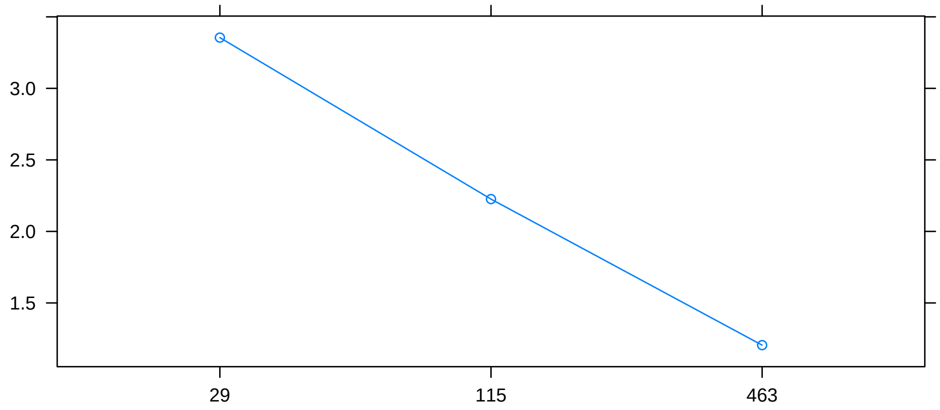


```
emm <- emmeans(model_uncertainty, specs=pairwise~Uncertainty, type="response")
summary(emm$emmeans, infer=TRUE)

##  Uncertainty emmean    SE df lower.CL upper.CL t.ratio p.value
##  29             3.4 0.125 27     3.10      3.6  26.900  <.0001
##  115            2.2 0.125 27     1.97      2.5  17.800  <.0001
##  463            1.2 0.125 27     0.95      1.5   9.600  <.0001
##
## Degrees-of-freedom method: kenward-roger
## Confidence level used: 0.95
```

```
pwpm(emm$emmeans)

##         29    115    463
## 29  [3.36] <.0001 <.0001
## 115   1.13 [2.23] <.0001
## 463   2.15   1.02 [1.20]
##
## Row and column labels: Uncertainty
## Upper triangle: P values   adjust = "tukey"
## Diagonal: [Estimates] (emmean)   type = "response"
## Lower triangle: Comparisons (estimate)   earlier vs. later

pwpp(emm$emmeans)
```

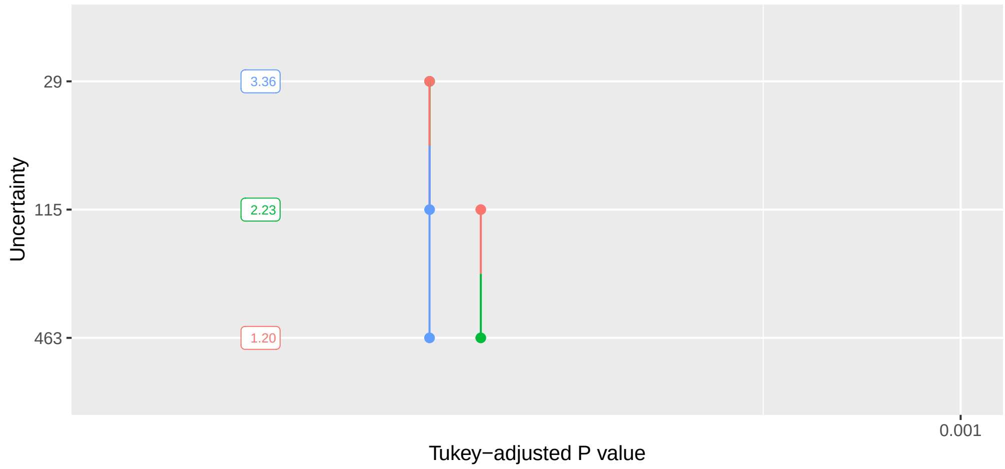


```
cld(emm$emmeans)

##  Uncertainty emmean    SE df lower.CL upper.CL .group
##  463            1.2 0.125 27     0.95      1.5  1
##  115            2.2 0.125 27     1.97      2.5   2
##  29             3.4 0.125 27     3.10      3.6    3
##
## Degrees-of-freedom method: kenward-roger
## Confidence level used: 0.95
## P value adjustment: tukey method for comparing a family of 3 estimates
## significance level used: alpha = 0.05
## NOTE: If two or more means share the same grouping letter,
##       then we cannot show them to be different.
##       But we also did not show them to be the same.
```

4 Reaction times

For comparison with d' results:

```
boxplot(RT~Uncertainty, data=rt.wos,
        main="Experiment II", xlab="Uncertainty (nats)")
```

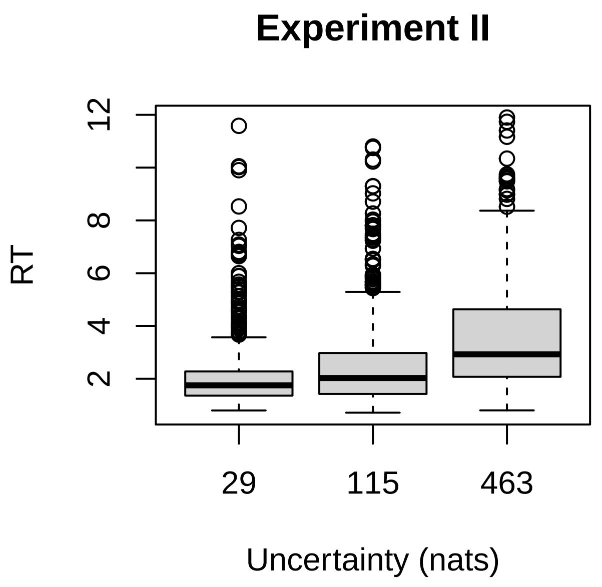


```
bwp1 <- bwplot(RT ~ Similarity | Uncertainty, data=rt.wos, layout=c(3,1))
bwp2 <- bwplot(RT ~ T.Rate | Uncertainty, data=rt.wos, layout=c(3,1))
grid.arrange(bwp1, bwp2, ncol=1, nrow=2)
```

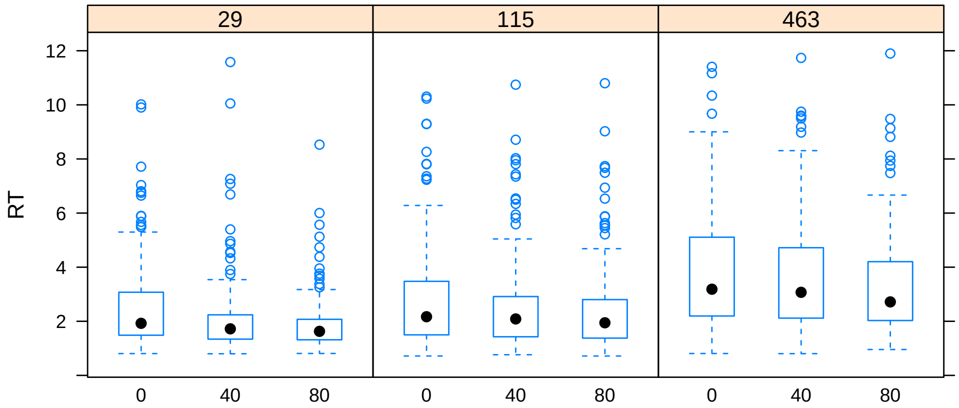

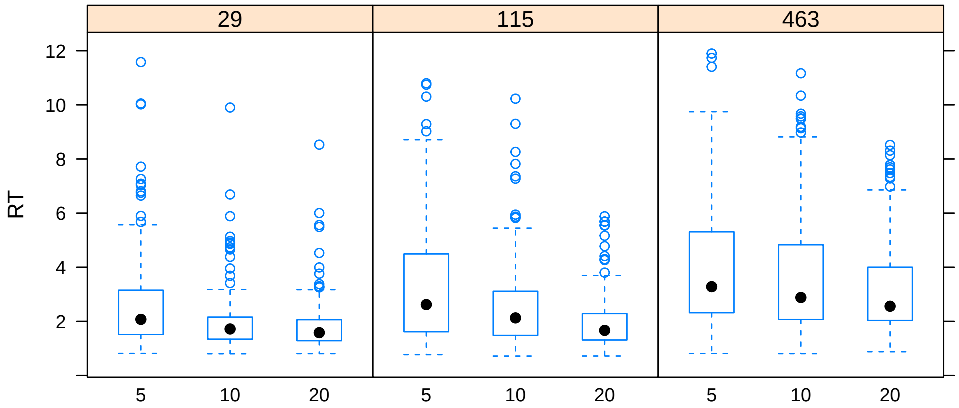


```
bwp1 <- bwplot(RT ~ Uncertainty | Similarity, data=rt.wos, layout=c(3,1))
bwp2 <- bwplot(RT ~ T.Rate | Similarity, data=rt.wos, layout=c(3,1))
grid.arrange(bwp1, bwp2, ncol=1, nrow=2)
```

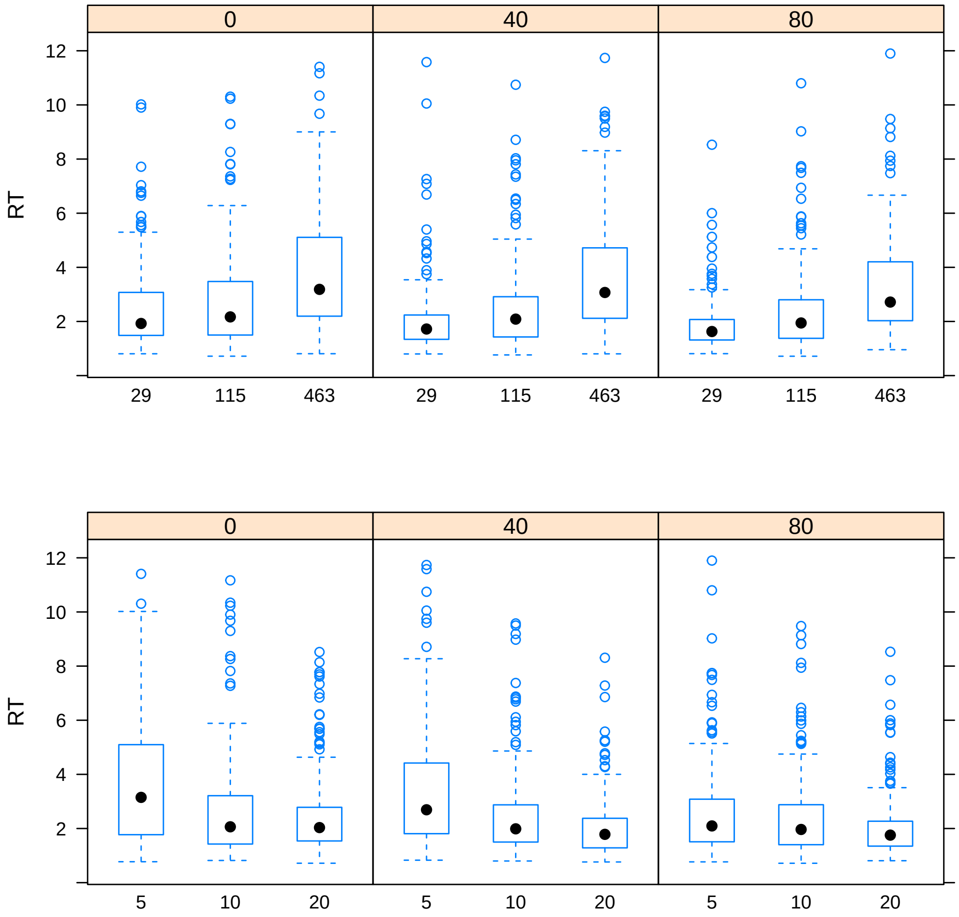


```
bwp1 <- bwplot(RT ~ Similarity | T.Rate, data=rt.wos, layout=c(3,1))
bwp2 <- bwplot(RT ~ Uncertainty | T.Rate, data=rt.wos, layout=c(3,1))
grid.arrange(bwp1, bwp2, ncol=1, nrow=2)
```

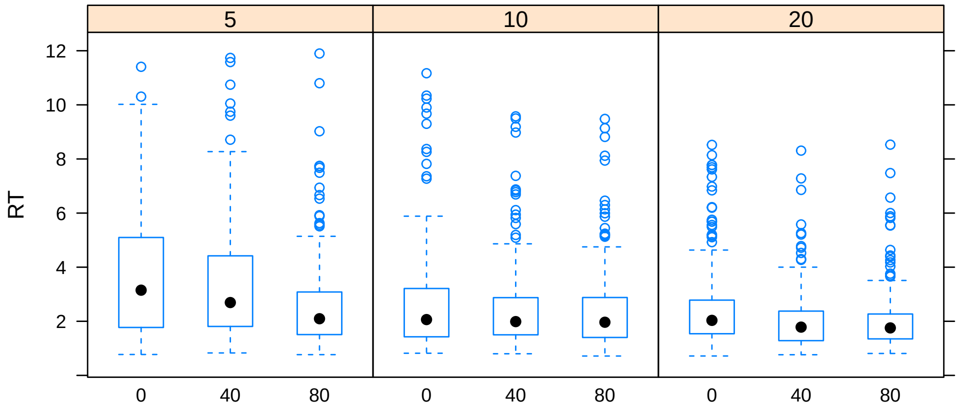

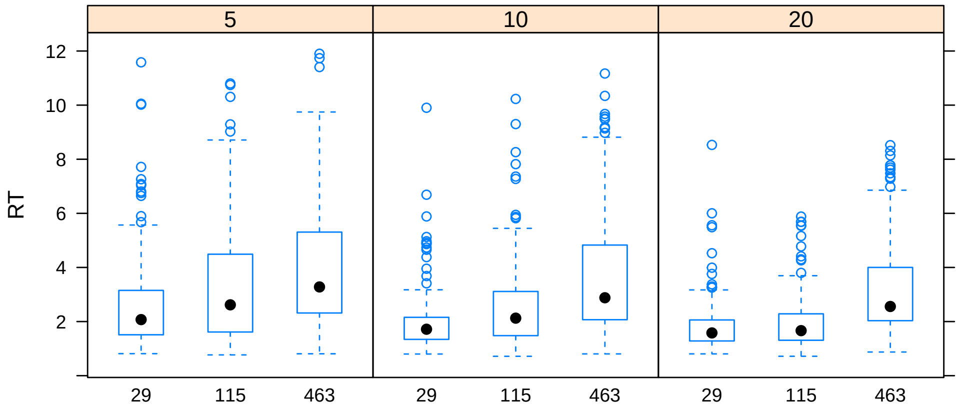

4.1 Time-to-event analysis

Creation of the survival object

```
# Selectioning reaction time for Hits from a cut of 700 ms
tte.wos = rbind(tte.wos[which(tte.wos$RT > 0.7 & tte.wos$Hits == 1),],
                tte.wos[which(tte.wos$Miss == 1),])
summary(tte.wos)

##      Sujet          Bloc        Stim               Hits             FA
##  3      :135   Min.   :2   Length:1742        Min.   :0.00   Min.   :0
##  4      :135   1st Qu.:3   Class :character   1st Qu.:1.00   1st Qu.:0
##  5      :135   Median :4   Mode  :character   Median :1.00   Median :0
##  7      :135   Mean   :4                      Mean   :0.81   Mean   :0
##  9      :135   3rd Qu.:5                      3rd Qu.:1.00   3rd Qu.:0
##  10     :135   Max.   :6                      Max.   :1.00   Max.   :0
##  (Other):932
##       Miss            RC          RT        Similarity     m_ppo
##  Min.   :0.00   Min.   :0   Min.   : 0.0   0 :579     Min.   : 4
##  1st Qu.:0.00   1st Qu.:0   1st Qu.: 1.1   40:582     1st Qu.: 4
##  Median :0.00   Median :0   Median : 1.8   80:581     Median :16
##  Mean   :0.19   Mean   :0   Mean   : 2.1              Mean   :28
##  3rd Qu.:0.00   3rd Qu.:0   3rd Qu.: 2.7              3rd Qu.:64
```

```
## Max.   :1.00   Max.   :0   Max.   :11.9            Max.   :64
##
##      m_td           m_iti            t_pi       T.Rate       t_td
## Min.   :0.02   Min.   :1500   Min.   : 489   5 :575   Min.   :0.02
## 1st Qu.:0.02   1st Qu.:1500   1st Qu.: 699   10:592   1st Qu.:0.02
## Median :0.06   Median :1500   Median :1430   20:575   Median :0.02
## Mean   :0.06   Mean   :1500   Mean   :1435            Mean   :0.02
## 3rd Qu.:0.10   3rd Qu.:1500   3rd Qu.:2045            3rd Qu.:0.02
## Max.   :0.10   Max.   :1500   Max.   :2924            Max.   :0.02
##
##   m_density    Uncertainty
## Min.   : 1.0   29 :577
## 1st Qu.: 1.0   115:583
## Median : 5.0   463:582
## Mean   : 8.1
## 3rd Qu.:18.0
## Max.   :19.0
##
```

```
# ADD THIS!
tte.wos$RT[which(tte.wos$Miss == 1)] <- 12

survie = Surv(tte.wos$RT, tte.wos$Hits)
model.wf <-  coxph(survie ~ Uncertainty * Similarity * T.Rate
      + frailty(Sujet, distribution='gaussian'), data=tte.wos)
plot.cox.diagnostics(model.wf, tte.wos)
```

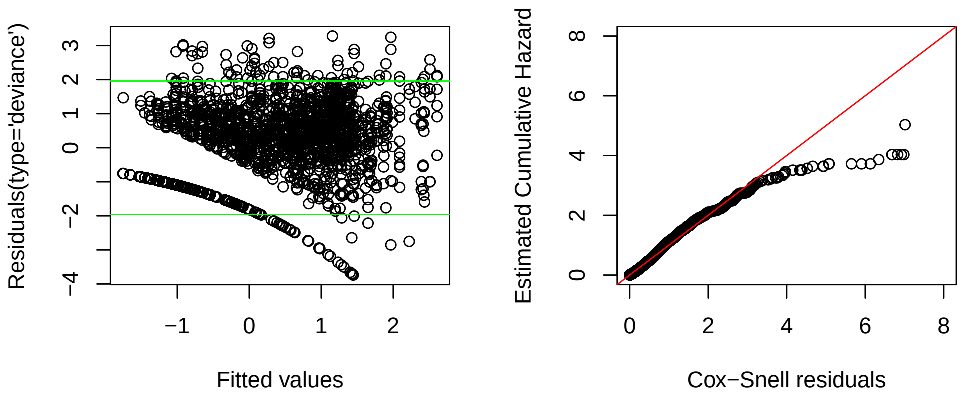


```
plot.cox.influence(model.wf, tte.wos)
```

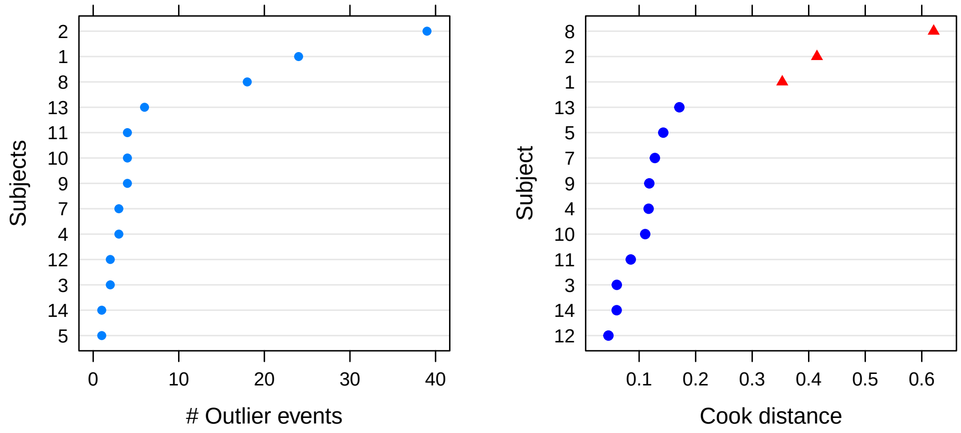


```
tte.wos = tte.wos[which(tte.wos$Sujet != 8),]
survie = Surv(tte.wos$RT, tte.wos$Hits)
model.wf <-  coxph(survie ~ Uncertainty * Similarity * T.Rate
      + frailty(Sujet, distribution='gaussian'), data=tte.wos)
plot.cox.diagnostics(model.wf, tte.wos)
```

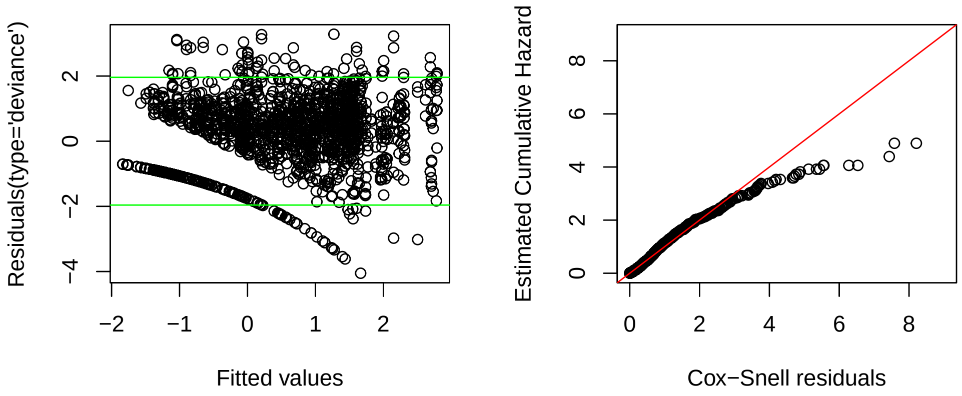


```
plot.cox.influence(model.wf, tte.wos)
```

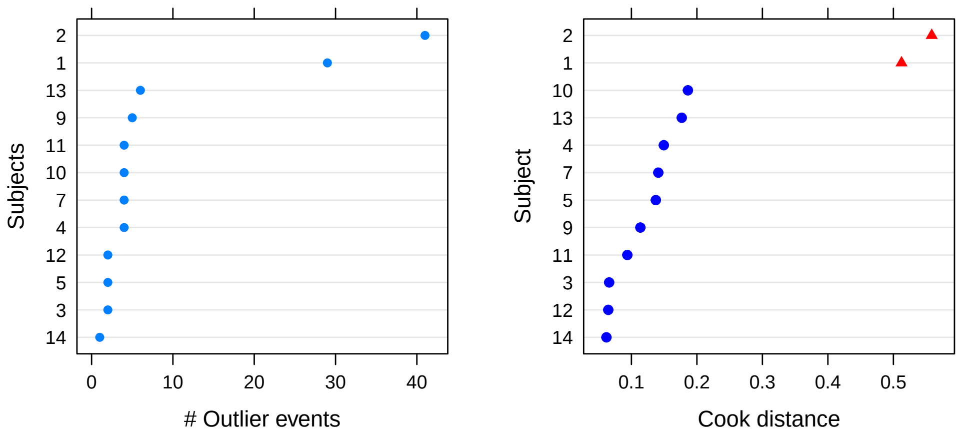


```
tte.wos = tte.wos[which(tte.wos$Sujet != 2),]
survie = Surv(tte.wos$RT, tte.wos$Hits)
model.wf <-  coxph(survie ~ Uncertainty * Similarity * T.Rate
      + frailty(Sujet, distribution='gaussian'), data=tte.wos)
plot.cox.diagnostics(model.wf, tte.wos)
```

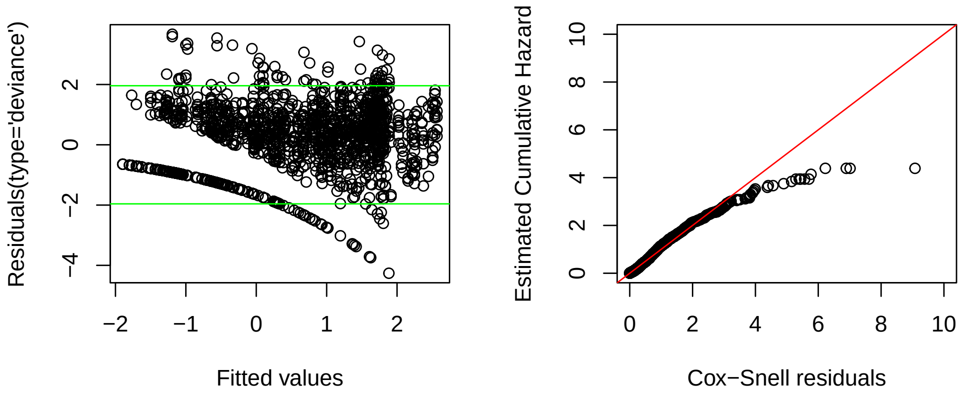


```
plot.cox.influence(model.wf, tte.wos)
```

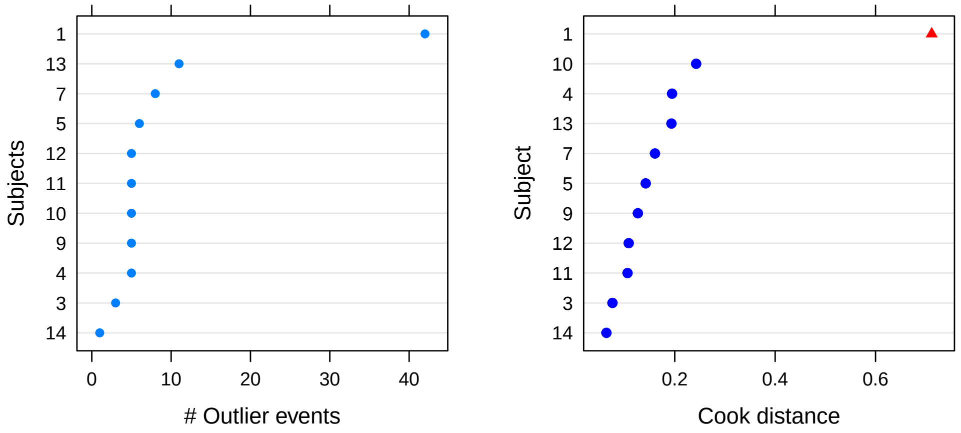


```
tte.wos = tte.wos[which(tte.wos$Sujet != 1),]
survie = Surv(tte.wos$RT, tte.wos$Hits)
model.wf <-  coxph(survie ~ Uncertainty * Similarity * T.Rate
      + frailty(Sujet, distribution='gaussian'), data=tte.wos)
plot.cox.diagnostics(model.wf, tte.wos)
```

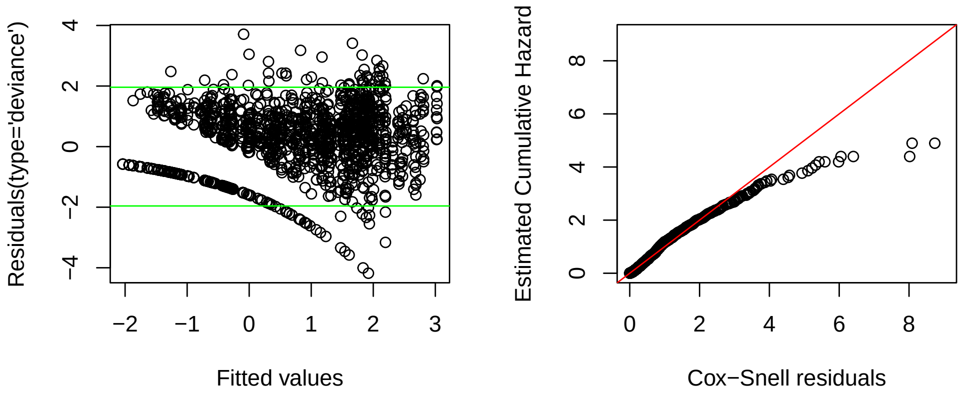


```
plot.cox.influence(model.wf, tte.wos)
```

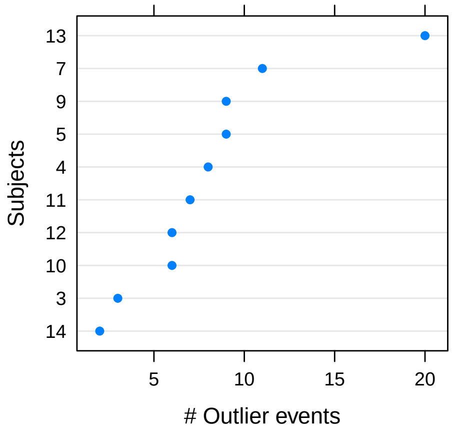

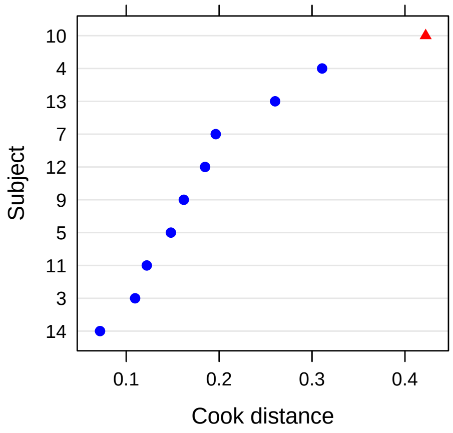

The total number of events (hits) is: 1059 and the average number of event per subject is 105.9 and the average number of event per modality is 39.22.

```
summary(model.wf)

## Call:
## coxph(formula = survie ~ Uncertainty * Similarity * T.Rate +
##     frailty(Sujet, distribution = "gaussian"), data = tte.wos)
##
##   n= 1350, number of events= 1059
##
##                           coef    se(coef) se2   Chisq  DF   p
## Uncertainty115            -1.1938 0.292    0.292  16.66 1.00 4.5e-05
## Uncertainty463            -1.3599 0.322    0.322  17.84 1.00 2.4e-05
## Similarity40               1.2853 0.218    0.218  34.60 1.00 4.0e-09
## Similarity80               1.8252 0.225    0.225  65.94 1.00 4.6e-16
## T.Rate10                   1.5819 0.219    0.219  52.02 1.00 5.5e-13
## T.Rate20                   1.8364 0.220    0.220  69.55 1.00 7.5e-17
## frailty(Sujet, distributi                        158.26 9.39 3.0e-29
## Uncertainty115:Similarity  0.2977 0.361    0.361   0.68 1.00 4.1e-01
## Uncertainty463:Similarity -1.0031 0.418    0.418   5.76 1.00 1.6e-02
## Uncertainty115:Similarity  0.1786 0.359    0.359   0.25 1.00 6.2e-01
## Uncertainty463:Similarity -1.5600 0.425    0.425  13.48 1.00 2.4e-04
## Uncertainty115:T.Rate10   -0.3765 0.366    0.366   1.06 1.00 3.0e-01
## Uncertainty463:T.Rate10   -1.4791 0.433    0.433  11.65 1.00 6.4e-04
## Uncertainty115:T.Rate20    0.5445 0.358    0.358   2.31 1.00 1.3e-01
## Uncertainty463:T.Rate20   -1.0879 0.405    0.405   7.22 1.00 7.2e-03
## Similarity40:T.Rate10     -0.8723 0.297    0.297   8.62 1.00 3.3e-03
## Similarity80:T.Rate10     -1.4532 0.302    0.302  23.19 1.00 1.5e-06
## Similarity40:T.Rate20     -0.9178 0.298    0.298   9.46 1.00 2.1e-03
## Similarity80:T.Rate20     -1.4516 0.302    0.302  23.10 1.00 1.5e-06
## Uncertainty115:Similarity  0.3098 0.468    0.468   0.44 1.00 5.1e-01
## Uncertainty463:Similarity  1.5345 0.558    0.558   7.56 1.00 6.0e-03
## Uncertainty115:Similarity  1.1515 0.468    0.468   6.06 1.00 1.4e-02
## Uncertainty463:Similarity  2.1513 0.561    0.561  14.69 1.00 1.3e-04
## Uncertainty115:Similarity  0.0846 0.463    0.463   0.03 1.00 8.6e-01
## Uncertainty463:Similarity  1.6656 0.529    0.529   9.92 1.00 1.6e-03
## Uncertainty115:Similarity  0.2489 0.461    0.461   0.29 1.00 5.9e-01
## Uncertainty463:Similarity  2.4954 0.534    0.534  21.86 1.00 2.9e-06
##
```

```
##                              exp(coef) exp(-coef) lower .95 upper .95
## Uncertainty115                  0.303     3.2996    0.1708     0.538
## Uncertainty463                  0.257     3.8959    0.1366     0.482
## Similarity40                    3.616     0.2766    2.3562     5.548
## Similarity80                    6.204     0.1612    3.9935     9.638
## T.Rate10                        4.864     0.2056    3.1647     7.477
## T.Rate20                        6.274     0.1594    4.0749     9.660
## Uncertainty115:Similarity       1.347     0.7425    0.6638     2.733
## Uncertainty463:Similarity       0.367     2.7268    0.1616     0.832
## Uncertainty115:Similarity       1.196     0.8364    0.5920     2.415
## Uncertainty463:Similarity       0.210     4.7588    0.0914     0.483
## Uncertainty115:T.Rate10         0.686     1.4572    0.3349     1.406
## Uncertainty463:T.Rate10         0.228     4.3891    0.0974     0.533
## Uncertainty115:T.Rate20         1.724     0.5801    0.8543     3.478
## Uncertainty463:T.Rate20         0.337     2.9680    0.1524     0.745
## Similarity40:T.Rate10           0.418     2.3924    0.2335     0.748
## Similarity80:T.Rate10           0.234     4.2766    0.1294     0.422
## Similarity40:T.Rate20           0.399     2.5037    0.2225     0.717
## Similarity80:T.Rate20           0.234     4.2699    0.1296     0.423
## Uncertainty115:Similarity       1.363     0.7336    0.5448     3.411
## Uncertainty463:Similarity       4.639     0.2156    1.5538    13.850
## Uncertainty115:Similarity       3.163     0.3161    1.2644     7.913
## Uncertainty463:Similarity       8.596     0.1163    2.8606    25.831
## Uncertainty115:Similarity       1.088     0.9189    0.4391     2.697
## Uncertainty463:Similarity       5.289     0.1891    1.8763    14.909
## Uncertainty115:Similarity       1.283     0.7797    0.5198     3.164
## Uncertainty463:Similarity      12.126     0.0825    4.2599    34.519
##
## Iterations: 5 outer, 16 Newton-Raphson
##      Variance of random effect= 0.17
## Degrees of freedom for terms= 2.0 2.0 2.0 9.4 4.0 4.0 4.0 8.0
## Concordance= 0.798  (se = 0.006 )
## Likelihood ratio test= 1085  on 35.4 df,   p=<2e-16
```

```
anova(model.wf)

## Analysis of Deviance Table
##  Cox model: response is survie
## Terms added sequentially (first to last)
##
##                                               loglik Chisq   Df Pr(>|Chi|)
## NULL                                           -7021
## Uncertainty                                    -6807 428.5  2.0    < 2e-16 ***
## Similarity                                     -6722 170.2  2.0    < 2e-16 ***
## T.Rate                                         -6598 249.1  2.0    < 2e-16 ***
## frailty(Sujet, distribution = "gaussian")      -6591  13.5  1.0    0.00023 ***
## Uncertainty:Similarity                         -6584  13.0  4.0    0.01110 *
## Uncertainty:T.Rate                             -6578  12.1  4.0    0.01659 *
## Similarity:T.Rate                              -6568  19.8  4.0    0.00055 ***
## Uncertainty:Similarity:T.Rate                  -6479 178.7 16.4    < 2e-16 ***
## ---
## Signif. codes:  0 '***' 0.001 '**' 0.01 '*' 0.05 '.' 0.1 ' ' 1
```

Survival: post-hocs with emmeans

```
emmip(model.wf, Uncertainty ~ T.Rate | Similarity, engine='lattice')
```

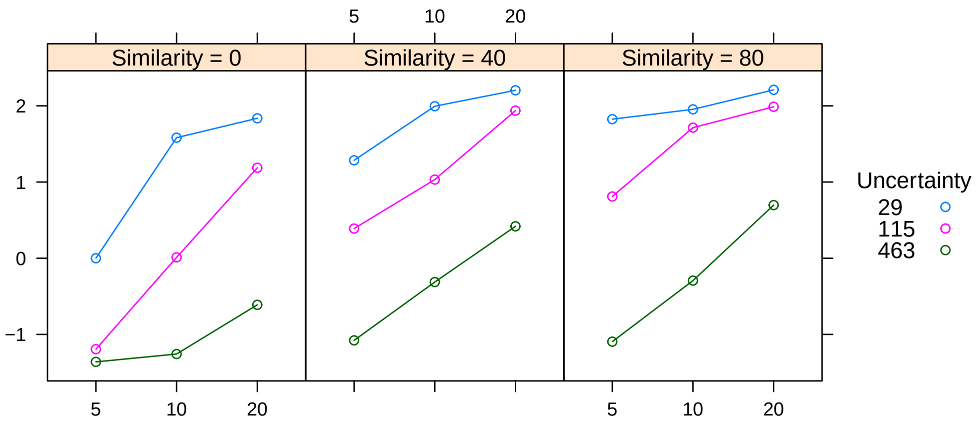


```
emmip(model.wf, T.Rate ~ Uncertainty | Similarity, engine='lattice')
```

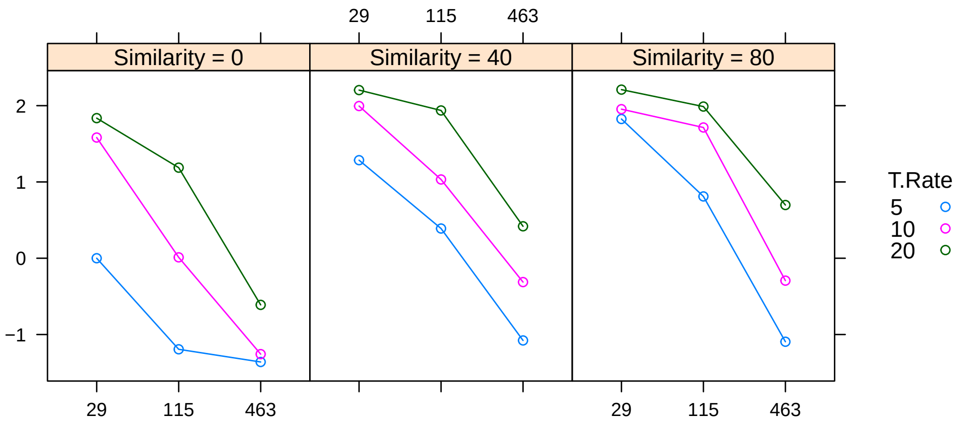


```
emmip(model.wf, Similarity ~ T.Rate | Uncertainty, engine='lattice')
```

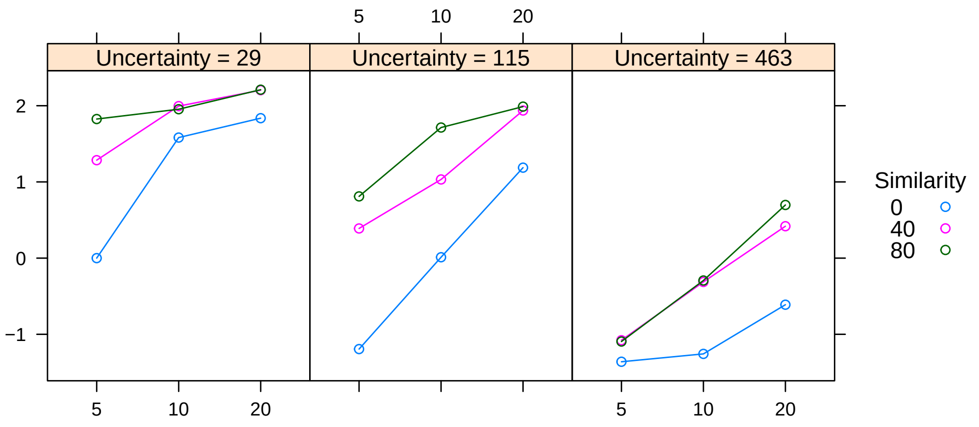

```
emmip(model.wf, T.Rate ~ Similarity | Uncertainty, engine='lattice')
```

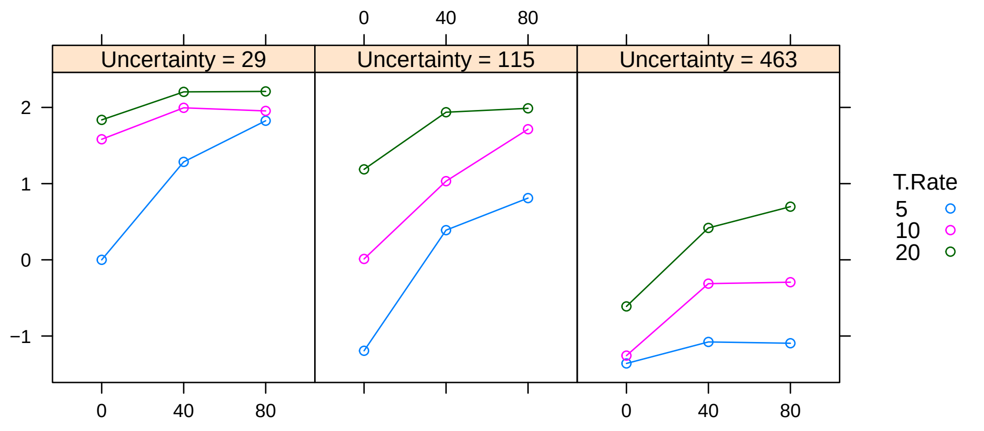


```
emmip(model.wf, Similarity ~ Uncertainty | T.Rate, engine='lattice')
```

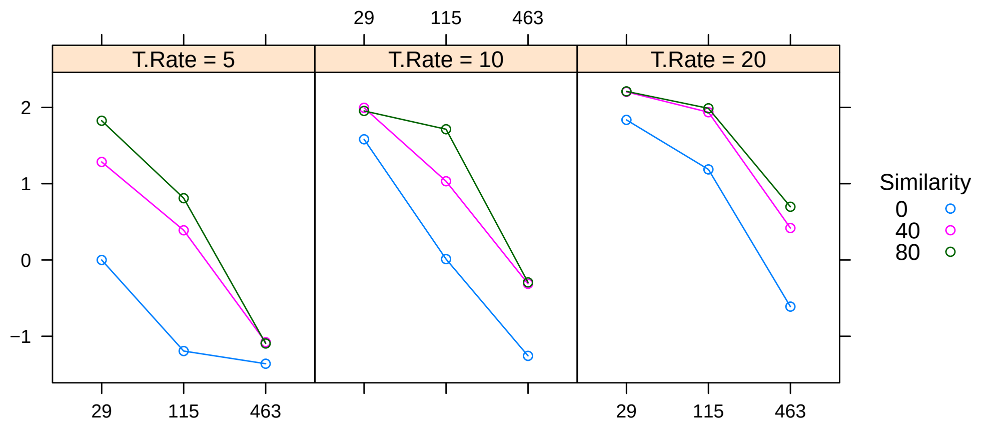


```
emmip(model.wf, Uncertainty ~ Similarity | T.Rate, engine='lattice')
```

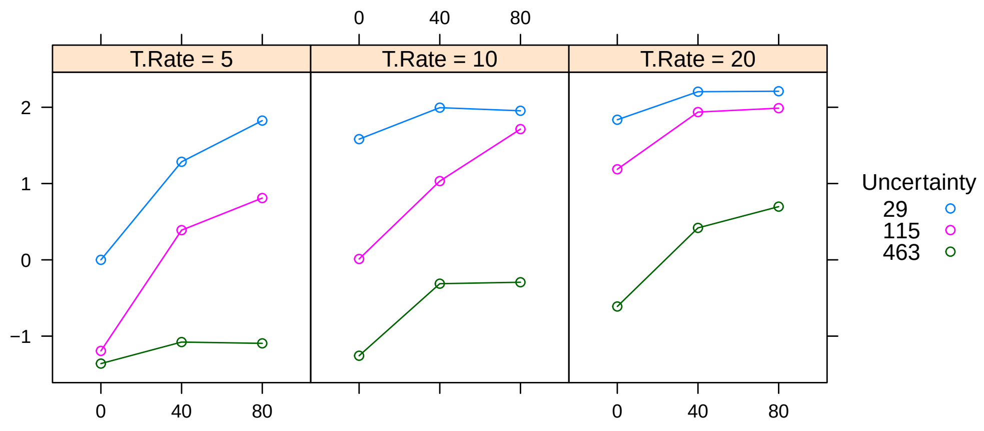

```
emm <- emmeans(model.wf, specs=pairwise~Similarity:T.Rate:Uncertainty)
pwpm(emm$emmeans)
```

```
##              0 5 29   40 5 29   80 5 29   0 10 29  40 10 29  80 10 29   0 20 29
## 0 5 29    [ 0.0000]    <.0001    <.0001    <.0001    <.0001    <.0001    <.0001
## 40 5 29    -1.28528 [ 1.2853]    0.6601    0.9997    0.0937    0.1699    0.5833
## 80 5 29    -1.82519  -0.53991 [ 1.8252]    1.0000    1.0000    1.0000    1.0000
## 0 10 29    -1.58193  -0.29665   0.24326 [ 1.5819]    0.9604    0.9890    1.0000
## 40 10 29   -1.99491  -0.70963  -0.16972  -0.41298 [ 1.9949]    1.0000    1.0000
## 80 10 29   -1.95395  -0.66866  -0.12876  -0.37202   0.04096 [ 1.9539]    1.0000
## 0 20 29    -1.83644  -0.55116  -0.01125  -0.25451   0.15847   0.11751 [ 1.8364]
## 40 20 29   -2.20396  -0.91868  -0.37878  -0.62204  -0.20906  -0.25002  -0.36752
## 80 20 29   -2.21004  -0.92476  -0.38486  -0.62812  -0.21514  -0.25610  -0.37361
## 0 5 115     1.19380   2.47909   3.01899   2.77573   3.18871   3.14775   3.03024
## 40 5 115   -0.38922   0.89607   1.43597   1.19271   1.60569   1.56473   1.44722
## 80 5 115   -0.81000   0.47528   1.01518   0.77192   1.18490   1.14394   1.02643
## 0 10 115   -0.01160   1.27368   1.81359   1.57033   1.98331   1.94234   1.82484
## 40 10 115  -1.03216   0.25312   0.79302   0.54976   0.96274   0.92178   0.80428
## 80 10 115  -1.71379  -0.42851   0.11140  -0.13186   0.28112   0.24016   0.12265
## 0 20 115   -1.18718   0.09810   0.63801   0.39475   0.80773   0.76677   0.64926
## 40 20 115  -1.93701  -0.65172  -0.11182  -0.35508   0.05790   0.01694  -0.10057
## 80 20 115  -1.98826  -0.70298  -0.16308  -0.40634   0.00664  -0.03432  -0.15182
## 0 5 463     1.35994   2.64522   3.18512   2.94186   3.35484   3.31388   3.19637
## 40 5 463    1.07779   2.36307   2.90298   2.65972   3.07270   3.03174   2.91423
## 80 5 463    1.09475   2.38003   2.91994   2.67668   3.08966   3.04870   2.93119
## 0 10 463    1.25713   2.54241   3.08231   2.83905   3.25203   3.21107   3.09357
## 40 10 463   0.31278   1.59806   2.13797   1.89471   2.30769   2.26673   2.14922
## 80 10 463   0.29380   1.57908   2.11899   1.87573   2.28871   2.24775   2.13024
## 0 20 463    0.61140   1.89668   2.43659   2.19333   2.60631   2.56535   2.44784
## 40 20 463  -0.41860   0.86668   1.40659   1.16333   1.57631   1.53535   1.41784
## 80 20 463  -0.69758   0.58771   1.12761   0.88435   1.29733   1.25637   1.13886
##            40 20 29  80 20 29   0 5 115  40 5 115  80 5 115  0 10 115 40 10 115
## 0 5 29       <.0001    <.0001    0.0122    0.9952    0.0426    1.0000    0.0009
## 40 5 29      0.0019    0.0018    <.0001    0.0074    0.8302    <.0001    1.0000
## 80 5 29      0.9886    0.9880    <.0001    <.0001    0.0004    <.0001    0.0415
## 0 10 29      0.3133    0.2790    <.0001    <.0001    0.0332    <.0001    0.5984
## 40 10 29     1.0000    1.0000    <.0001    <.0001    <.0001    <.0001    0.0009
## 80 10 29     1.0000    1.0000    <.0001    <.0001    <.0001    <.0001    0.0022
## 0 20 29      0.9919    0.9884    <.0001    <.0001    0.0002    <.0001    0.0267
## 40 20 29  [ 2.2040]    1.0000    <.0001    <.0001    <.0001    <.0001    <.0001
## 80 20 29   -0.00608 [ 2.2100]    <.0001    <.0001    <.0001    <.0001    <.0001
## 0 5 115     3.39777   3.40385 [-1.1938]    <.0001    <.0001    0.0110    <.0001
## 40 5 115    1.81475   1.82083  -1.58302 [ 0.3892]    0.9663    0.9972    0.3319
## 80 5 115    1.39396   1.40004  -2.00381  -0.42079 [ 0.8100]    0.0557    1.0000
## 0 10 115    2.19236   2.19844  -1.20541   0.37761   0.79840 [ 0.0116]    0.0013
## 40 10 115   1.17180   1.17788  -2.22597  -0.64295  -0.22216  -1.02056 [ 1.0322]
## 80 10 115   0.49017   0.49625  -2.90759  -1.32457  -0.90379  -1.70219  -0.68163
## 0 20 115    1.01678   1.02287  -2.38098  -0.79796  -0.37717  -1.17558  -0.15502
## 40 20 115   0.26696   0.27304  -3.13081  -1.54779  -1.12700  -1.92540  -0.90484
## 80 20 115   0.21570   0.22178  -3.18207  -1.59905  -1.17826  -1.97666  -0.95610
## 0 5 463     3.56390   3.56998   0.16613   1.74915   2.16994   1.37154   2.39210
## 40 5 463    3.28176   3.28784  -0.11601   1.46701   1.88780   1.08939   2.10996
## 80 5 463    3.29871   3.30479  -0.09906   1.48396   1.90475   1.10635   2.12691
## 0 10 463    3.46109   3.46717   0.06332   1.64634   2.06713   1.26873   2.28929
## 40 10 463   2.51675   2.52283  -0.88102   0.70200   1.12279   0.32438   1.34495
## 80 10 463   2.49776   2.50385  -0.90000   0.68302   1.10381   0.30540   1.32596
## 0 20 463    2.81536   2.82144  -0.58241   1.00061   1.42140   0.62300   1.64356
```

```
## 40 20 463   1.78536   1.79144  -1.61241  -0.02938   0.39140  -0.40700   0.61356
## 80 20 463   1.50639   1.51247  -1.89138  -0.30836   0.11243  -0.68597   0.33459
##           80 10 115  0 20 115 40 20 115 80 20 115   0 5 463  40 5 463  80 5 463
## 0 5 29       <.0001    <.0001    <.0001    <.0001    0.0069    0.0253    0.0258
## 40 5 29      0.9418    1.0000    0.1973    0.1054    <.0001    <.0001    <.0001
## 80 5 29      1.0000    0.3341    1.0000    1.0000    <.0001    <.0001    <.0001
## 0 10 29      1.0000    0.9806    0.9943    0.9696    <.0001    <.0001    <.0001
## 40 10 29     0.9999    0.0241    1.0000    1.0000    <.0001    <.0001    <.0001
## 80 10 29     1.0000    0.0478    1.0000    1.0000    <.0001    <.0001    <.0001
## 0 20 29      1.0000    0.2570    1.0000    1.0000    <.0001    <.0001    <.0001
## 40 20 29     0.7975    0.0003    0.9999    1.0000    <.0001    <.0001    <.0001
## 80 20 29     0.7839    0.0003    0.9999    1.0000    <.0001    <.0001    <.0001
## 0 5 115      <.0001    <.0001    <.0001    <.0001    1.0000    1.0000    1.0000
## 40 5 115     <.0001    0.0481    <.0001    <.0001    <.0001    <.0001    <.0001
## 80 5 115     0.0027    0.9884    <.0001    <.0001    <.0001    <.0001    <.0001
## 0 10 115     <.0001    <.0001    <.0001    <.0001    0.0062    0.0228    0.0235
## 40 10 115    0.1647    1.0000    0.0030    0.0011    <.0001    <.0001    <.0001
## 80 10 115 [ 1.7138]    0.7076    1.0000    0.9999    <.0001    <.0001    <.0001
## 0 20 115    0.52661 [ 1.1872]    0.0559    0.0273    <.0001    <.0001    <.0001
## 40 20 115  -0.22322  -0.74983 [ 1.9370]    1.0000    <.0001    <.0001    <.0001
## 80 20 115  -0.27447  -0.80108  -0.05126 [ 1.9883]    <.0001    <.0001    <.0001
## 0 5 463     3.07373   2.54711   3.29694   3.34820 [-1.3599]    1.0000    1.0000
## 40 5 463    2.79158   2.26497   3.01480   3.06606  -0.28214 [-1.0778]    1.0000
## 80 5 463    2.80854   2.28193   3.03175   3.08301  -0.26519   0.01696 [-1.0947]
## 0 10 463    2.97092   2.44431   3.19413   3.24539  -0.10281   0.17933   0.16238
## 40 10 463   2.02657   1.49996   2.24979   2.30105  -1.04715  -0.76501  -0.78197
## 80 10 463   2.00759   1.48098   2.23081   2.28207  -1.06613  -0.78399  -0.80095
## 0 20 463    2.32519   1.79858   2.54840   2.59966  -0.74854  -0.46639  -0.48335
## 40 20 463   1.29519   0.76858   1.51841   1.56966  -1.77854  -1.49639  -1.51335
## 80 20 463   1.01621   0.48960   1.23943   1.29069  -2.05751  -1.77537  -1.79232
##            0 10 463 40 10 463 80 10 463  0 20 463 40 20 463 80 20 463
## 0 5 29       0.0073    1.0000    1.0000    0.8042    0.9868    0.2607
## 40 5 29      <.0001    <.0001    <.0001    <.0001    0.0140    0.4932
## 80 5 29      <.0001    <.0001    <.0001    <.0001    <.0001    <.0001
## 0 10 29      <.0001    <.0001    <.0001    <.0001    <.0001    0.0074
## 40 10 29     <.0001    <.0001    <.0001    <.0001    <.0001    <.0001
## 80 10 29     <.0001    <.0001    <.0001    <.0001    <.0001    <.0001
## 0 20 29      <.0001    <.0001    <.0001    <.0001    <.0001    <.0001
## 40 20 29     <.0001    <.0001    <.0001    <.0001    <.0001    <.0001
## 80 20 29     <.0001    <.0001    <.0001    <.0001    <.0001    <.0001
## 0 5 115      1.0000    0.4005    0.3420    0.9858    <.0001    <.0001
## 40 5 115     <.0001    0.3733    0.4173    0.0171    1.0000    0.9998
## 80 5 115     <.0001    0.0003    0.0004    <.0001    0.9876    1.0000
## 0 10 115     0.0066    0.9999    1.0000    0.7811    0.9919    0.3052
## 40 10 115    <.0001    <.0001    <.0001    <.0001    0.4510    0.9988
## 80 10 115    <.0001    <.0001    <.0001    <.0001    <.0001    0.0005
## 0 20 115     <.0001    <.0001    <.0001    <.0001    0.0790    0.8610
## 40 20 115    <.0001    <.0001    <.0001    <.0001    <.0001    <.0001
## 80 20 115    <.0001    <.0001    <.0001    <.0001    <.0001    <.0001
## 0 5 463      1.0000    0.2343    0.1954    0.9066    <.0001    <.0001
## 40 5 463     1.0000    0.6047    0.5354    0.9989    <.0001    <.0001
## 80 5 463     1.0000    0.5920    0.5230    0.9985    <.0001    <.0001
## 0 10 463  [-1.2571]    0.2958    0.2463    0.9602    <.0001    <.0001
## 40 10 463  -0.94434 [-0.3128]    1.0000    1.0000    0.2931    0.0056
## 80 10 463  -0.96332  -0.01898 [-0.2938]    1.0000    0.3278    0.0068
## 0 20 463   -0.64573   0.29862   0.31760 [-0.6114]    0.0114    <.0001
```

```
## 40 20 463  -1.67573  -0.73138  -0.71240  -1.03000 [ 0.4186]    1.0000
## 80 20 463  -1.95470  -1.01036  -0.99138  -1.30897  -0.27898 [ 0.6976]
##
## Row and column labels: Similarity:T.Rate:Uncertainty
## Upper triangle: P values   adjust = "tukey"
## Diagonal: [Estimates] (emmean)
## Lower triangle: Comparisons (estimate)   earlier vs. later

pwpp(emm$emmeans)
```

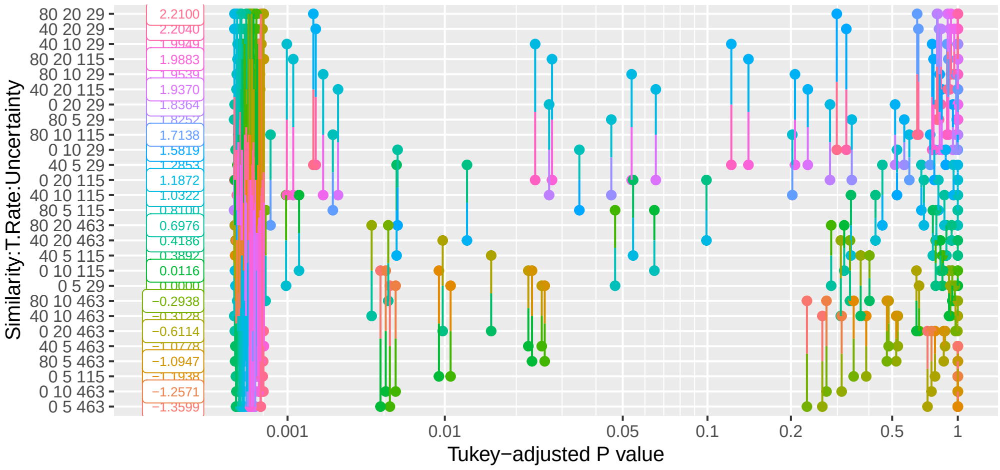


```
# Table 5
emm.cld <- cld(emm$emmeans)
plot(emm.cld)
```

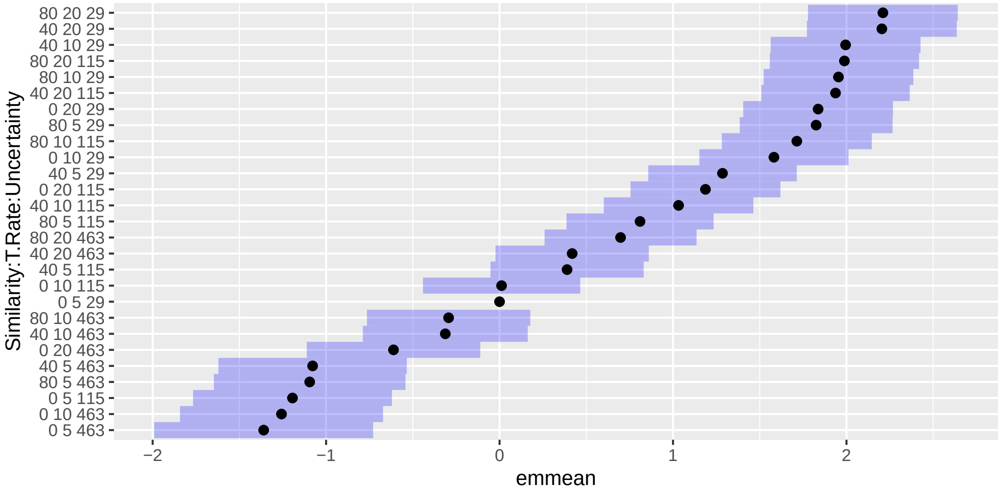


```
emm.cld

##  Similarity T.Rate Uncertainty emmean   SE  df asymp.LCL asymp.UCL
##  0          5      463          -1.36 0.32 Inf     -1.99     -0.73
##  0          10     463          -1.26 0.30 Inf     -1.84     -0.67
##  0          5      115          -1.19 0.29 Inf     -1.77     -0.62
##  80         5      463          -1.09 0.28 Inf     -1.65     -0.54
##  40         5      463          -1.08 0.28 Inf     -1.62     -0.53
```

```
##  0          20     463           -0.61 0.26 Inf     -1.11     -0.11
##  40         10     463           -0.31 0.24 Inf     -0.79      0.16
##  80         10     463           -0.29 0.24 Inf     -0.76      0.18
##  0          5      29             0.00 0.00 Inf      0.00      0.00
##  0          10     115            0.01 0.23 Inf     -0.44      0.46
##  40         5      115            0.39 0.23 Inf     -0.05      0.83
##  40         20     463            0.42 0.23 Inf     -0.02      0.86
##  80         20     463            0.70 0.22 Inf      0.26      1.14
##  80         5      115            0.81 0.22 Inf      0.39      1.23
##  40         10     115            1.03 0.22 Inf      0.60      1.46
##  0          20     115            1.19 0.22 Inf      0.76      1.62
##  40         5      29             1.29 0.22 Inf      0.86      1.71
##  0          10     29             1.58 0.22 Inf      1.15      2.01
##  80         10     115            1.71 0.22 Inf      1.28      2.15
##  80         5      29             1.83 0.22 Inf      1.38      2.27
##  0          20     29             1.84 0.22 Inf      1.40      2.27
##  40         20     115            1.94 0.22 Inf      1.51      2.36
##  80         10     29             1.95 0.22 Inf      1.52      2.39
##  80         20     115            1.99 0.22 Inf      1.56      2.42
##  40         10     29             1.99 0.22 Inf      1.56      2.43
##  40         20     29             2.20 0.22 Inf      1.77      2.64
##  80         20     29             2.21 0.22 Inf      1.78      2.64
##  .group
##   1
##   1
##   1
##   1
##   1
##   12
##   123
##   123
##    234
##    2345
##     3456
##     34567
##      45678
##       5678
##        6789
##         7890
##          890A
##           90AB
##           90AB
##            0AB
##            0AB
##            0AB
##             AB
##             AB
##             AB
##              B
##              B
##
## Results are given on the log (not the response) scale.
## Confidence level used: 0.95
## P value adjustment: tukey method for comparing a family of 27 estimates
## significance level used: alpha = 0.05
## NOTE: If two or more means share the same grouping letter,
```

```
##       then we cannot show them to be different.
##       But we also did not show them to be the same.
```
