# Supplementary material for: Investigating the influence of masker and target properties on the dynamics of perceptual awareness under informational masking
Source: PLoS One. 2023 Mar 16;18(3):e0282885. doi: 10.1371/journal.pone.0282885 (PMC10019711; doi:10.1371/journal.pone.0282885)

Reproducible Report for PONE-D-22-05102 - Investigating the influence of masker and target properties on the dynamics of perceptual awareness under informational masking
Experiment III

A. Veyrié and L. Pezard

Thursday 29th September, 2022

Contents

1 Loading data **1**
1.1 Performance data . . . 1
1.2 Reaction times . . . 2

2 Qualitative description of performance indices and reaction times **3**
2.1 Performance indices (d') distributions . . . 3
2.2 Reaction times distributions . . . 5
2.3 Conclusion . . . 5

3 Detection performance (d') **6**

4 Reaction times **10**
4.1 Time-to-event analysis . . . 12

A Check mixed model for d' **21**

```
library(nlme)
library(emmeans)
library(xtable)
library(lme4)
library(lmerTest)
library(survival)
library(influence.ME)
library(multcomp)
library(lattice)
source('../utils.R')
```

1 Loading data

1.1 Performance data

Loading dataframe

```
perf.all = read.table("../data/performance_Exp_III.csv",
                          header=TRUE, sep=',', dec=".",
                          fileEncoding="utf-8")
# Changing variable in categoric factor
perf.all$Uncertainty = factor(perf.all$Uncertainty)
summary(perf.all)

##      Sujet         Exp            Uncertainty     nbhits          nbmiss
##  Min.   : 1   Length:117         84     :13   Min.   : 3.0   Min.   : 0.0
##  1st Qu.: 4   Class :character   110    :13   1st Qu.:11.0   1st Qu.: 0.0
##  Median : 7   Mode  :character   123    :13   Median :14.0   Median : 1.0
##  Mean   : 7                      169    :13   Mean   :13.1   Mean   : 1.7
##  3rd Qu.:10                      221    :13   3rd Qu.:15.0   3rd Qu.: 2.0
##  Max.   :13                      246    :13   Max.   :18.0   Max.   :14.0
##                                  (Other):39
##       nbfa          nbrc          hitsrate        fasrate         dprime
##  Min.   : 0   Min.   : 0.0   Min.   :0.23   Min.   :0.03   Min.   :-0.5
##  1st Qu.: 0   1st Qu.: 4.0   1st Qu.:0.83   1st Qu.:0.08   1st Qu.: 1.7
##  Median : 0   Median : 5.0   Median :0.91   Median :0.08   Median : 2.5
##  Mean   : 1   Mean   : 6.2   Mean   :0.86   Mean   :0.19   Mean   : 2.3
##  3rd Qu.: 1   3rd Qu.: 5.0   3rd Qu.:0.97   3rd Qu.:0.25   3rd Qu.: 3.2
##  Max.   :15   Max.   :15.0   Max.   :0.97   Max.   :0.97   Max.   : 3.8
##
```

1.2 Reaction times

Loading the dataframe

```
rt.all = read.table("../data/data_Exp_III.csv",
                    header=TRUE, sep=',', dec=".", fileEncoding="utf-8")
# Changing reaction times from msec to sec
rt.all$RT = rt.all$RT/1000
# Removing the first bloc (because of learning)
rt.all = rt.all[which(rt.all$Bloc != 1),]
```

```
# Transforming variable into categoric factor
rt.all$T.Rate = factor(rt.all$T.Rate)
rt.all$Sujet = factor(rt.all$Sujet)
rt.all$Uncertainty = factor(rt.all$Uncertainty)
summary(rt.all)

##      Sujet            Bloc         Stim                 Hits            FA
##  1      : 200   Min.   :2   Length:2600        Min.   :0.0   Min.   :0.00
##  2      : 200   1st Qu.:3   Class :character   1st Qu.:0.0   1st Qu.:0.00
##  3      : 200   Median :4   Mode  :character   Median :1.0   Median :0.00
##  4      : 200   Mean   :4                      Mean   :0.6   Mean   :0.04
##  5      : 200   3rd Qu.:5                      3rd Qu.:1.0   3rd Qu.:0.00
##  6      : 200   Max.   :6                      Max.   :1.0   Max.   :1.00
##  (Other):1400
##       Miss             RC              RT             Diss         m_ppo
##  Min.   :0.00   Min.   :0.00   Min.   : 0.0   Min.   :40    Min.   :16
##  1st Qu.:0.00   1st Qu.:0.00   1st Qu.: 0.0   1st Qu.:40    1st Qu.:16
##  Median :0.00   Median :0.00   Median : 1.7   Median :40    Median :32
##  Mean   :0.08   Mean   :0.28   Mean   : 2.1   Mean   :40    Mean   :38
##  3rd Qu.:0.00   3rd Qu.:1.00   3rd Qu.: 2.8   3rd Qu.:40    3rd Qu.:64
```

```
##  Max.   :1.00   Max.   :1.00   Max.   :12.0   Max.   :40    Max.   :64
##                                               NA's   :845
##       m_td            m_iti            t_pi       T.Rate          t_td
##  Min.   :0.020   Min.   : 300   Min.   : 489   1    :591   Min.   :0
##  1st Qu.:0.020   1st Qu.: 300   1st Qu.: 699   2    :582   1st Qu.:0
##  Median :0.020   Median :1100   Median :1000   5    :582   Median :0
##  Mean   :0.026   Mean   :1221   Mean   :1424   NA's:845    Mean   :0
##  3rd Qu.:0.020   3rd Qu.:2300   3rd Qu.:2045               3rd Qu.:0
##  Max.   :0.100   Max.   :2300   Max.   :2924               Max.   :0
##                                 NA's   :845                NA's   :845
##    m_density      Uncertainty
##  Min.   : 4.0   442    :384
##  1st Qu.: 6.0   110    :383
##  Median : 9.0   221    :265
##  Mean   :11.4   123    :264
##  3rd Qu.:17.0   492    :264
##  Max.   :23.0   169    :263
##                 (Other):777
```

2 Qualitative description of performance indices and reaction times

2.1 Performance indices (d') distributions

Histogram of the all dprime

```
hist(perf.all[,"dprime"],
     breaks=30, col=c("skyblue"), prob=TRUE, xlab="d'", main="")
lines(density(perf.all[,"dprime"]))
abline(v=mean(perf.all[,"dprime"]), col="green")
```

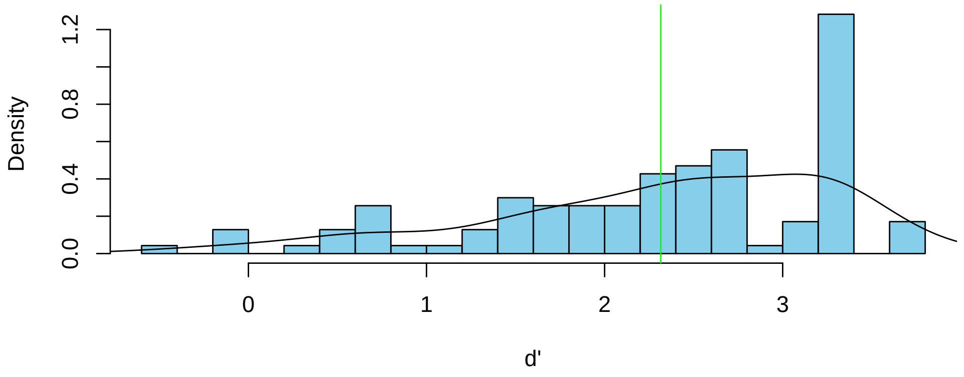

Boxplot of performance indices per subjects

```
par(mfrow=c(3,1))
boxplot(hitsrate ~ Sujet, col=c("skyblue"), data=perf.all,
        xlab="Subject", ylab="Hit's rate", main="")
abline(h=mean(perf.all[,"hitsrate"]), col="green")
boxplot(fasrate ~ Sujet, col=c("skyblue"), data=perf.all,
```

```
        xlab="Subject", ylab="False alarm's rate", main="")
abline(h=mean(perf.all[,"fasrate"]), col="green")
boxplot(dprime ~ Sujet,
        col=c("skyblue"), data=perf.all, xlab="Subject", ylab="d'", main="")
abline(h=mean(perf.all[,"dprime"]), col="green")
```

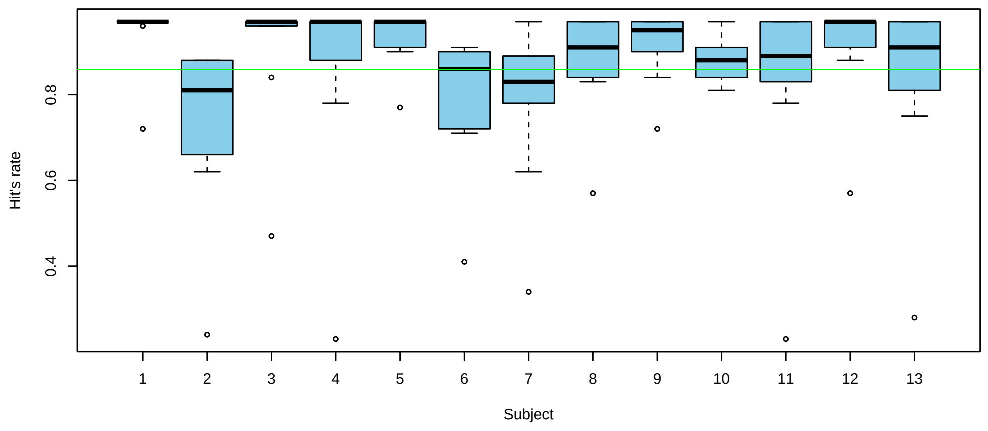

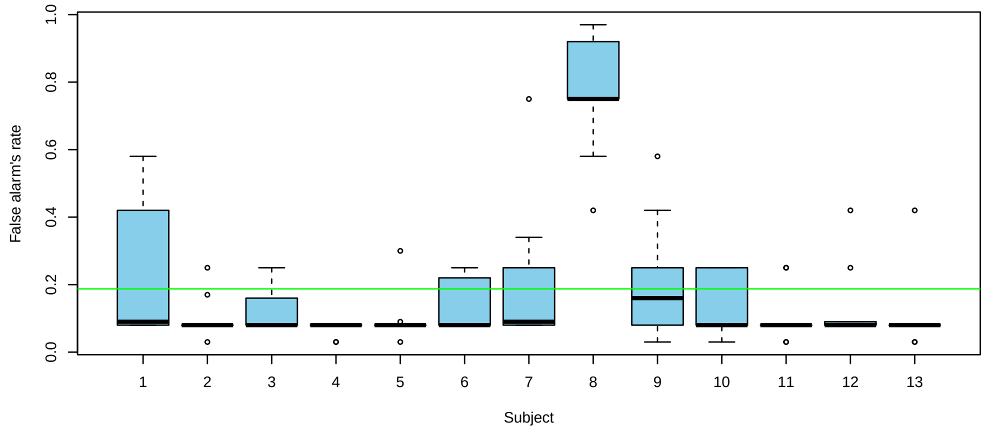

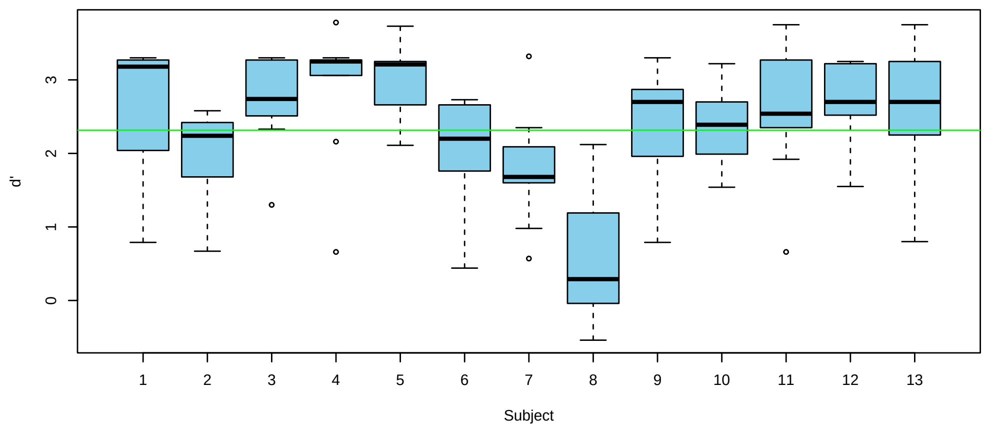

2.2 Reaction times distributions

Ploting the reaction times distribution and a gamma distribution

```
# Selectioning reaction time for Hits from a cut of 1100 ms
rt.hits = rt.all[which(rt.all$RT > 1.1 & rt.all$Hits == 1),]
#par(mfrow=c(1,2))
hist(rt.hits[,"RT"], breaks=50, col=c("skyblue"),
     prob=TRUE, xlab="TD (ms)", main="")
lines(density(rt.hits[,"RT"]))
abline(v=mean(rt.hits[,"RT"]), col="green")
```

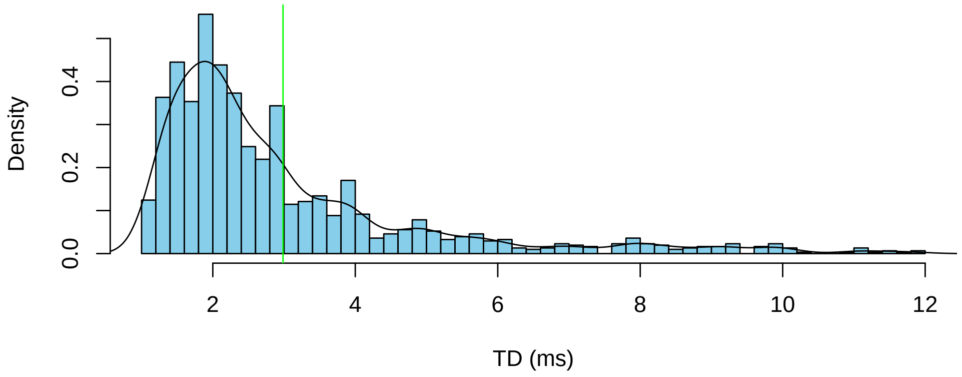

Boxplot of dprime values by subject

```
boxplot(RT ~ Sujet,
        col=c("skyblue"), data=rt.hits, xlab="Subject", ylab="DT", main="")
abline(h=mean(rt.hits[,"RT"]), col="green")
```

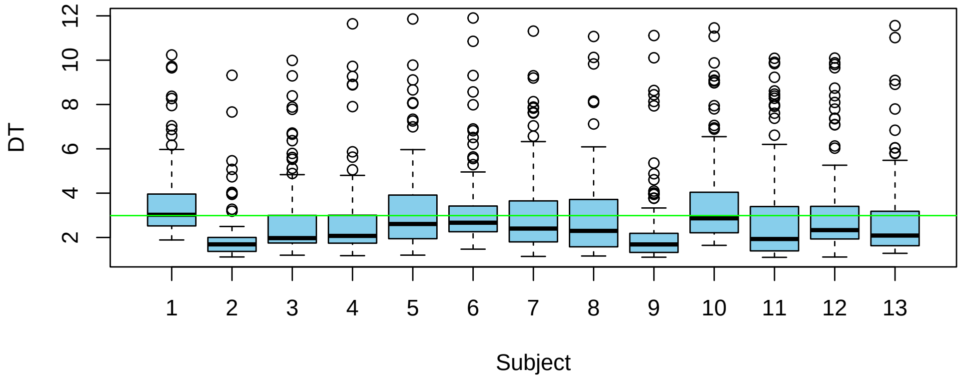

2.3 Conclusion

Removing the outliers subjects n°8 due to high alarm rate

```
perf.wos = perf.all[which(perf.all$Sujet != 8),]
rt.wos = rt.hits[which(rt.hits$Sujet != 8),]
tte.wos = rt.all[which(rt.all$Sujet != 8),]
```

N.B.: Subject n°8 is also the first removed subject as influencial subject for mixed effect model as shown in appendix A.

3 Detection performance (d')

```
bwplot(dprime~Uncertainty, data=perf.wos)
```

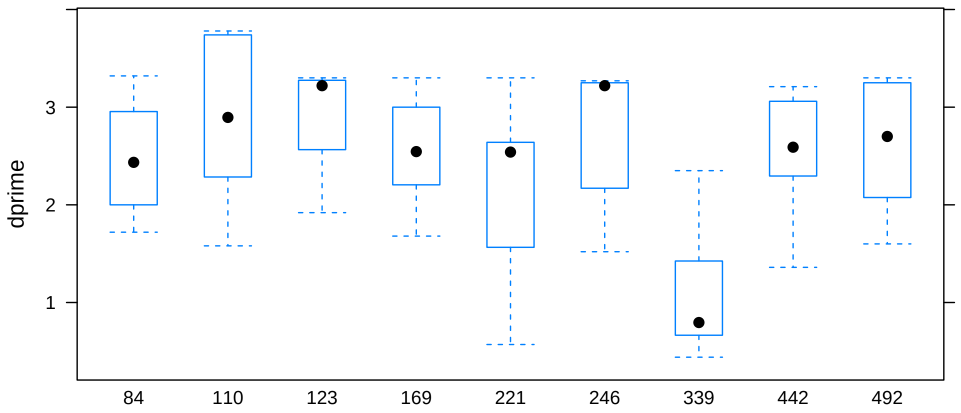


```
model_uncertainty = lmer(dprime ~ Uncertainty + (1|Sujet), data=perf.wos)
plot.lmer.diagnostics(model_uncertainty, perf.wos)
```

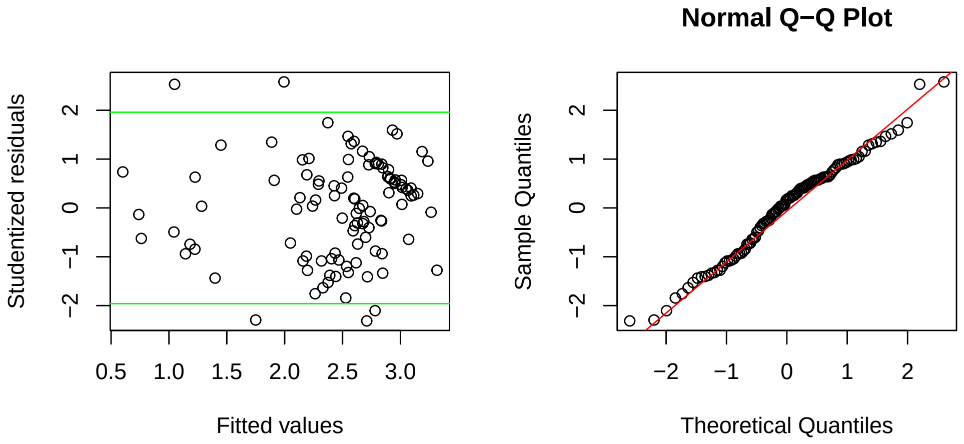


```
plot.lmer.influence(model_uncertainty, perf.wos)
```

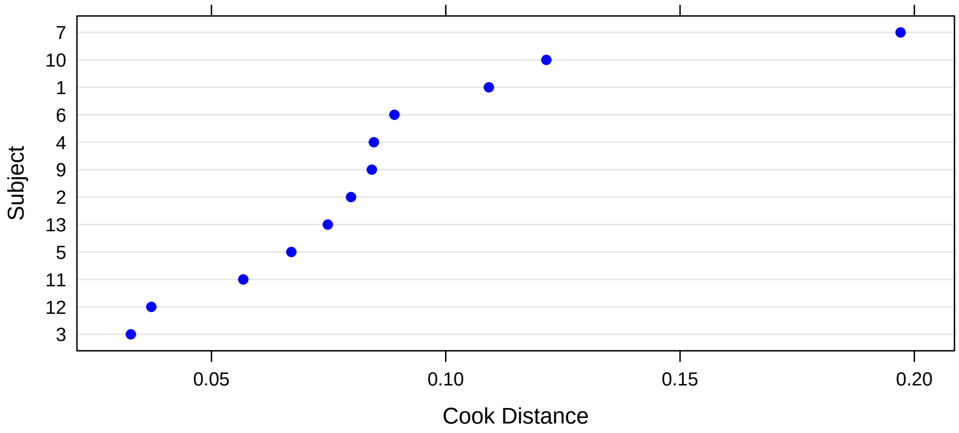

No subject has an oversized influence on the model fitting.

```
summary(model_uncertainty)

## Linear mixed model fit by REML. t-tests use Satterthwaite's method [
## lmerModLmerTest]
## Formula: dprime ~ Uncertainty + (1 | Sujet)
##    Data: perf.wos
##
## REML criterion at convergence: 203
##
## Scaled residuals:
##    Min     1Q Median     3Q    Max
## -2.123 -0.706  0.158  0.586  2.368
##
## Random effects:
##  Groups   Name        Variance Std.Dev.
##  Sujet    (Intercept) 0.0968   0.311
##  Residual             0.3138   0.560
## Number of obs: 108, groups:  Sujet, 12
##
## Fixed effects:
##                 Estimate Std. Error      df t value Pr(>|t|)
## (Intercept)       2.4842     0.1850 68.5241   13.43  < 2e-16 ***
## Uncertainty110    0.3958     0.2287 88.0000    1.73    0.087 .
## Uncertainty123    0.4742     0.2287 88.0000    2.07    0.041 *
## Uncertainty169    0.0558     0.2287 88.0000    0.24    0.808
## Uncertainty221   -0.2442     0.2287 88.0000   -1.07    0.289
## Uncertainty246    0.2733     0.2287 88.0000    1.20    0.235
## Uncertainty339   -1.3925     0.2287 88.0000   -6.09  2.9e-08 ***
## Uncertainty442    0.1092     0.2287 88.0000    0.48    0.634
## Uncertainty492    0.1642     0.2287 88.0000    0.72    0.475
## ---
## Signif. codes:  0 '***' 0.001 '**' 0.01 '*' 0.05 '.' 0.1 ' ' 1
##
## Correlation of Fixed Effects:
##             (Intr) Unc110 Unc123 Unc169 Unc221 Unc246 Unc339 Unc442
## Uncrtnty110 -0.618
## Uncrtnty123 -0.618  0.500
## Uncrtnty169 -0.618  0.500  0.500
```

```
## Uncrtnty221 -0.618  0.500  0.500  0.500
## Uncrtnty246 -0.618  0.500  0.500  0.500  0.500
## Uncrtnty339 -0.618  0.500  0.500  0.500  0.500  0.500
## Uncrtnty442 -0.618  0.500  0.500  0.500  0.500  0.500  0.500
## Uncrtnty492 -0.618  0.500  0.500  0.500  0.500  0.500  0.500  0.500

anova(model_uncertainty)

## Type III Analysis of Variance Table with Satterthwaite's method
##             Sum Sq Mean Sq NumDF DenDF F value  Pr(>F)
## Uncertainty   29.9    3.74     8    88    11.9 2.3e-11 ***
## ---
## Signif. codes:  0 '***' 0.001 '**' 0.01 '*' 0.05 '.' 0.1 ' ' 1
```

```
# Figure 2.C.
pdf("../../manuscript/figures/results/Exp-III/performance.pdf", width=7.5, height=3.5)
par(oma=c(0,0,0,0), mar=c(4,4,2,0)+0.1)
boxplot(dprime~Uncertainty, data=perf.wos, ylim=c(-0.5,4.5),
        main="Experiment III", xlab="Uncertainty (nats)", ylab="d'")
dev.off()
```

Performance (new parts) : post-hocs with emmeans

```
emmip(model_uncertainty, ~ Uncertainty, engine='lattice')
```

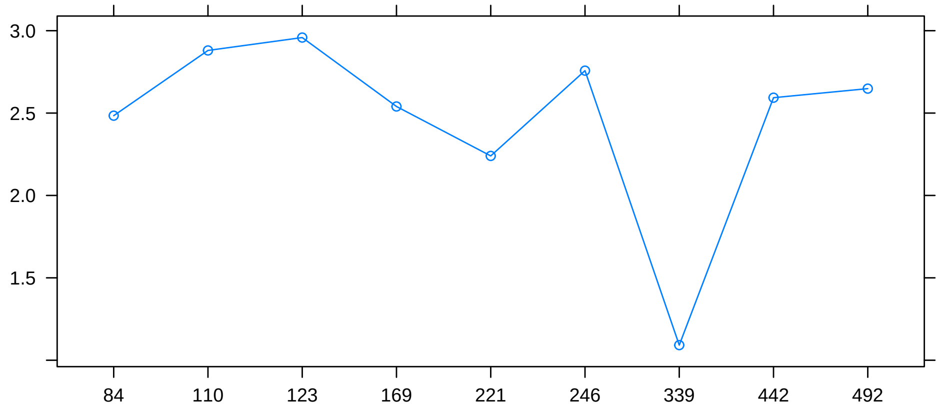


```
emm = emmeans(model_uncertainty, specs=pairwise~Uncertainty, type="response")
xt <- xtable(summary(emm$contrasts, infer=TRUE))
pxt <- print(xt, tabular.environment='longtable', floating=FALSE, print.result=FALSE)
#cat(paste("\\begin{landscape}", pxt, "\\end{landscape}", sep='\n'))
cat(pxt)
```

contrast	estimate	SE	df	lower.CL	upper.CL	t.ratio	p.value
Uncertainty84 - Uncertainty110	-0.3958	0.2287	88	-1.1231	0.3315	-1.731	0.7260
Uncertainty84 - Uncertainty123	-0.4742	0.2287	88	-1.2015	0.2531	-2.073	0.4978
Uncertainty84 - Uncertainty169	-0.0558	0.2287	88	-0.7831	0.6715	-0.244	1.0000
Uncertainty84 - Uncertainty221	0.2442	0.2287	88	-0.4831	0.9715	1.068	0.9773
Uncertainty84 - Uncertainty246	-0.2733	0.2287	88	-1.0006	0.4540	-1.195	0.9555

Uncertainty84 - Uncertainty339	1.3925	0.2287	88	0.6652	2.1198	6.089	<.0001
Uncertainty84 - Uncertainty442	-0.1092	0.2287	88	-0.8365	0.6181	-0.477	0.9999
Uncertainty84 - Uncertainty492	-0.1642	0.2287	88	-0.8915	0.5631	-0.718	0.9984
Uncertainty110 - Uncertainty123	-0.0783	0.2287	88	-0.8056	0.6490	-0.343	1.0000
Uncertainty110 - Uncertainty169	0.3400	0.2287	88	-0.3873	1.0673	1.487	0.8587
Uncertainty110 - Uncertainty221	0.6400	0.2287	88	-0.0873	1.3673	2.799	0.1306
Uncertainty110 - Uncertainty246	0.1225	0.2287	88	-0.6048	0.8498	0.536	0.9998
Uncertainty110 - Uncertainty339	1.7883	0.2287	88	1.0610	2.5156	7.820	<.0001
Uncertainty110 - Uncertainty442	0.2867	0.2287	88	-0.4406	1.0140	1.254	0.9417
Uncertainty110 - Uncertainty492	0.2317	0.2287	88	-0.4956	0.9590	1.013	0.9837
Uncertainty123 - Uncertainty169	0.4183	0.2287	88	-0.3090	1.1456	1.829	0.6628
Uncertainty123 - Uncertainty221	0.7183	0.2287	88	-0.0090	1.4456	3.141	0.0556
Uncertainty123 - Uncertainty246	0.2008	0.2287	88	-0.5265	0.9281	0.878	0.9936
Uncertainty123 - Uncertainty339	1.8667	0.2287	88	1.1394	2.5940	8.163	<.0001
Uncertainty123 - Uncertainty442	0.3650	0.2287	88	-0.3623	1.0923	1.596	0.8044
Uncertainty123 - Uncertainty492	0.3100	0.2287	88	-0.4173	1.0373	1.356	0.9110
Uncertainty169 - Uncertainty221	0.3000	0.2287	88	-0.4273	1.0273	1.312	0.9252
Uncertainty169 - Uncertainty246	-0.2175	0.2287	88	-0.9448	0.5098	-0.951	0.9891
Uncertainty169 - Uncertainty339	1.4483	0.2287	88	0.7210	2.1756	6.333	<.0001
Uncertainty169 - Uncertainty442	-0.0533	0.2287	88	-0.7806	0.6740	-0.233	1.0000
Uncertainty169 - Uncertainty492	-0.1083	0.2287	88	-0.8356	0.6190	-0.474	0.9999
Uncertainty221 - Uncertainty246	-0.5175	0.2287	88	-1.2448	0.2098	-2.263	0.3757
Uncertainty221 - Uncertainty339	1.1483	0.2287	88	0.4210	1.8756	5.021	0.0001
Uncertainty221 - Uncertainty442	-0.3533	0.2287	88	-1.0806	0.3740	-1.545	0.8309
Uncertainty221 - Uncertainty492	-0.4083	0.2287	88	-1.1356	0.3190	-1.786	0.6914
Uncertainty246 - Uncertainty339	1.6658	0.2287	88	0.9385	2.3931	7.284	<.0001
Uncertainty246 - Uncertainty442	0.1642	0.2287	88	-0.5631	0.8915	0.718	0.9984
Uncertainty246 - Uncertainty492	0.1092	0.2287	88	-0.6181	0.8365	0.477	0.9999
Uncertainty339 - Uncertainty442	-1.5017	0.2287	88	-2.2290	-0.7744	-6.567	<.0001
Uncertainty339 - Uncertainty492	-1.5567	0.2287	88	-2.2840	-0.8294	-6.807	<.0001
Uncertainty442 - Uncertainty492	-0.0550	0.2287	88	-0.7823	0.6723	-0.241	1.0000

Degrees-of-freedom method: kenward-roger
Confidence level used: 0.95
Conf-level adjustment: tukey method for comparing a family of 9 estimates
P value adjustment: tukey method for comparing a family of 9 estimates

```
# Temporary Table 5
emm.cld <- cld(emm$emmeans)
plot(emm.cld)
```

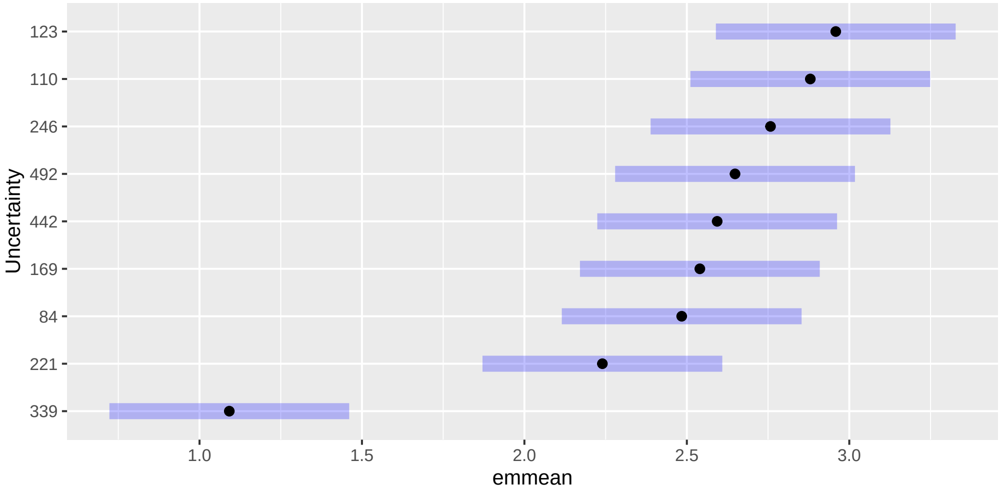

```
xtt <- xtable(emm.cld)
# Why it does not work!?
#print(xtt, file="../../manuscript/tmp-tables/Exp-II_cld.tex", print.result=FALSE)
#print(xtable(cld(emm£emmeans)), file="tt.tt")
```

```
pwpm(emm$emmeans)

##          84     110     123     169     221     246     339     442    492
## 84    [2.48]  0.7260  0.4978  1.0000  0.9773  0.9555  <.0001  0.9999 0.9984
## 110 -0.3958  [2.88]  1.0000  0.8587  0.1306  0.9998  <.0001  0.9417 0.9837
## 123 -0.4742 -0.0783  [2.96]  0.6628  0.0556  0.9936  <.0001  0.8044 0.9110
## 169 -0.0558  0.3400  0.4183  [2.54]  0.9252  0.9891  <.0001  1.0000 0.9999
## 221  0.2442  0.6400  0.7183  0.3000  [2.24]  0.3757  <.0001  0.8309 0.6914
## 246 -0.2733  0.1225  0.2008 -0.2175 -0.5175  [2.76]  <.0001  0.9984 0.9999
## 339  1.3925  1.7883  1.8667  1.4483  1.1483  1.6658  [1.09]  <.0001 <.0001
## 442 -0.1092  0.2867  0.3650 -0.0533 -0.3533  0.1642 -1.5017  [2.59] 1.0000
## 492 -0.1642  0.2317  0.3100 -0.1083 -0.4083  0.1092 -1.5567 -0.0550 [2.65]
##
## Row and column labels: Uncertainty
## Upper triangle: P values   adjust = "tukey"
## Diagonal: [Estimates] (emmean)   type = "response"
## Lower triangle: Comparisons (estimate)   earlier vs. later

pwpp(emm$emmeans)
```

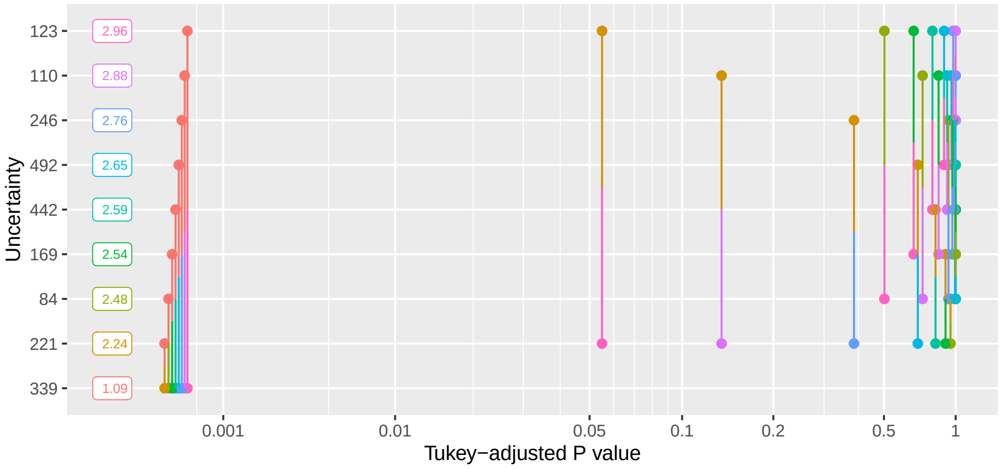

4 Reaction times

For comparison with d' results

```
boxplot(RT~Uncertainty, data=rt.wos,
main="Experiment III", xlab="Uncertainty (nats)")
```

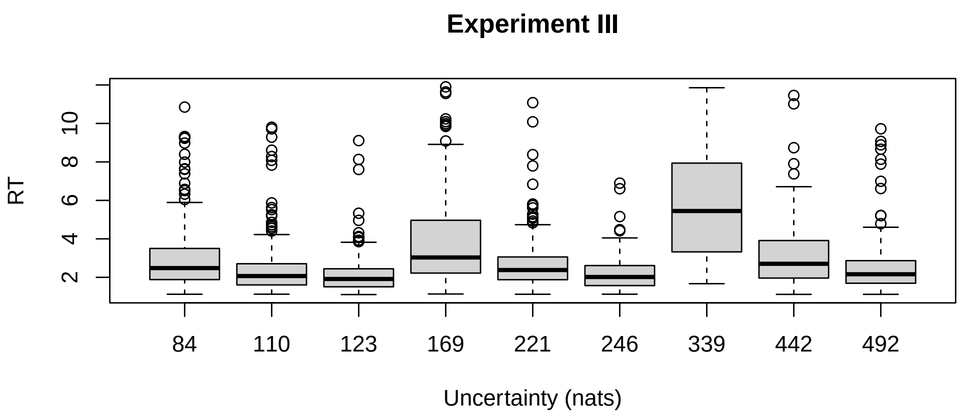


```
bwplot(RT~Uncertainty|T.Rate, data=rt.wos, layout=c(3,1))
```

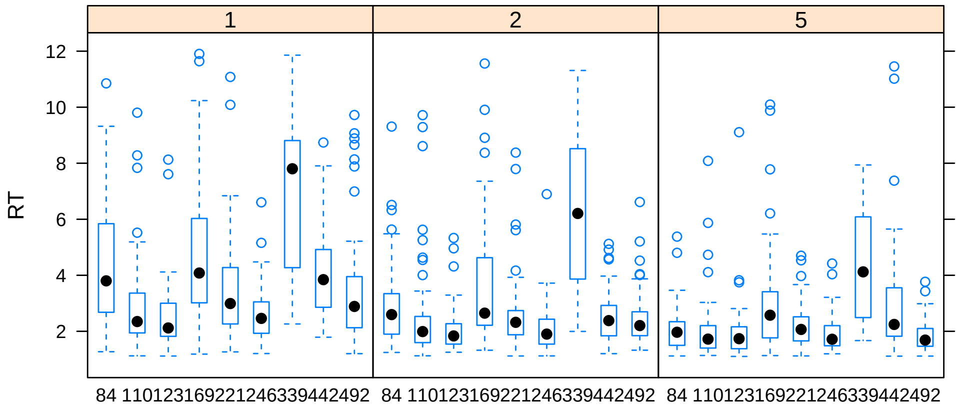


```
bwplot(RT~T.Rate|Uncertainty, data=rt.wos, layout=c(3,3))
```

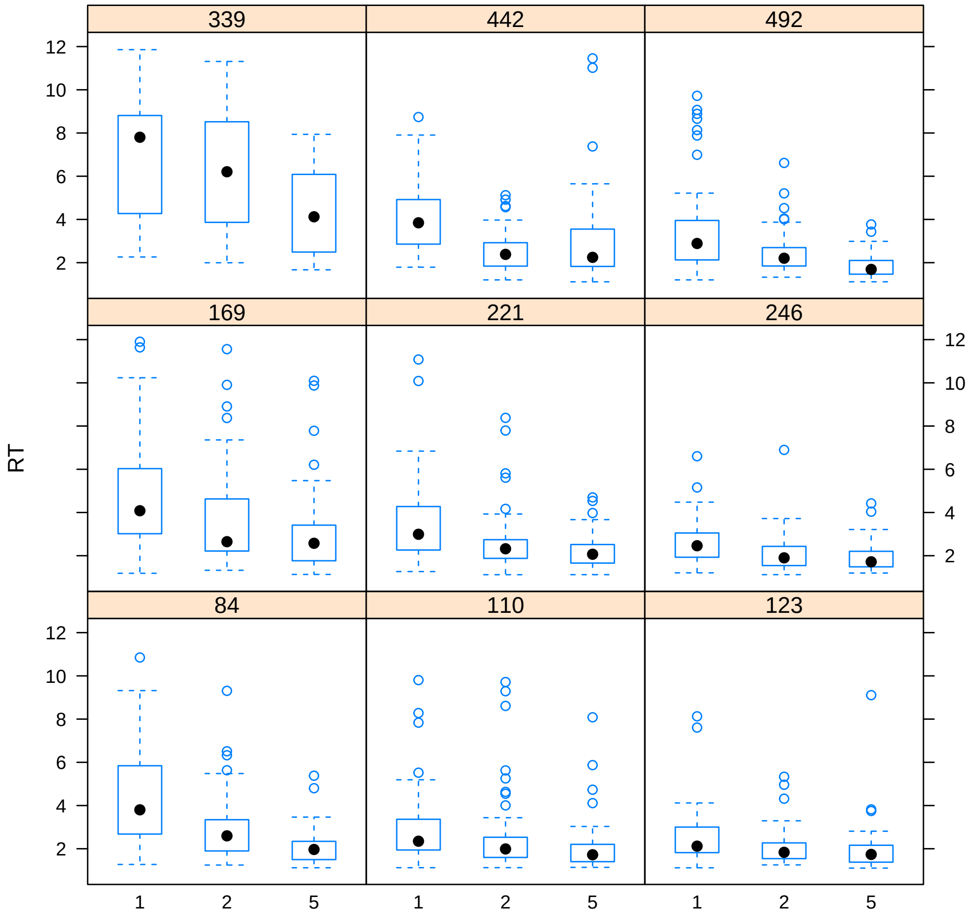

4.1 Time-to-event analysis

Creation of the survival object

```
tte.wos <- rbind(tte.wos[which(tte.wos$RT > 1.1 & tte.wos$Hits == 1),],
                 tte.wos[which(tte.wos$Miss == 1),])
summary(tte.wos)

##       Sujet          Bloc        Stim                Hits            FA
##  1      :135   Min.   :2   Length:1597        Min.   :0.00   Min.   :0
##  3      :135   1st Qu.:3   Class :character   1st Qu.:1.00   1st Qu.:0
##  4      :135   Median :4   Mode  :character   Median :1.00   Median :0
##  5      :135   Mean   :4                      Mean   :0.88   Mean   :0
##  6      :135   3rd Qu.:5                      3rd Qu.:1.00   3rd Qu.:0
##  10     :135   Max.   :6                      Max.   :1.00   Max.   :0
##  (Other):787
##       Miss            RC          RT            Diss         m_ppo
##  Min.   :0.00   Min.   :0   Min.   : 0.0   Min.   :40   Min.   :16
##  1st Qu.:0.00   1st Qu.:0   1st Qu.: 1.5   1st Qu.:40   1st Qu.:16
##  Median :0.00   Median :0   Median : 2.1   Median :40   Median :32
##  Mean   :0.12   Mean   :0   Mean   : 2.6   Mean   :40   Mean   :38
##  3rd Qu.:0.00   3rd Qu.:0   3rd Qu.: 3.1   3rd Qu.:40   3rd Qu.:64
##  Max.   :1.00   Max.   :0   Max.   :11.9   Max.   :40   Max.   :64
```

```
##
##       m_td          m_iti          t_pi       T.Rate     t_td
##  Min.   :0.02   Min.   : 300   Min.   : 489   1:543   Min.   :0.06
##  1st Qu.:0.02   1st Qu.: 300   1st Qu.: 699   2:532   1st Qu.:0.06
##  Median :0.02   Median :1100   Median :1000   5:522   Median :0.06
##  Mean   :0.02   Mean   :1224   Mean   :1425           Mean   :0.06
##  3rd Qu.:0.02   3rd Qu.:2300   3rd Qu.:2045           3rd Qu.:0.06
##  Max.   :0.02   Max.   :2300   Max.   :2924           Max.   :0.06
##
##    m_density      Uncertainty
##  Min.   : 4.0   169    :183
##  1st Qu.: 6.0   221    :183
##  Median : 9.0   492    :183
##  Mean   :11.4   84     :181
##  3rd Qu.:17.0   339    :181
##  Max.   :23.0   123    :175
##                 (Other):511
```

```
# ADD THIS!
tte.wos$RT[which(tte.wos$Miss == 1)] <- 12

survie = Surv(tte.wos$RT, tte.wos$Hits)
model.wf <- coxph(survie ~ Uncertainty * T.Rate +
                      frailty(Sujet, distribution='gaussian'), data=tte.wos)
plot.cox.diagnostics(model.wf, tte.wos)
```

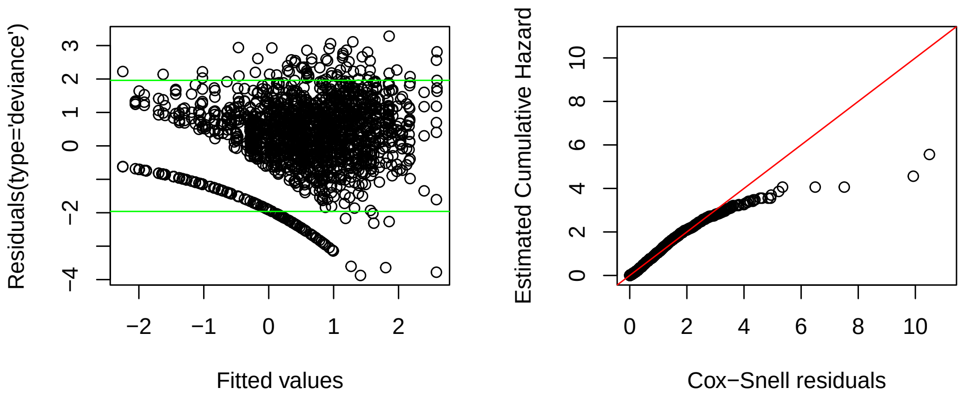


```
plot.cox.influence(model.wf, tte.wos)
```

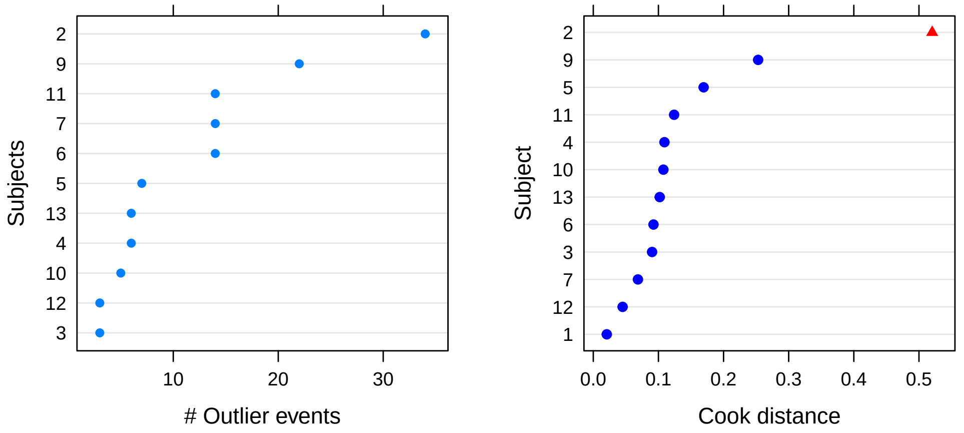

The total number of events (hits) is: 1406 and the average number of event per subject is 117.17 and the average number of event per modality is 52.07.

The total number of events (hits) is: 1406.

```
summary(model.wf)

## Call:
## coxph(formula = survie ~ Uncertainty * T.Rate + frailty(Sujet,
##     distribution = "gaussian"), data = tte.wos)
##
##   n= 1597, number of events= 1406
##
##                             coef    se(coef) se2   Chisq  DF   p
## Uncertainty110               0.7510 0.188    0.188  15.99  1.0 6.4e-05
## Uncertainty123               0.8308 0.189    0.189  19.23  1.0 1.2e-05
## Uncertainty169              -0.2980 0.193    0.193   2.39  1.0 1.2e-01
## Uncertainty221               0.2434 0.190    0.190   1.65  1.0 2.0e-01
## Uncertainty246               0.7110 0.193    0.193  13.61  1.0 2.2e-04
## Uncertainty339              -1.7181 0.267    0.267  41.45  1.0 1.2e-10
## Uncertainty442              -0.3084 0.199    0.199   2.40  1.0 1.2e-01
## Uncertainty492               0.1572 0.188    0.188   0.70  1.0 4.0e-01
## T.Rate2                      0.5693 0.187    0.187   9.31  1.0 2.3e-03
## T.Rate5                      1.3657 0.192    0.191  50.84  1.0 1.0e-12
## frailty(Sujet, distributi                          156.34 11.2 9.4e-28
## Uncertainty110:T.Rate2      -0.5646 0.266    0.266   4.50  1.0 3.4e-02
## Uncertainty123:T.Rate2      -0.1539 0.265    0.265   0.34  1.0 5.6e-01
## Uncertainty169:T.Rate2      -0.1990 0.272    0.272   0.54  1.0 4.6e-01
## Uncertainty221:T.Rate2      -0.1008 0.265    0.265   0.14  1.0 7.0e-01
## Uncertainty246:T.Rate2       0.0318 0.267    0.267   0.01  1.0 9.1e-01
## Uncertainty339:T.Rate2       0.0519 0.345    0.345   0.02  1.0 8.8e-01
## Uncertainty442:T.Rate2       0.0304 0.277    0.277   0.01  1.0 9.1e-01
## Uncertainty492:T.Rate2       0.0564 0.265    0.265   0.05  1.0 8.3e-01
## Uncertainty110:T.Rate5      -0.9550 0.278    0.278  11.77  1.0 6.0e-04
## Uncertainty123:T.Rate5      -0.4164 0.268    0.268   2.42  1.0 1.2e-01
## Uncertainty169:T.Rate5      -0.7413 0.270    0.270   7.51  1.0 6.1e-03
## Uncertainty221:T.Rate5      -0.5098 0.266    0.266   3.66  1.0 5.6e-02
## Uncertainty246:T.Rate5      -0.4942 0.271    0.271   3.32  1.0 6.9e-02
## Uncertainty339:T.Rate5      -0.4996 0.343    0.343   2.12  1.0 1.5e-01
## Uncertainty442:T.Rate5      -0.5730 0.278    0.278   4.26  1.0 3.9e-02
## Uncertainty492:T.Rate5       0.2482 0.265    0.265   0.88  1.0 3.5e-01
```

```
##
##                          exp(coef) exp(-coef) lower .95 upper .95
## Uncertainty110               2.119      0.472     1.467     3.062
## Uncertainty123               2.295      0.436     1.583     3.327
## Uncertainty169               0.742      1.347     0.509     1.083
## Uncertainty221               1.276      0.784     0.880     1.850
## Uncertainty246               2.036      0.491     1.396     2.971
## Uncertainty339               0.179      5.574     0.106     0.303
## Uncertainty442               0.735      1.361     0.497     1.085
## Uncertainty492               1.170      0.855     0.809     1.693
## T.Rate2                      1.767      0.566     1.226     2.547
## T.Rate5                      3.918      0.255     2.692     5.703
## Uncertainty110:T.Rate2       0.569      1.759     0.338     0.958
## Uncertainty123:T.Rate2       0.857      1.166     0.510     1.443
## Uncertainty169:T.Rate2       0.820      1.220     0.481     1.396
## Uncertainty221:T.Rate2       0.904      1.106     0.538     1.520
## Uncertainty246:T.Rate2       1.032      0.969     0.611     1.743
## Uncertainty339:T.Rate2       1.053      0.949     0.536     2.069
## Uncertainty442:T.Rate2       1.031      0.970     0.600     1.773
## Uncertainty492:T.Rate2       1.058      0.945     0.630     1.778
## Uncertainty110:T.Rate5       0.385      2.599     0.223     0.664
## Uncertainty123:T.Rate5       0.659      1.516     0.390     1.114
## Uncertainty169:T.Rate5       0.477      2.099     0.280     0.810
## Uncertainty221:T.Rate5       0.601      1.665     0.356     1.013
## Uncertainty246:T.Rate5       0.610      1.639     0.358     1.038
## Uncertainty339:T.Rate5       0.607      1.648     0.310     1.189
## Uncertainty442:T.Rate5       0.564      1.774     0.327     0.972
## Uncertainty492:T.Rate5       1.282      0.780     0.762     2.155
##
## Iterations: 6 outer, 19 Newton-Raphson
##      Variance of random effect= 0.13
## Degrees of freedom for terms=  8.0  2.0 11.2 16.0
## Concordance= 0.739  (se = 0.006 )
## Likelihood ratio test= 811  on 37.1 df,   p=<2e-16
```

```
anova(model.wf)

## Analysis of Deviance Table
##  Cox model: response is survie
## Terms added sequentially (first to last)
##
##                                             loglik  Chisq   Df Pr(>|Chi|)
## NULL                                         -9371
## Uncertainty                                  -9142 458.32  8.0     <2e-16 ***
## T.Rate                                       -9065 153.81  2.0     <2e-16 ***
## frailty(Sujet, distribution = "gaussian")  -9064   1.25  1.0       0.26
## Uncertainty:T.Rate                           -8966 197.78 26.1     <2e-16 ***
## ---
## Signif. codes:  0 '***' 0.001 '**' 0.01 '*' 0.05 '.' 0.1 ' ' 1
```

```
emmip(model.wf, Uncertainty ~ T.Rate, engine='lattice')
```

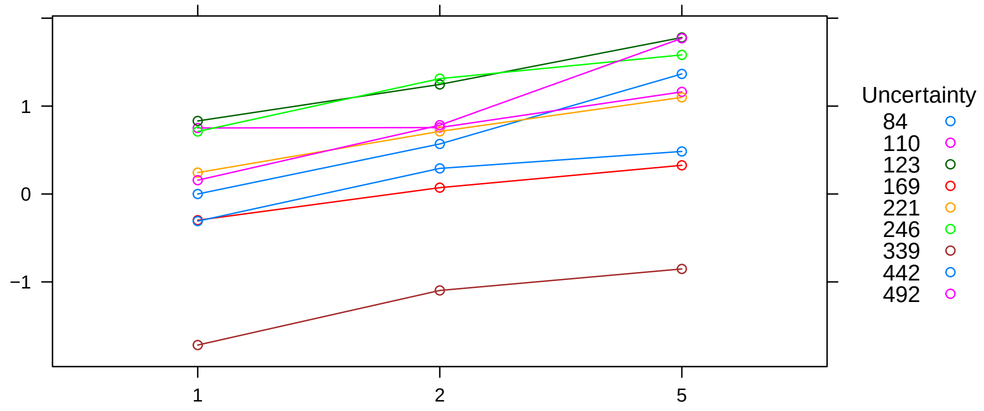


```
emmip(model.wf, T.Rate ~ Uncertainty, engine='lattice')
```

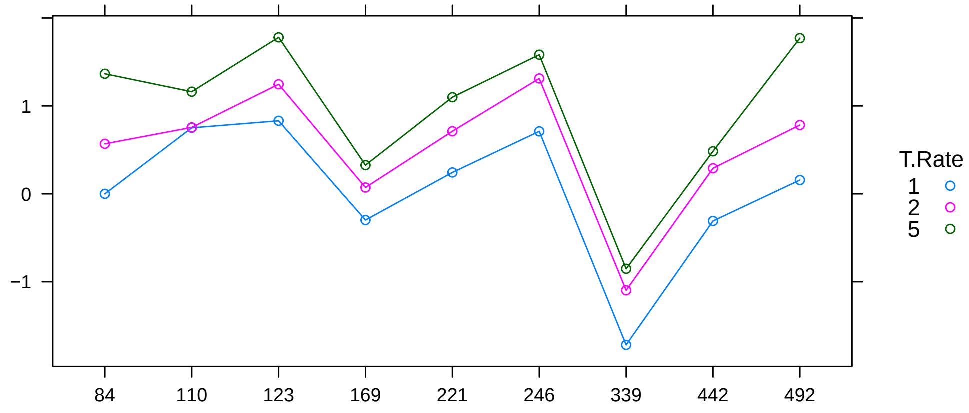


```
emm <- emmeans(model.wf, specs=pairwise~T.Rate:Uncertainty)
pwpm(emm$emmeans)

##              1 84      2 84      5 84     1 110     2 110     5 110     1 123
## 1 84   [ 0.0000]    0.3043    <.0001    0.0168    0.0235    <.0001    0.0035
## 2 84   -0.569305 [ 0.5693]    0.0052    1.0000    1.0000    0.3549    0.9998
## 5 84   -1.365660 -0.796356 [ 1.3657]    0.1627    0.2291    1.0000    0.4569
## 1 110  -0.751013 -0.181708  0.614647 [ 0.7510]    1.0000    0.9556    1.0000
## 2 110  -0.755742 -0.186437  0.609918 -0.004729 [ 0.7557]    0.9708    1.0000
## 5 110  -1.161632 -0.592328  0.204028 -0.410619 -0.405890 [ 1.1616]    0.9976
## 1 123  -0.830849 -0.261544  0.534812 -0.079835 -0.075107  0.330784 [ 0.8308]
## 2 123  -1.246270 -0.676965  0.119391 -0.495257 -0.490528 -0.084637 -0.415421
## 5 123  -1.780148 -1.210843 -0.414488 -1.029135 -1.024406 -0.618516 -0.949299
## 1 169   0.298026  0.867331  1.663686  1.049039  1.053768  1.459658  1.128875
## 2 169  -0.072307  0.496998  1.293353  0.678706  0.683435  1.089325  0.758542
## 5 169  -0.326376  0.242929  1.039284  0.424637  0.429366  0.835256  0.504472
## 1 221  -0.243450  0.325855  1.122211  0.507563  0.512292  0.918183  0.587399
## 2 221  -0.711958 -0.142653  0.653703  0.039055  0.043784  0.449675  0.118891
```

```
## 5 221 -1.099319 -0.530014  0.266342 -0.348306 -0.343577  0.062313 -0.268470
## 1 246 -0.711013 -0.141708  0.654647  0.040000  0.044729  0.450619  0.119836
## 2 246 -1.312168 -0.742863  0.053493 -0.561155 -0.556426 -0.150535 -0.481319
## 5 246 -1.582521 -1.013216 -0.216860 -0.831508 -0.826779 -0.420888 -0.751672
## 1 339  1.718117  2.287422  3.083777  2.469130  2.473859  2.879749  2.548966
## 2 339  1.096943  1.666248  2.462603  1.847956  1.852685  2.258575  1.927791
## 5 339  0.852075  1.421380  2.217736  1.603089  1.607817  2.013708  1.682924
## 1 442  0.308407  0.877712  1.674067  1.059420  1.064149  1.470039  1.139256
## 2 442 -0.291347  0.277958  1.074313  0.459666  0.464395  0.870285  0.539501
## 5 442 -0.484204  0.085101  0.881456  0.266809  0.271538  0.677428  0.346644
## 1 492 -0.157228  0.412077  1.208432  0.593785  0.598514  1.004404  0.673621
## 2 492 -0.782901 -0.213596  0.582760 -0.031888 -0.027159  0.378732  0.047948
## 5 492 -1.771134 -1.201829 -0.405474 -1.020121 -1.015392 -0.609502 -0.940286
##            2 123     5 123     1 169     2 169     5 169     1 221     2 221
## 1 84      <.0001    <.0001    0.9991    1.0000    0.9956    1.0000    0.0449
## 2 84      0.0628    <.0001    0.0013    0.6635    1.0000    0.9935    1.0000
## 5 84      1.0000    0.9137    <.0001    <.0001    <.0001    <.0001    0.1093
## 1 110     0.6155    <.0001    <.0001    0.0868    0.8731    0.5439    1.0000
## 2 110     0.6976    <.0001    <.0001    0.1101    0.8946    0.5949    1.0000
## 5 110     1.0000    0.3036    <.0001    <.0001    0.0100    0.0016    0.9010
## 1 123     0.9071    0.0002    <.0001    0.0239    0.5933    0.2516    1.0000
## 2 123 [ 1.2463]     0.4830    <.0001    <.0001    0.0005    <.0001    0.4812
## 5 123 -0.533878 [ 1.7801]    <.0001    <.0001    <.0001    <.0001    <.0001
## 1 169  1.544296  2.078174 [-0.2980]    0.9852    0.1908    0.4721    <.0001
## 2 169  1.173963  1.707841 -0.370333 [ 0.0723]    1.0000    1.0000    0.1811
## 5 169  0.919893  1.453772 -0.624402 -0.254069 [ 0.3264]    1.0000    0.9606
## 1 221  1.002820  1.536698 -0.541476 -0.171143  0.082927 [ 0.2434]    0.7462
## 2 221  0.534312  1.068190 -1.009984 -0.639651 -0.385581 -0.468508 [ 0.7120]
## 5 221  0.146951  0.680829 -1.397345 -1.027012 -0.772943 -0.855869 -0.387361
## 1 246  0.535257  1.069135 -1.009039 -0.638706 -0.384637 -0.467563  0.000945
## 2 246 -0.065898  0.467980 -1.610194 -1.239861 -0.985792 -1.068718 -0.600210
## 5 246 -0.336251  0.197627 -1.880547 -1.510214 -1.256144 -1.339071 -0.870563
## 1 339  2.964387  3.498265  1.420091  1.790424  2.044493  1.961567  2.430075
## 2 339  2.343213  2.877091  0.798917  1.169250  1.423319  1.340393  1.808901
## 5 339  2.098345  2.632223  0.554049  0.924382  1.178452  1.095525  1.564033
## 1 442  1.554677  2.088555  0.010381  0.380714  0.634783  0.551857  1.020365
## 2 442  0.954923  1.488801 -0.589373 -0.219040  0.035029 -0.047897  0.420611
## 5 442  0.762066  1.295944 -0.782230 -0.411897 -0.157828 -0.240754  0.227754
## 1 492  1.089042  1.622920 -0.455254 -0.084921  0.169148  0.086222  0.554730
## 2 492  0.463369  0.997247 -1.080927 -0.710594 -0.456525 -0.539451 -0.070943
## 5 492 -0.524864  0.009014 -2.069160 -1.698827 -1.444758 -1.527684 -1.059176
##            5 221     1 246     2 246     5 246     1 339     2 339     5 339
## 1 84      <.0001    0.0512    <.0001    <.0001    <.0001    0.0003    0.0218
## 2 84      0.4209    1.0000    0.0179    <.0001    <.0001    <.0001    <.0001
## 5 84      0.9998    0.1164    1.0000    1.0000    <.0001    <.0001    <.0001
## 1 110     0.9833    1.0000    0.3390    0.0030    <.0001    <.0001    <.0001
## 2 110     0.9911    1.0000    0.4269    0.0060    <.0001    <.0001    <.0001
## 5 110     1.0000    0.9061    1.0000    0.9456    <.0001    <.0001    <.0001
## 1 123     0.9997    1.0000    0.6924    0.0192    <.0001    <.0001    <.0001
## 2 123     1.0000    0.4989    1.0000    0.9938    <.0001    <.0001    <.0001
## 5 123     0.0634    <.0001    0.7597    1.0000    <.0001    <.0001    <.0001
## 1 169     <.0001    <.0001    <.0001    <.0001    <.0001    0.0775    0.7024
## 2 169     <.0001    0.1977    <.0001    <.0001    <.0001    <.0001    0.0081
## 5 169     0.0107    0.9652    <.0001    <.0001    <.0001    <.0001    <.0001
## 1 221     0.0015    0.7646    <.0001    <.0001    <.0001    <.0001    0.0001
## 2 221     0.9517    1.0000    0.2379    0.0017    <.0001    <.0001    <.0001
```

```
## 5 221 [ 1.0993]    0.9556    1.0000    0.6804    <.0001    <.0001    <.0001
## 1 246  0.388306 [ 0.7110]    0.2581    0.0020    <.0001    <.0001    <.0001
## 2 246 -0.212849 -0.601155 [ 1.3122]    0.9998    <.0001    <.0001    <.0001
## 5 246 -0.483202 -0.871508 -0.270353 [ 1.5825]    <.0001    <.0001    <.0001
## 1 339  2.817436  2.429130  3.030285  3.300638 [-1.7181]    0.9286    0.3143
## 2 339  2.196262  1.807956  2.409111  2.679463 -0.621174 [-1.0969]    1.0000
## 5 339  1.951394  1.563088  2.164243  2.434596 -0.866042 -0.244867 [-0.8521]
## 1 442  1.407726  1.019420  1.620575  1.890928 -1.409710 -0.788536 -0.543668
## 2 442  0.807972  0.419666  1.020821  1.291174 -2.009464 -1.388290 -1.143423
## 5 442  0.615115  0.226809  0.827964  1.098317 -2.202321 -1.581147 -1.336279
## 1 492  0.942091  0.553785  1.154940  1.425293 -1.875345 -1.254171 -1.009303
## 2 492  0.316418 -0.071888  0.529267  0.799620 -2.501018 -1.879844 -1.634976
## 5 492 -0.671815 -1.060121 -0.458966 -0.188613 -3.489251 -2.868077 -2.623210
##           1 442     2 442     5 442     1 492     2 492     5 492
## 1 84     0.9991    0.9996    0.7385    1.0000    0.0116    <.0001
## 2 84     0.0021    0.9997    1.0000    0.8904    1.0000    <.0001
## 5 84     <.0001    <.0001    0.0017    <.0001    0.2867    0.9180
## 1 110    <.0001    0.8222    0.9998    0.2141    1.0000    <.0001
## 2 110    <.0001    0.8479    0.9999    0.2524    1.0000    <.0001
## 5 110    <.0001    0.0092    0.1714    0.0002    0.9862    0.3286
## 1 123    <.0001    0.5219    0.9907    0.0686    1.0000    0.0002
## 2 123    <.0001    0.0005    0.0230    <.0001    0.7761    0.4983
## 5 123    <.0001    <.0001    <.0001    <.0001    <.0001    1.0000
## 1 169    1.0000    0.3659    0.0176    0.8064    <.0001    <.0001
## 2 169    0.9857    1.0000    0.9492    1.0000    0.0617    <.0001
## 5 169    0.2169    1.0000    1.0000    1.0000    0.8018    <.0001
## 1 221    0.5013    1.0000    1.0000    1.0000    0.4466    <.0001
## 2 221    0.0001    0.9308    1.0000    0.3830    1.0000    <.0001
## 5 221    <.0001    0.0095    0.2097    0.0002    0.9966    0.0645
## 1 246    0.0001    0.9404    1.0000    0.4073    1.0000    <.0001
## 2 246    <.0001    <.0001    0.0061    <.0001    0.5053    0.7688
## 5 246    <.0001    <.0001    <.0001    <.0001    0.0085    1.0000
## 1 339    <.0001    <.0001    <.0001    <.0001    <.0001    <.0001
## 2 339    0.1141    <.0001    <.0001    <.0001    <.0001    <.0001
## 5 339    0.7809    <.0001    <.0001    0.0008    <.0001    <.0001
## 1 442 [-0.3084]    0.3939    0.0226    0.8213    <.0001    <.0001
## 2 442 -0.599754 [ 0.2913]    1.0000    1.0000    0.7369    <.0001
## 5 442 -0.792611 -0.192857 [ 0.4842]    0.9961    0.9991    <.0001
## 1 492 -0.465635  0.134119  0.326976 [ 0.1572]    0.1605    <.0001
## 2 492 -1.091308 -0.491554 -0.298697 -0.625673 [ 0.7829]    <.0001
## 5 492 -2.079541 -1.479787 -1.286930 -1.613906 -0.988233 [ 1.7711]
##
## Row and column labels: T.Rate:Uncertainty
## Upper triangle: P values   adjust = "tukey"
## Diagonal: [Estimates] (emmean)
## Lower triangle: Comparisons (estimate)   earlier vs. later

pwpp(emm$emmeans)
```

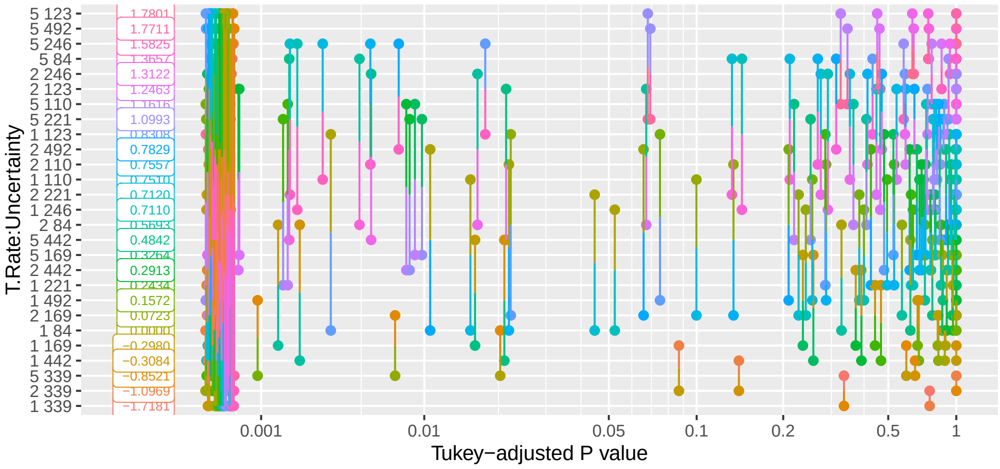


```
# Temporary Table 5
emm.cld <- cld(emm$emmeans)
plot(emm.cld)
```

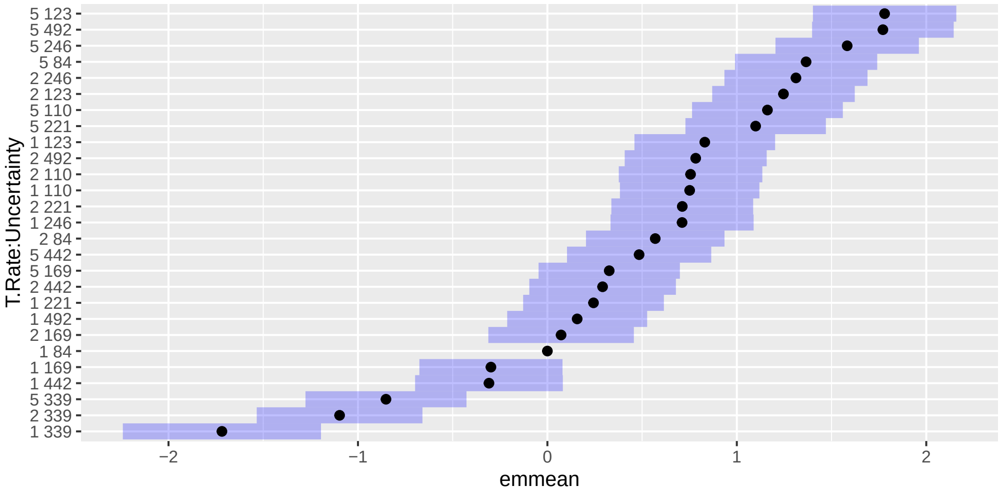


```
emm.cld

##  T.Rate Uncertainty emmean    SE  df asymp.LCL asymp.UCL .group
##  1      339          -1.72 0.267 Inf     -2.24     -1.20  1
##  2      339          -1.10 0.223 Inf     -1.53     -0.66  12
##  5      339          -0.85 0.217 Inf     -1.28     -0.43  12
##  1      442          -0.31 0.199 Inf     -0.70      0.08   23
##  1      169          -0.30 0.193 Inf     -0.68      0.08   23
##  1      84            0.00 0.000 Inf      0.00      0.00    34
##  2      169           0.07 0.196 Inf     -0.31      0.46    345
##  1      492           0.16 0.188 Inf     -0.21      0.53    3456
##  1      221           0.24 0.190 Inf     -0.13      0.61    3456
##  2      442           0.29 0.197 Inf     -0.10      0.68    3456
##  5      169           0.33 0.190 Inf     -0.05      0.70    3456
##  5      442           0.48 0.194 Inf      0.10      0.86     4567
##  2      84            0.57 0.187 Inf      0.20      0.93     45678
##  1      246           0.71 0.193 Inf      0.33      1.09     456789
##  2      221           0.71 0.191 Inf      0.34      1.09      56789
```

```
##  1      110           0.75 0.188 Inf      0.38      1.12      56789
##  2      110           0.76 0.193 Inf      0.38      1.13      56789
##  2      492           0.78 0.191 Inf      0.41      1.16      56789
##  1      123           0.83 0.189 Inf      0.46      1.20       6789
##  5      221           1.10 0.189 Inf      0.73      1.47        7890
##  5      110           1.16 0.203 Inf      0.76      1.56        7890
##  2      123           1.25 0.192 Inf      0.87      1.62         890
##  2      246           1.31 0.193 Inf      0.93      1.69          90
##  5      84            1.37 0.192 Inf      0.99      1.74          90
##  5      246           1.58 0.193 Inf      1.20      1.96           0
##  5      492           1.77 0.191 Inf      1.40      2.14           0
##  5      123           1.78 0.193 Inf      1.40      2.16           0
##
## Results are given on the log (not the response) scale.
## Confidence level used: 0.95
## P value adjustment: tukey method for comparing a family of 27 estimates
## significance level used: alpha = 0.05
## NOTE: If two or more means share the same grouping letter,
##       then we cannot show them to be different.
##       But we also did not show them to be the same.
```

A Check mixed model for d'

```
model.check = lmer(dprime ~ Uncertainty + (1|Sujet),
                   data=perf.all)
(nb.subj <- length(unique(perf.all$Sujet)))

## [1] 13

estex.model.check = influence(model.check, "Sujet")
plot(estex.model.check, which="cook", cutoff=4/nb.subj,
     sort=TRUE, ylab="Subject", xlab="Cook Distance")
```

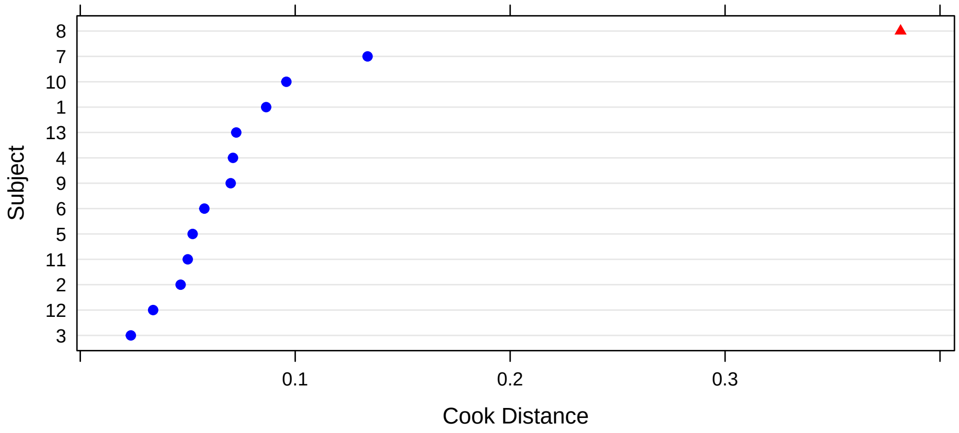

Supplement: S3 File — (PDF) [file pone.0282885.s003.pdf]
