# Supplementary figures and images for: Investigating the influence of masker and target properties on the dynamics of perceptual awareness under informational masking
Source: PLoS One. 2023 Mar 16;18(3):e0282885. doi: 10.1371/journal.pone.0282885 (PMC10019711; doi:10.1371/journal.pone.0282885)

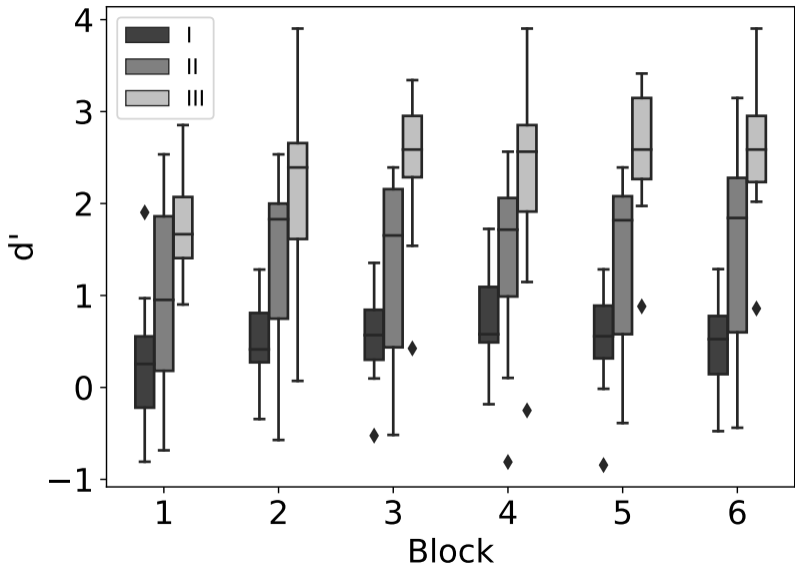

Supplement: S1 Fig — The performance is lower in the first block for the three experiments and thus their results have been removed from final analysis to minimize a residual learning effect. (PDF) [file pone.0282885.s004.pdf]

# Experiment I: Similarity $\times$ Uncertainty

$S = -80$

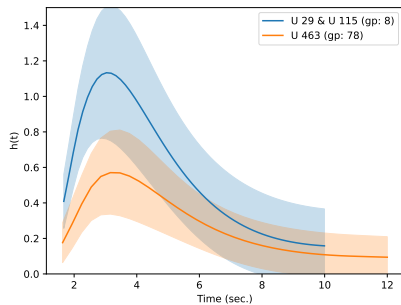

$S = -40$

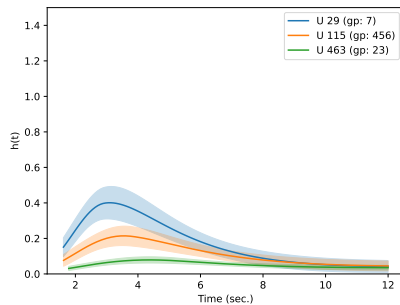

$S = 0$

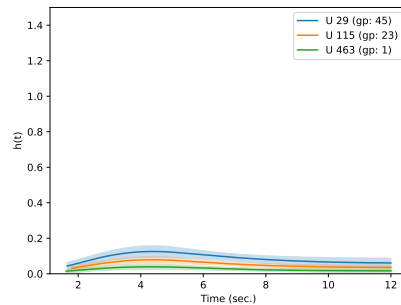

$S = +80$

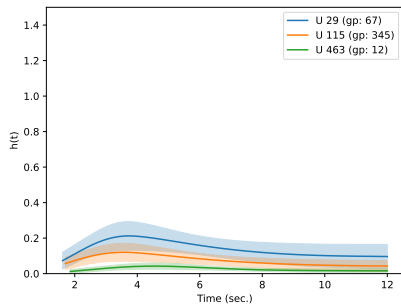

$S = +40$

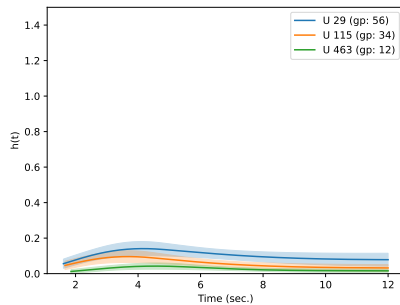

Supplement: S2 Fig — (PDF) [file pone.0282885.s005.pdf]

# Experiment I: Similarity $\times$ Uncertainty

$S = -80$

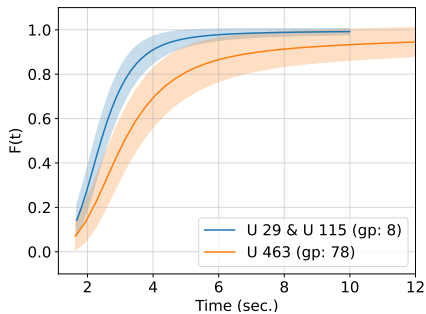

$S = -40$

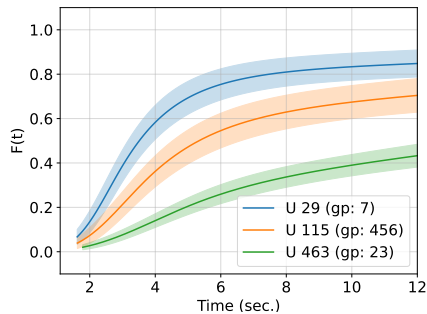

$S = 0$

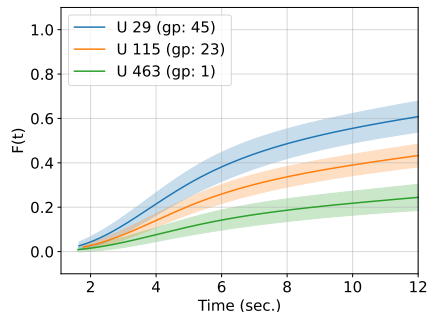

$S = +80$

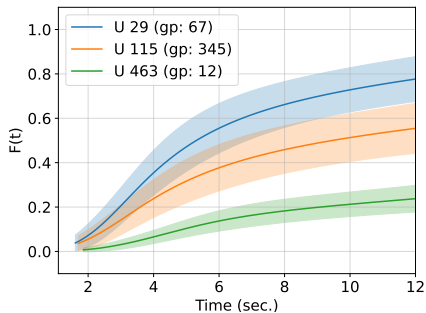

$S = +40$

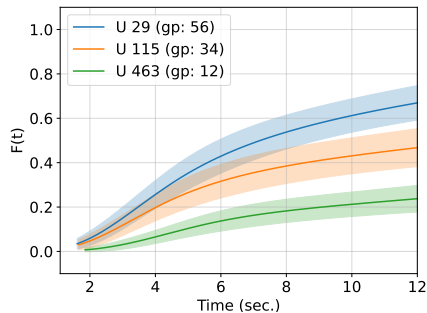

Supplement: S3 Fig — (PDF) [file pone.0282885.s006.pdf]

# Experiment II: Similarity $\times$ Target Rate $\times$ Uncertainty

5 Hz

10 Hz

20 Hz

S0

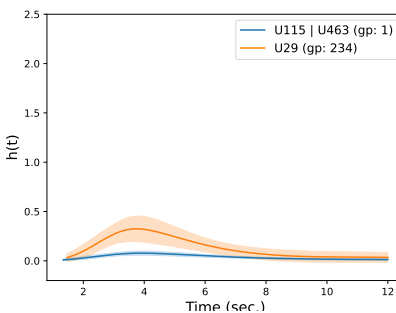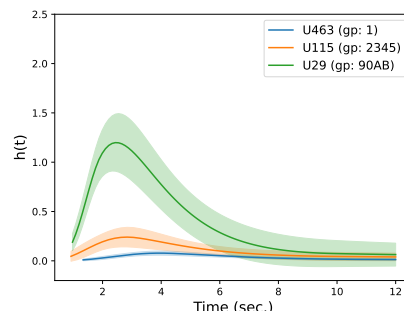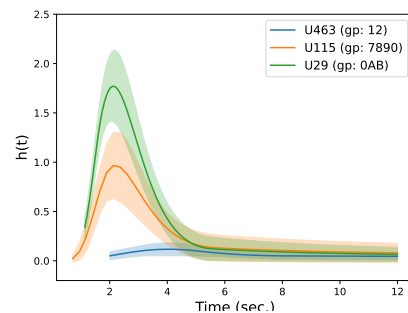

S40

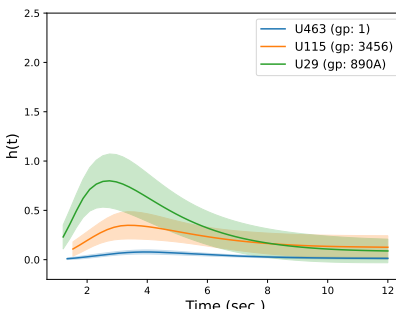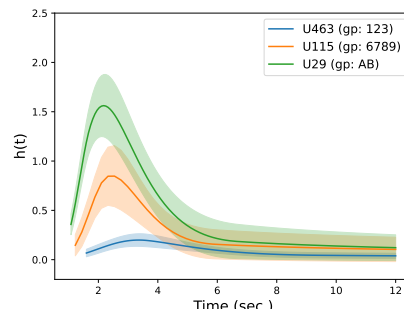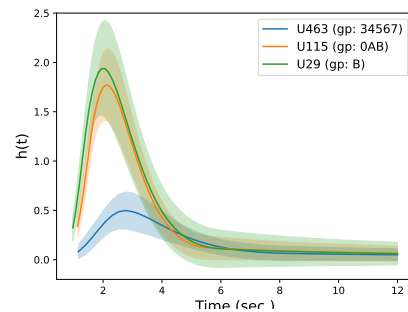

S80

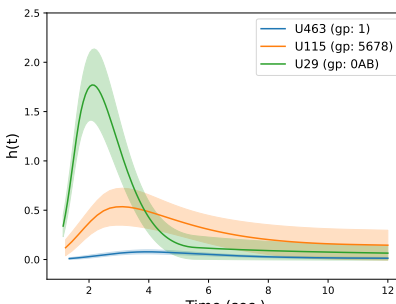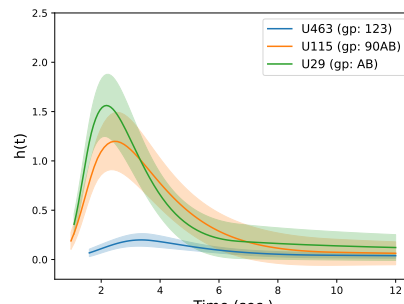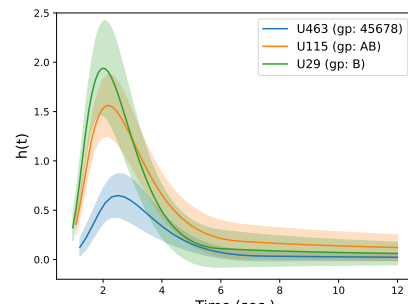

Supplement: S4 Fig — (PDF) [file pone.0282885.s007.pdf]

# Experiment II: Target Rate $\times$ Uncertainty $\times$ Similarity

U = 29 nats

U = 115 nats

U = 463 nats

5 Hz

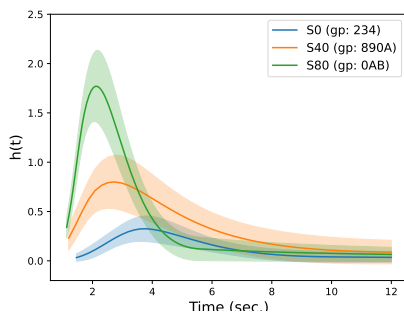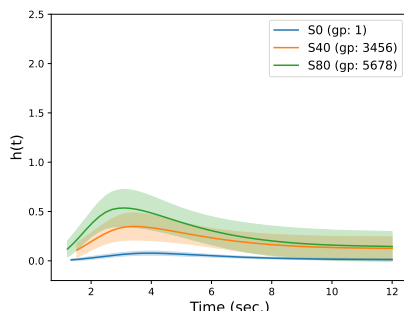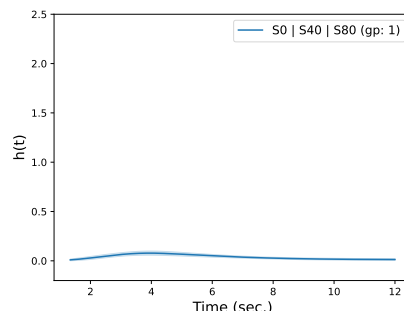

10 Hz

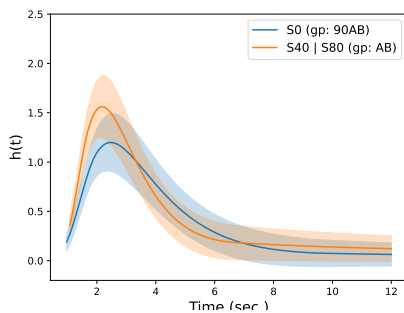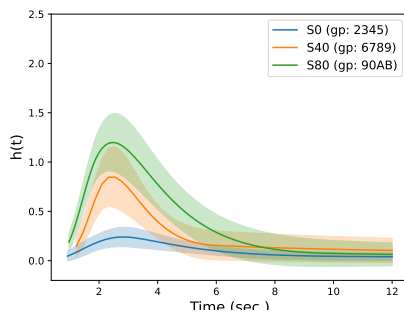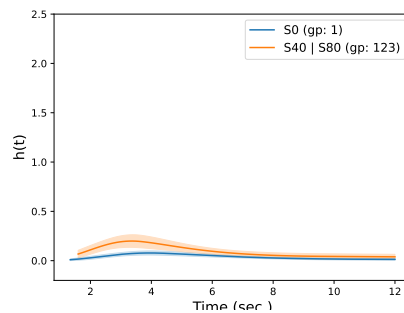

20 Hz

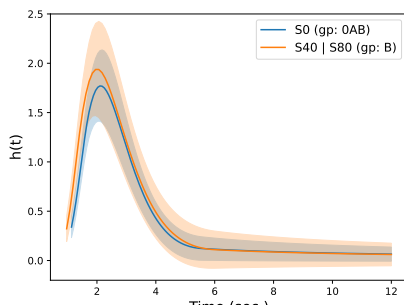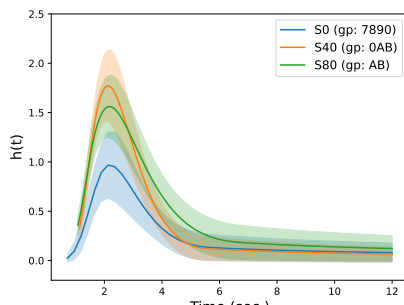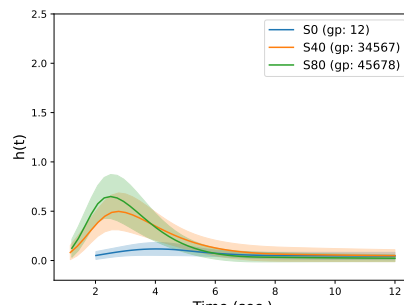

Supplement: S5 Fig — (PDF) [file pone.0282885.s008.pdf]

# Experiment II: Similarity $\times$ Target Rate $\times$ Uncertainty

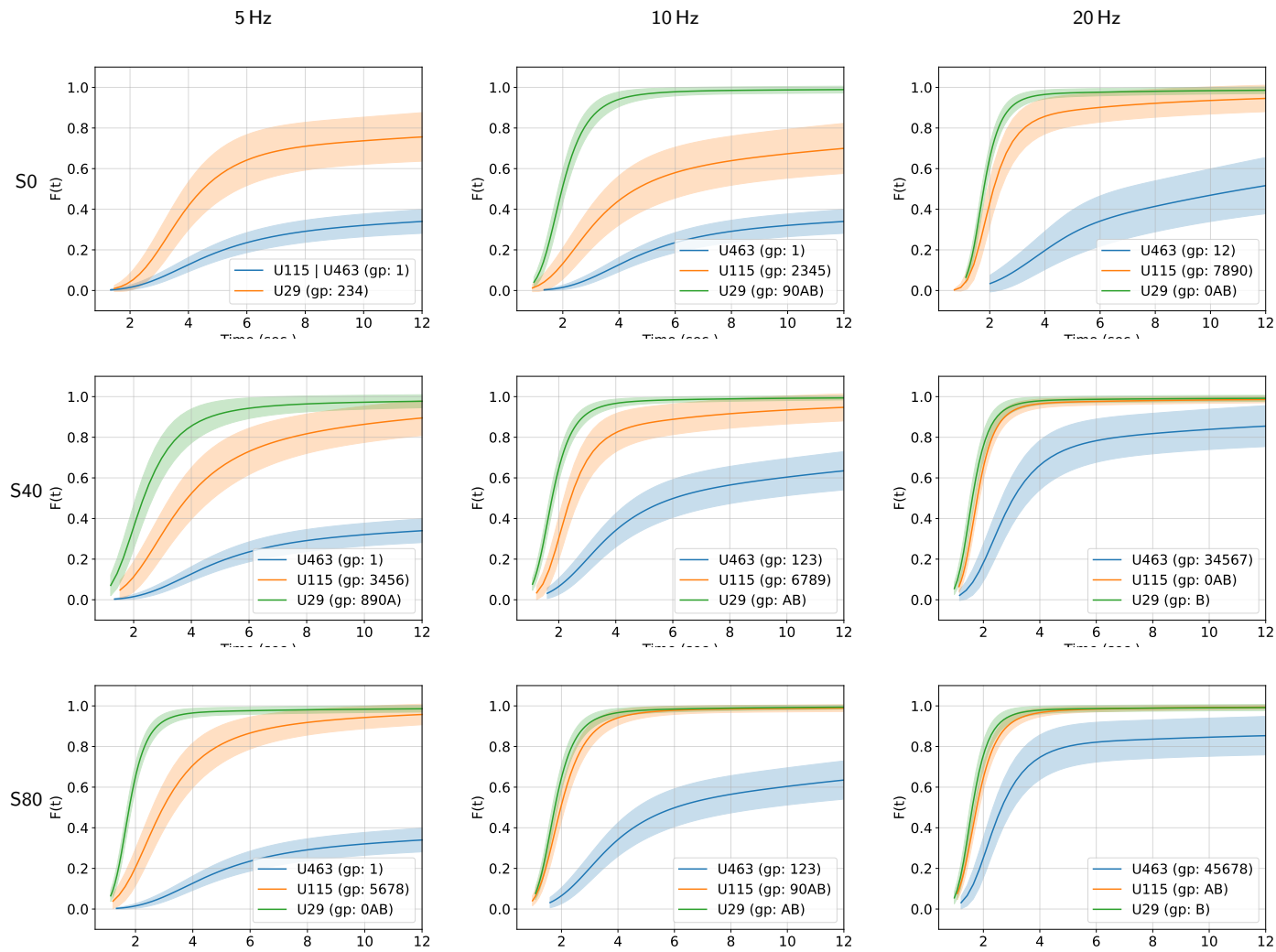

Supplement: S6 Fig — (PDF) [file pone.0282885.s009.pdf]

# Experiment II: Target Rate $\times$ Uncertainty $\times$ Similarity

U = 29 nats

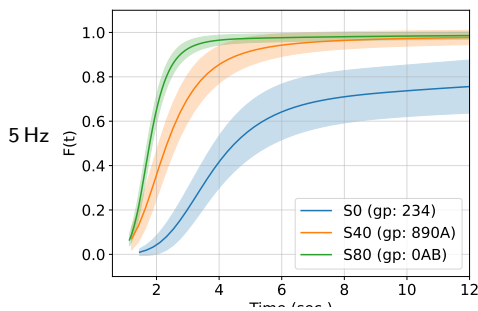

U = 115 nats

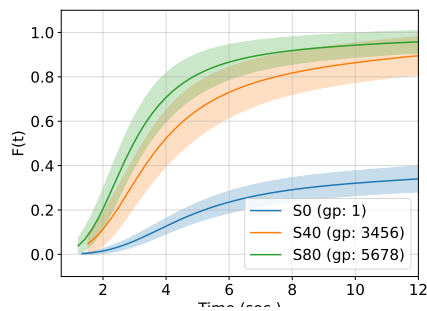

U = 463 nats

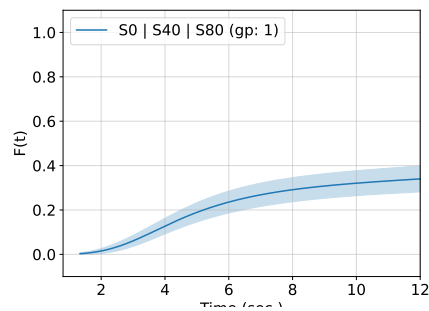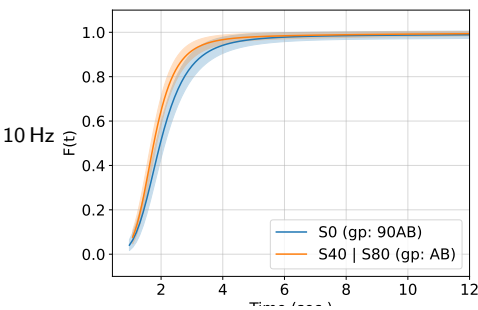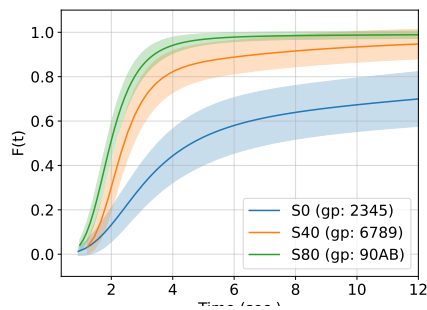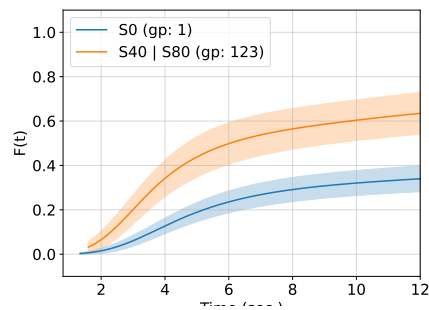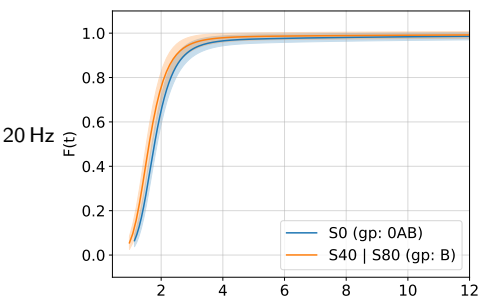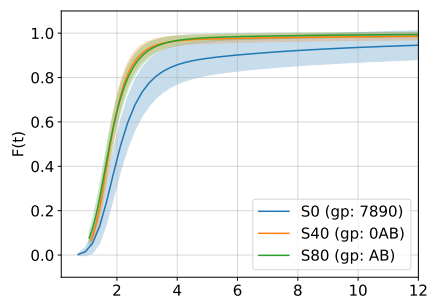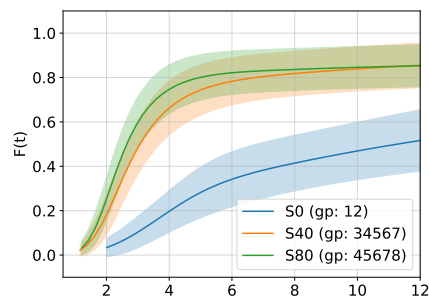

Supplement: S7 Fig — (PDF) [file pone.0282885.s010.pdf]

# Experiment III: Target Rate $\times$ Uncertainty

1 Hz

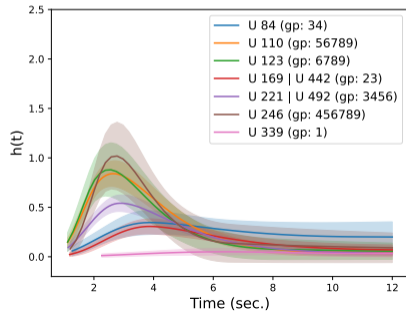

2 Hz

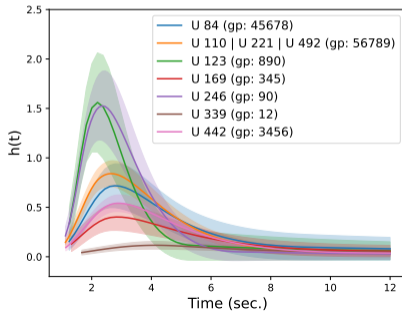

5 Hz

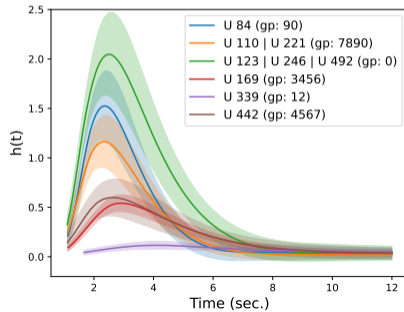

Supplement: S8 Fig — (PDF) [file pone.0282885.s011.pdf]

# Experiment III: Target Rate $\times$ Uncertainty

1 Hz

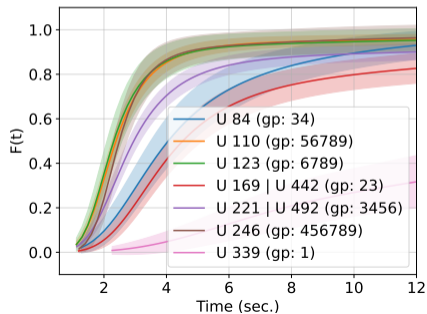

2 Hz

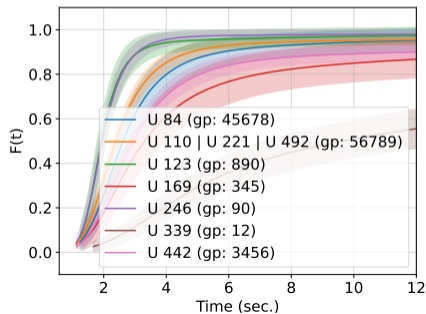

5 Hz

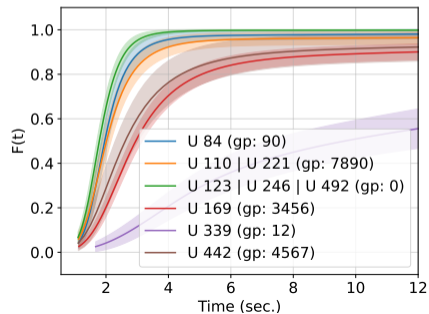

Supplement: S9 Fig — (PDF) [file pone.0282885.s012.pdf]

A.

## Experiment III - Uncertainty main effect

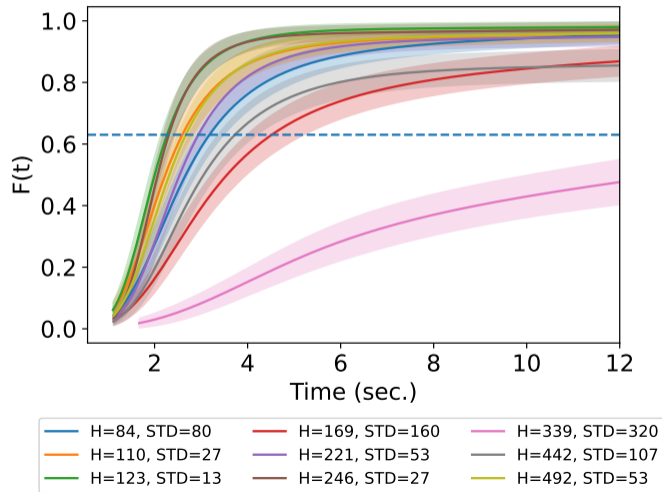

B.

## Entropy vs. Density

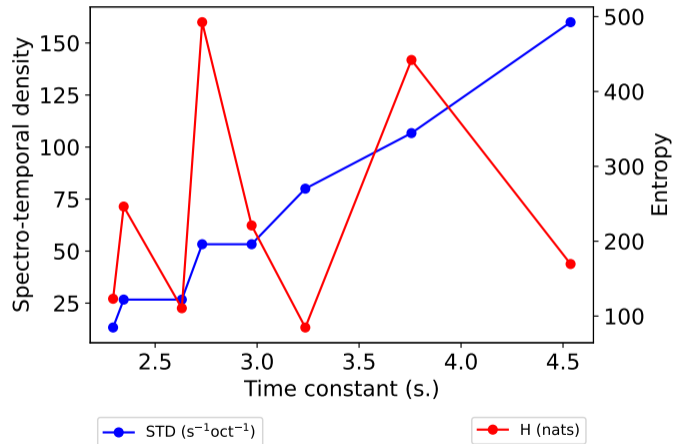

Supplement: S10 Fig — Auditory segregation time constant is defined as the time τ for which the cumulative distribution function associated to the hazard rate for auditory segregation equals 0.63 (see Panel A.). Masker spectro-temporal density is defined as fpo/miti in s−1oct−1 where miti: mean inter-tone interval, fpo: frequencies per octave. Panel B. compares the relationship between τ and STD or entropy. The data where τ > 12 is not depicted. The following table gives the values of masker spectro-temporal density for the set of masker parameters used in the experiments. Exp. I—IIExp. IIImiti (ms)8002006001200Δ (ms)140020010002200fpo45———16—8026.713.3324016053.326.76480320106.753.3 miti: mean inter-tone interval, fpo: frequencies per octave. (PDF) [file pone.0282885.s013.pdf]

**A.**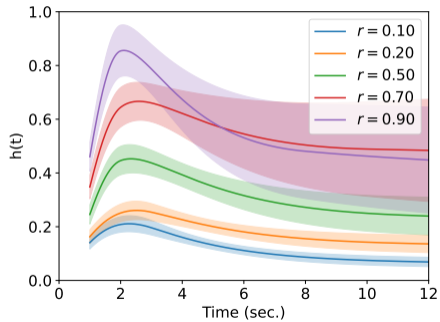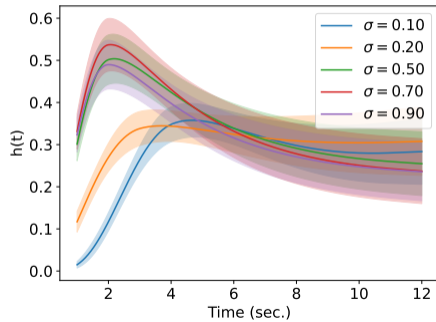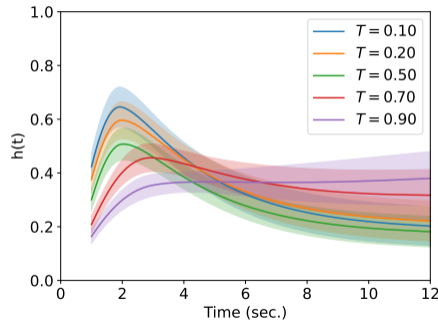

Supplement: S11 Fig — A simple model of evidence accumulation [65] describes activity that accumulates and saturates at target level T. The activity Xn is updated sequentially according to: Xn+1 = Xn + r(T − Xn) + εn+1 where T < 1 and where εn+1∼N(0,σ2) are independent random variables (Gaussian noise of zero mean and standard deviation σ). The activity increments are state dependent and proportional to the difference T − Xn, with constant rate r. Accordingly, the activity X drifts towards T stochastically if 0 < r < 1. Accumulation slows with Xn near T and the activity can cross the threshold only due to noise. Examples of the effect of changes in the model parameters. Constant parameters are: T = 0.9, X0 = 0. When not varied, r = 0.7 and σ = 0.15. (PDF) [file pone.0282885.s014.pdf]
